# Structural Basis of an Asymmetric Condensin ATPase Cycle

**DOI:** 10.1016/j.molcel.2019.03.037

**Published:** 2019-06-20

**Authors:** Markus Hassler, Indra A. Shaltiel, Marc Kschonsak, Bernd Simon, Fabian Merkel, Lena Thärichen, Henry J. Bailey, Jakub Macošek, Sol Bravo, Jutta Metz, Janosch Hennig, Christian H. Haering

**Affiliations:** 1 Cell Biology and Biophysics Unit, European Molecular Biology Laboratory, 69117 Heidelberg, Germany; 2 Structural and Computational Biology Unit, European Molecular Biology Laboratory, 69117 Heidelberg, Germany; 3 Collaboration for Joint PhD degree between EMBL and Heidelberg University, Faculty of Biosciences, Heidelberg, Germany

**Keywords:** condensin, cohesin, SMC protein complex, ABC ATPase, DNA loop extrusion, mitotic chromosome, genome organization, structural biology

## Abstract

The condensin protein complex plays a key role in the structural organization of genomes. How the ATPase activity of its SMC subunits drives large-scale changes in chromosome topology has remained unknown. Here we reconstruct, at near-atomic resolution, the sequence of events that take place during the condensin ATPase cycle. We show that ATP binding induces a conformational switch in the Smc4 head domain that releases its hitherto undescribed interaction with the Ycs4 HEAT-repeat subunit and promotes its engagement with the Smc2 head into an asymmetric heterodimer. SMC head dimerization subsequently enables nucleotide binding at the second active site and disengages the Brn1 kleisin subunit from the Smc2 coiled coil to open the condensin ring. These large-scale transitions in the condensin architecture lay out a mechanistic path for its ability to extrude DNA helices into large loop structures.

## Highlights

• Smc4 and Smc2 ATPase head structures reorganize upon ATP binding and dimerization
• A Q-loop-mediated switch releases the Ycs4 HEAT-repeat subunit from the Smc4 head
• The Smc2 head engages with the ATP-bound Smc4 head into an asymmetric heterodimer
• Head dimerization releases the Brn1 kleisin from Smc2 via coiled-coil rotation

## Graphical Abstract

**Figure:**
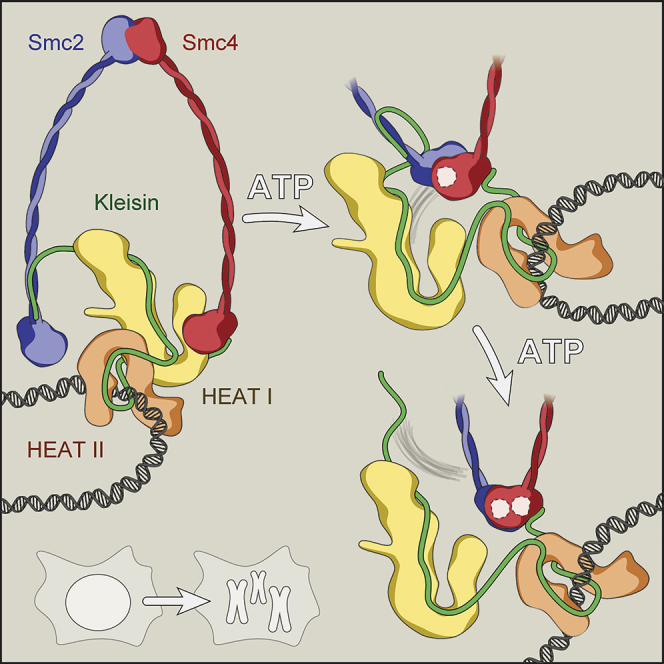

## Teaser

Hassler et al. report structural and functional insights into the enzymatic core of the condensin protein complex that reveal large-scale conformational changes upon ATP binding by and subsequent dimerization of its catalytic SMC head domains. These movements presumably power the condensin-mediated extrusion of DNA loops during mitotic chromosome formation.

## Introduction

Multi-subunit protein complexes of the structural maintenance of chromosomes (SMC) family direct large-scale organizational changes in genome architecture that are essential for all aspects of chromosome biology. In addition to their central functions in the segregation of replicated genomes during prokaryotic and eukaryotic cell divisions (Hirano, 2016, Uhlmann, 2016), SMC protein complexes also determine the three-dimensional landscape of interphase nuclei to regulate gene expression (Albritton and Ercan, 2018, Merkenschlager and Nora, 2016) and contribute to DNA damage repair, recombination, and replication (Wood et al., 2010, Wu and Yu, 2012). The unifying principle for the diverse actions of SMC complexes, foremost condensin and cohesin, might be their ability to extrude DNA into loop structures (Goloborodko et al., 2016, Nasmyth, 2001). This hypothesis is consistent with the recent discoveries that condensin complexes purified from budding yeast are able to translocate along DNA double helices and processively expand loops of DNA of several kilobase pairs in length in a fashion that depends on their ability to hydrolyze ATP (Ganji et al., 2018, Terakawa et al., 2017). How the energy of nucleotide binding, hydrolysis, and release is converted into DNA translocation and looping movements has remained unknown, but currently available models rely on mechanochemical coupling of the ATPase cycle to large conformational transitions that affect chromosome interactions by creating topological compartments or contact sites that entrap or directly bind DNA, respectively (Gruber, 2017, Hassler et al., 2018).

Based on their homology to ATP binding cassette (ABC) transmembrane transporters and the Rad50 DNA damage repair protein (Hopfner, 2016), the two globular ATPase “head” domains situated at the ends of ∼50-nm-long intra-molecular coiled coils of a heterodimer of condensin’s Smc2 and Smc4 subunits are thought to sandwich a pair of ATP molecules between composite catalytic sites, each composed of Walker A (P loop) and Walker B motifs of one head and a so-called ABC signature motif of the opposite head. Each head domain (hd) can be subdivided into a “RecA”-like lobe that contains the ATP-binding pocket and a “helical” lobe that merges into the coiled coils; they connect the heads to a half-doughnut-shaped “hinge” dimerization domain. Smc2_hd_ and Smc4_hd_ are furthermore connected by their binding to opposite ends of the Brn1^Cnd2/NCAPH^ kleisin subunit (Onn et al., 2007). Several crystal structures have revealed the formation of a helical bundle between the kleisin N-terminal domain and the coiled-coil “neck” region immediately adjacent to one SMC head (the ν-SMC_hd_) and the interaction of the kleisin C-terminal winged helix domain (wHD) with the “cap” region located at the distal surface of the other SMC head (the κ-SMC_hd_) of cohesin (Gligoris et al., 2014, Haering et al., 2004) or bacterial SMC complexes (Bürmann et al., 2013, Diebold-Durand et al., 2017, Kamada et al., 2017, Woo et al., 2009). No structural information has so far been available for the homologous ATPase domains or interfaces of the condensin complex. The condensin SMC-kleisin ring structure has been proposed to topologically encircle chromosomal DNA (Cuylen et al., 2011), which is consistent with the finding that other tripartite SMC-kleisin rings that have been covalently circularized retain their association with chromosomal DNA even after protein denaturation (Gligoris et al., 2014, Haering et al., 2008, Wilhelm et al., 2015). Data from cohesin suggest that DNA release from these rings relies on the opening of the interface between the kleisin N terminus and the SMC coiled coil (cc), which serves as a DNA exit (Buheitel and Stemmann, 2013, Chan et al., 2012, Huis in ’t Veld et al., 2014) and, potentially, also as an entry gate (Murayama and Uhlmann, 2015).

The central role of the ATPase cycle to SMC complex function is underscored by the fact that the majority of mutations that affect nucleotide binding, head dimerization, or ATP hydrolysis render cohesin or condensin non-functional (Arumugam et al., 2003, Hudson et al., 2008, Kinoshita et al., 2015, Palou et al., 2018, Thadani et al., 2018, Weitzer et al., 2003). Although these mutations largely abolish the association of cohesin with chromosomes, some of the homologous mutations in condensin have less dramatic effects on the chromosomal levels of condensin. This difference might be due to the presence of an ATPase-independent DNA binding site formed at the interface between the Brn1 kleisin subunit and the Ycg1^Cnd3/NCAPG^ subunit, which is composed of multiple repeats of α-helical HEAT (huntingtin, elongation factor 3, protein phosphatase 2A, Tor1 kinase) motifs (Kschonsak et al., 2017). The function of the second HEAT-repeat subunit, named Ycs4^Cnd1/NCAPD2^, has so far remained unclear, despite its presence being similarly essential for the association of condensin with chromosomes (Kinoshita et al., 2015, Lavoie et al., 2002).

Here we report high-resolution structures of the Smc2 and Smc4 heads, including their interfaces with the N- and C-terminal domains of the Brn1 kleisin subunit, and of the Ycs4-Brn1 complex. We provide evidence for rearrangements of key residues that take place during sequential ATP binding to the two catalytic sites and describe how these structural transitions trigger large-scale conformational changes that result in the dissociation of the Ycs4 subunit from a highly conserved binding site within the Smc4 head, Smc2-Smc4 head dimerization, and, ultimately, release of the Brn1 kleisin subunit from the Smc2 coiled coil.

## Results

### Structural Basis for Asymmetric ATP Binding by the Condensin SMC Head Domains

**Figure 1:**
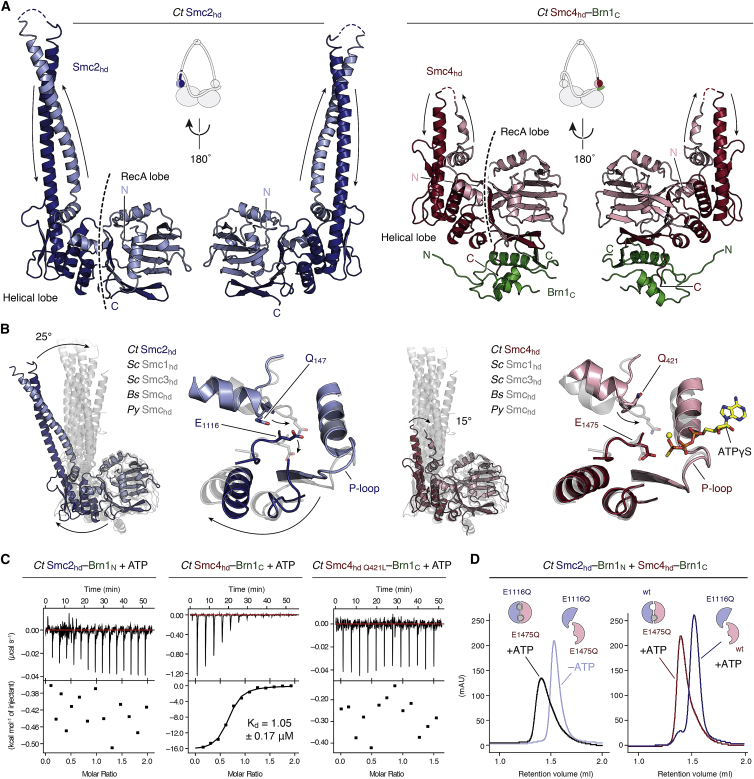
Structures and Dimerization of Smc2 and Smc4 ATPase Head Domains (A) Cartoon models of the *Ct* Smc2_hd_ (crystal form I) and the *Ct* Smc4_hd_-Brn1_C_ complex. (B) Structural alignment based on the RecA-like lobe of *Ct* Smc2_hd_ (I) and *Ct* Smc4_hd_ structures to ATPγS-bound structures of the *Sc* cohesin Smc1_hd_ (PDB: 1W1W; C_α_ root-mean-square deviation [RMSD] = 0.892 and 0.839) and Smc3_hd_ (PDB: 4UX3; C_α_ RMSD = 2.582 and 1.068) or the nucleotide-free structures of *B. subtilis* (*Bs*) SMC_hd_ (PDB: 3ZGX; C_α_ RMSD = 1.828 and 1.914) and *P. yayanosii* (*Py*) SMC_hd_ (PDB: 5XEI; C_α_ RMSD = 0.977 and 0.818). Close-up views highlight the positions of the conserved Q-loop glutamine and Walker B glutamate residues aligned to *Sc* cohesin Smc1_hd_ with ATPγS (gray). (C) Isothermal titration calorimetry (ITC) of ATP binding by WT *Ct* Smc2_hd_-Brn1_N_ and WT or Q-loop mutant *Ct* Smc4_hd_-Brn1_C_ (fit ± error of the fit). (D) Size exclusion chromatography profiles of double or single Walker B mutant combinations of *Ct* Smc2_hd_-Brn1_N_ and *Ct* Smc4_hd_-Brn1_C_ in the absence (–ATP) or presence (+ATP) of nucleotide. See also Figures S1 and S2.

To gain functional insights into the condensin ATPase cycle, we solved the crystal structures of Smc2_hd_ and the Smc4_hd_-Brn1_C_ complex of the thermophilic yeast *Chaetomium thermophilum* (*Ct*; Figure S1A; Table S1) to 2.6- or 3.0-Å resolution, respectively (Figure 1A; Table 1). As expected, both structures revealed canonical two-lobed SMC ATPase folds that display a high degree of evolutionary surface conservation at their ATP-binding and head dimerization interfaces (Figure S1B). Although included in the crystallization construct, crystals of the *Ct* Smc2_hd_ domain showed no electron density for the N-terminal Brn1 region, which presumably dissociated during crystallization (see below). In contrast, the *Ct* Smc4_hd_ crystal structure displayed distinct density for the C-terminal Brn1 region, which folds into a wHD and binds to the cap’ face of the SMC ATPase.

**Table 1:** Crystallography Data Collection and Refinement Statistics

	*Ct* Smc2_hd_ (I) (SeMet- SAD)	*Ct* Smc_2hd_ (II) (Native)	*Ct* Smc4_hd_-Brn1_C_		*Ct* Ycs4-Brn1_Y4_*(SeMet-SIRAS)^∗^*	*Ct* Ycs4-Brn1_Y4_ (Native-SIRAS)	*Ct* Ycs4-Brn1_Y4_-Smc4_hd_-Brn1_C_
**Data collection**							

Space group	P 2_1_ 2_1_ 2_1_	P 6_5_	P 6_4_	P 2_1_		P 2_1_	P 2_1_
Molecules per asymmetric unit	1	1	1	1		1	1

**Cell dimensions (Å,°)**							

a	47.32	93.75	132.72	86.61		86.07	84.40
b	107.98	93.75	132.72	81.76		80.79	82.38
c	174.25	117.97	75.55	132.88		130.84	177.83
α	90.00	90.00	90.00	90.00		90.00	90.00
β	90.00	90.00	90.00	93.15		93.40	98.77
ɣ	90.00	120.00	120.00	90.00		90.00	90.00
Resolution (Å)	45.94–2.50 (2.57–2.50)	81.19–2.00 (2.11–2.00)	45.74–2.90 (3.06–2.90)	45.11–3.30 (3.50–3.30)		44.63–3.38 (3.56–3.38)	47.74–5.50 (5.80–5.50)
*R*_*merge*_	0.082 (1.854)	0.103 (1.733)	0.086 (1.497)	0.175 (1.108)		0.096 (0.763)	0.121 (1.359)
*I/σI*	20.09 (1.50)	12.3 (1.40)	12.4 (1.10)	11.5 (2.1)		8.8 (1.7)	8.1 (0.9)
CC (½)	1.0 (0.515)	1.0 (0.558)	1.0 (0.497)	1.0 (0.704)		1.0 (0.456)	0.99 (0.358)
Completeness (%)	99.8 (97.6)	99.9 (99.2)	99.9 (99.7)	99.3 (96.1)		99.4 (99.5)	97.6 (99.6)
Redundancy	16.09 (15.64)	10.3 (10.5)	6.9 (7.1)	12.6 (7.2)		3.4 (3.4)	3.3 (3.5)

**Refinement**							

Resolution (Å)	45.90–2.56	66.88–2.00	43.45–3.00	45.11–3.30			47.74–5.80
No. reflections (total)	29,681	39,658	15,305	27,912			6,655
*R*_*work*_/*R*_*free*_	0.23/0.26	0.19/0.22	0.22/0.24	0.23/0.28			0.29/0.30

**No. atoms**							

Protein	3,205	3,178	3,474	7,704			10,778
Ligand or ion	0	0	30	0			0
Water	41	163	0	0			0

**B-factors**							

Protein	88.71	59.86	124.61	121.21			210.41
Ligand or ion	NA	NA	172.37	NA			NA
Water	74.31	56.64	NA	NA			NA

**RMSDs**							

Bond lengths (Å)	0.003	0.007	0.004	0.064			0.004
Bond angles (°)	0.629	0.855	0.771	1.220			0.692

Values in parentheses are for the highest-resolution shell; ^∗^from two merged datasets (used for refinement). NA, not available; SAD, single-wavelength anomalous diffraction; SeMEt, selenomethionine; SIRAS, single isomorphous replacement with anomalous scattering.

A comparison of the nucleotide-free *Ct* Smc2_hd_ and Smc4_hd_–Brn1_C_ structures of condensin to the adenosine 5′-[γ-thio]triphosphate (ATPγS)-bound *Saccharomyces cerevisiae* (*Sc*) Smc1_hd_-Scc1_C_ and Smc3_hd_-Scc1_N_ structures of cohesin or to bacterial SMC ATPase head structures revealed differences in the orientations of the helical lobes and attached coiled coils relative to the RecA lobes, which can be explained by flexion movements of ∼25° or ∼15°, respectively (Figure 1B). These motions displace strictly conserved glutamine residues in the Smc2 and Smc4 Q loops, which are thought to coordinate the catalytic Mg^2+^ ion and contribute to nucleotide binding. Mutation of this residue in Smc4 to leucine indeed drastically reduced the low micromolar affinity of *Ct* Smc4_hd_-Brn1_C_ for ATP (Figure 1C). In contrast, even the wild-type (WT) version of *Ct* Smc2_hd_-Brn1_N_ was unable to bind ATP. This finding is readily explained by the *Ct* Smc2_hd_ structure, where the more pronounced flexion of the Smc2 helical lobe not only repositions the Q loop but also induces a cascade of structural displacement events that alter the P loop of the ATP-binding pocket into a conformation that is incompatible with nucleotide binding (Figure 1B). This incompatibility with ATP binding is even more obvious in a second crystal form of *Ct* Smc2_hd_ (Figure S1C).

### Distinct Contributions of the Two ATPase Sites to SMC Head Dimerization

Despite these structural differences, we were able to trap a stable heterodimer of *Ct* Smc2_hd_-Brn1_N_ and *Ct* Smc4_hd_-Brn1_C_ when we prevented ATP hydrolysis by mutation of the catalytic Walker B glutamate residues in both heads (*Ct* Smc2_hd E1116Q_, *Ct* Smc4_hd E1475Q_; Figures 1D and S2A). This is consistent with the absence of discernible steric clashes in a structural model of an ATP-dimerized *Ct* Smc2_hd_-Smc4_hd_-Brn1_C_ complex (Figure S2B). Consistent with the inability of *Ct* Smc2_hd_ to bind ATP (Figure 1C), preventing ATP hydrolysis only at the Smc2 active site was insufficient for dimer formation, whereas mutation of only the Smc4 active site was sufficient (Figures 1D and S2A). Mutation of the Smc2 signature motif serine residue that contacts the nucleotide bound at the Smc4 active site (*Ct* Smc2_hd S1088R_) prevented dimerization with Walker B mutant *Ct* Smc4_hd E1475Q_, whereas simultaneous mutation of the Smc4 signature motif (*Ct* Smc4_hd S1447R, E1475Q_) still allowed formation of a dimer that, however, eluted at a different retention volume during size-exclusion chromatography (Figure S2A). This suggests that a dimer with a distinct conformation can be mediated solely by ATP sandwiched between the Smc4 Walker A, Walker B, and Smc2 signature motifs. However, the second site formed by the Smc2 Walker A, Walker B, and Smc4 signature motifs must nevertheless be capable of binding and hydrolyzing ATP in the context of the heterodimer because mutation of the Smc2 Walker B motif had an even more severe effect on the basal ATPase activity of *Ct* Smc2_hd_-Brn1_N_ and *Ct* Smc4_hd_-Brn1_C_ complexes than mutation of the Smc4 Walker B motif (Figures S2C and S2D). ATP binding to the Smc4 active site is therefore sufficient to induce Smc2-Smc4 head dimerization, which then renders the Smc2 active site capable of binding and hydrolyzing ATP.

### A Conserved Patch on the Smc4 Head Binds to the Ycs4 HEAT-Repeat Subunit

**Figure 2:**
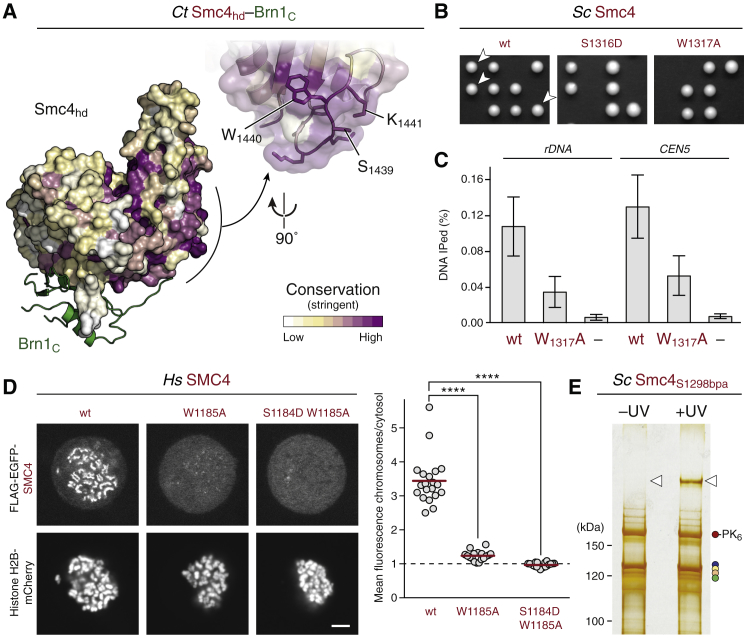
The Smc4 W-loop Is Essential for Condensin Function and Binds the Ycs4 Subunit (A) Surface conservation of the *Ct* Smc4_hd_-Brn1_C_ structure with a close-up view of the W-loop region. Residues chosen for mutational analysis are indicated. (B) Spores from diploid *Sc SMC4/smc4Δ* strains expressing an ectopic HA_6_-tagged copy of WT Smc4 (strain C4568), Smc4_S1316D_ (C4595), or Smc4_W1317A_ (C4570) W-loop mutant versions were dissected and incubated for 3 days at 30°C. (C) ChIP-qPCR at sites within the rDNA and at centromere V (*CEN5*) in diploid *Sc SMC4/smc4Δ* strains expressing no additional (C4936) or an ectopic HA_6_-tagged copy of WT Smc4 (C4568) or Smc4_W1317A_ (C4570) W-loop mutant versions (mean ± SD of 4 data points from 2 biological and 2 technical repeats each). (D) Snapshots of live HeLa cells expressing mCherry-tagged histone H2B (bottom) and ectopic copies of FLAG-EGFP-tagged WT SMC4, SMC4_W1185A_ single, or SMC4_S1184A, W1185A_ double mutant versions (top). The graph shows the ratio of mean fluorescence EGFP signals in the chromosomal to cytosolic area for 20 data points (circles) and median (horizontal line) from 2 independent experiments (^∗∗∗∗^p < 0.0001, Kolmogorov-Smirnov test). (E) Silver stain of immunoprecipitated *Sc* condensin complexes (C4681) expressing a bpa-modified version as the only copy of Smc4 without (–UV) or after (+UV) crosslinking. See also Figure S3.

A striking feature of the Smc4_hd_ helical lobe is a highly conserved surface patch formed by residues within a loop that surround a strictly conserved tryptophan residue (W-loop; Figure 2A). The corresponding region in the homologous Smc1_hd_ of cohesin also displays some degree of conservation, whereas the regions in the Smc2_hd_ or Smc3_hd_ structures show no obvious sequence conservation (Figure S3A; Table S2). Mutation to alanine of the strictly conserved tryptophan residue of Smc4 rendered budding yeast cells non-viable (*Sc* Smc4_W1317A_), as did mutation to aspartate of the neighboring serine residue (*Sc* Smc4_S1316D_; Figure 2B; Table S3). Mutation of the latter to alanine (*Sc* Smc4_S1316A_), of the arginine residue following the tryptophan residue to aspartate (*Sc* Smc4_R1318E_), or of the corresponding tryptophan or lysine residues in *Sc* Smc2 (*Sc* Smc2_W1077A_, *Sc* Smc2_K1078E_) had a less dramatic effect on cell proliferation (Figure S3B). None of these mutations affected Smc2 or Smc4 expression levels (Figure S3B). The loss of viability upon mutation of the Smc4 but not upon mutation of the Smc2 W-loop tryptophan residue can be explained by their differential effect on ATP hydrolysis rates because only the former (*Ct* Smc4_hd W1440A_) but not the latter (*Ct* Smc2_hd W1080A_) dramatically reduced ATP turnover by *Ct* Smc2_hd_-Brn1_N_ and *Ct* Smc4_hd_-Brn1_C_ complexes (Figures S2D and S2E). Because the heterodimer formed between *Ct* Smc2_hd_-Brn1_N_ and W-loop mutant Smc4_hd_-Brn1_C_ eluted during size-exclusion chromatography at the same retention volume as the dimer formed between *Ct* Smc2_hd_-Brn1_N_ and signature motif mutant Smc4_hd_-Brn1_C_ (Figure S2A), it is likely that the Smc4 W-loop mutation prevents the neighboring signature motif from sandwiching ATP bound to the Smc2 active site, which explains the reduction in ATPase rates. As an expected consequence of their inability to complete a full ATPase cycle, chromosome binding of yeast (Figure 2C) and human (Figure 2D) condensin complexes with Smc4 W-loop mutations was dramatically reduced when measured by chromatin immunoprecipitation followed by qPCR (ChIP-qPCR) or live-cell microscopy, respectively. We conclude that the conserved W-loop of Smc4 is essential for condensin’s ATPase activity and for the stable association of condensin complexes with chromosomes.

To identify proteins that potentially interact with the W-loop surface patch of Smc4, we introduced the non-natural amino acid *p*-benzoyl-L-phenylalanine (bpa) into one of several positions surrounding the *Sc* Smc4 W-loop for *in vivo* photo-crosslinking (Chen et al., 2007). Three of seven different *Sc* Smc4_bpa_ constructs produced upshifted bands in immunoblots for the PK_6_ epitope tag fused to the C terminus of *Sc* Smc4 after UV crosslinking in live yeast cells (Figure S3C). Mass spectrometry of the upshifted band identified, in addition to *Sc* Smc4 itself, peptides of the *Sc* Ycs4 HEAT-repeat subunit and, to a lesser extent, the *Sc* Brn1 kleisin subunit (Figure 2E; Table S4). Western blotting against HA_6_ epitope tags fused to the C termini of *Sc* Ycs4 or *Sc* Brn1 confirmed the presence of these subunits in the upshifted bands, whereas the Ycg1 HEAT-repeat subunit did not shift under these conditions (Figure S3D).

### Ycs4 Forms a Complex with Brn1 via Two Independent Binding Interfaces

**Figure 3:**
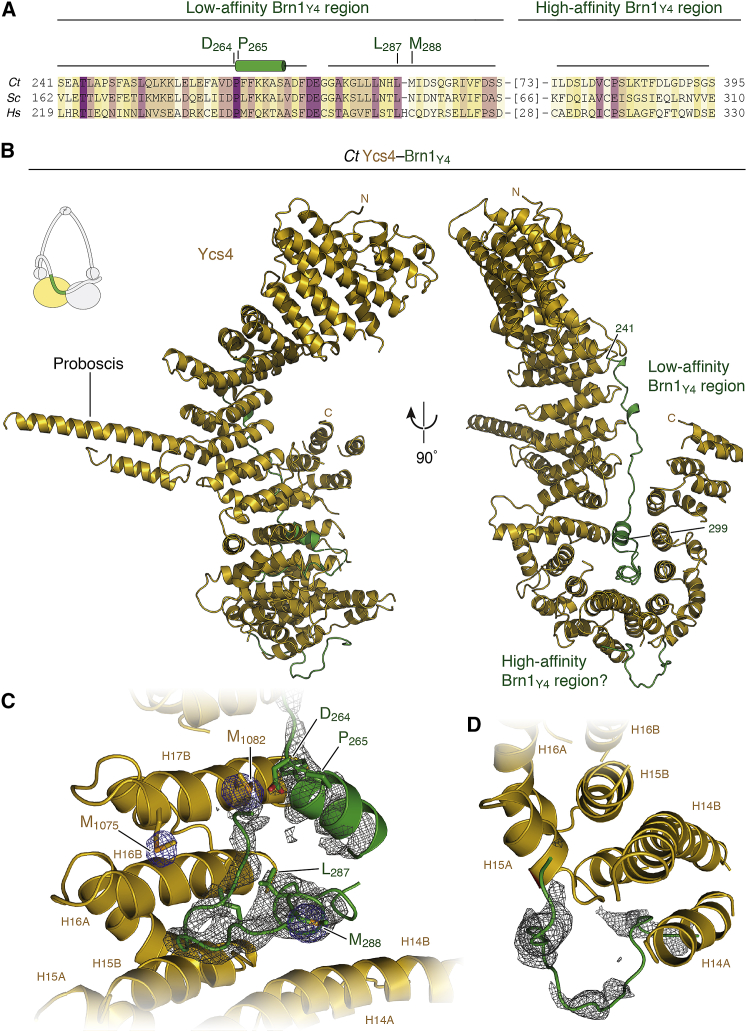
Structure of the Ycs4-Brn1_Y4_ Complex (A) Partial alignment of the extended Brn1_Y4_ region that binds Ycs4. Colors indicate conservation scores calculated from an alignment of sequences from 40 species. Residues and secondary elements highlighted in the *Ct* Ycs4–Brn1_Y4_ structure are indicated. (B) Cartoon model of the 21 HEAT-repeat motifs of *Ct* Ycs4 (yellow) bound to residues 241 to 299 of *Ct* Brn1_Y4_ (green). A helical insertion between Ycs4 HEAT-repeat motifs 8 and 9 creates an extended “trunk” (proboscis). (C) Close-up view of Brn1_Y4_ electron density in the Ycs4 U-turn. Anomalous difference density marking selenomethionine positions is shown in blue. (D) Close-up view of additional electron density at the distal tip of the Ycs4 U-turn that presumably matches residues of the Brn1_Y4_ high-affinity interaction region included in the crystallization construct. See also Figures S4 and S5.

Because Ycs4 has so far been thought to assemble into the condensin complex exclusively through its constitutive interaction with the Brn1 kleisin subunit (Onn et al., 2007, Piazza et al., 2014), we mapped the part of *Ct* Brn1 required for *Ct* Ycs4 binding to a high-affinity (K_d_ = 0.70 nM) core region of ∼175 residues (*Ct* Brn1_336–512_) and to an extended region that includes the preceding ∼110 residues (*Ct* Brn1_225–512_; Figures S4A and S4B). The additional segment of the extended region displays a higher degree of sequence conservation (Figures 3A and S4C) but lower affinity for *Ct* Ycs4 binding (Figure S4). Further truncation experiments showed that the N-terminal ∼690 residues of *Ct* Ycs4 are sufficient for binding to the extended *Ct* Brn1 segment (Figure S4D) but with strongly reduced affinity (K_d_ = 1.22 μM) when compared to full-length *Ct* Ycs4 (Figure S4B). These results point to the presence of two distinct sites of contact between *Ct* Ycs4 and *Ct* Brn1: a high-affinity interface between the conserved C-terminal region of Ycs4 and the less conserved kleisin core binding region (*Ct* Brn1_336–512_), and a low-affinity interface between the less conserved N-terminal part of Ycs4 and the more highly conserved extension of the kleisin core region (*Ct* Brn1_225–335_).

### Structure of the *Ct* Ycs4-Brn1 Complex

To reveal the interaction between the Brn1 kleisin and Ycs4 HEAT-repeat subunits at near-atomic resolution, we solved the co-crystal structure of *Ct* Ycs4 bound to *Ct* Brn1_225–418_ to 3.3-Å resolution (*Ct* Ycs4-Brn1_Y4_; Figure 3B; Table 1). The structure revealed a hook-shaped conformation of the 21 HEAT-repeat motifs of Ycs4 (Figure S5A), with the low-affinity binding region of the kleisin subunit (*Ct* Brn1_241–299_) winding along the concave surface of the HEAT-repeat solenoid (Figure 3B) and entirely filling the space between the two lobes of the sharp U-turn in the C-terminal part of the Ycs4 subunit (Figure 3C). A marked drop in the quality of the electron density map in the second lobe because of crystallographic disorder only allowed modeling of a polyalanine chain into unaccounted electron density alongside the tip of the Ycs4 U-turn, which presumably corresponds to the high-affinity kleisin core binding region (Figure 3D). Localization of selenomethionine residues based on anomalous difference maps and secondary structure prediction nevertheless allowed the assignment of residue numbers for almost all of Ycs4.

A search for similar structures in the PDB using the DALI server (Holm and Laakso, 2016) returned several HEAT-repeat proteins that fold into similarly curved shapes, including the cohesin subunits Pds5 and Scc3^SA2^ (Hara et al., 2014, Ouyang et al., 2016) and the condensin subunit Ycg1 (Kschonsak et al., 2017; Figure S5B). Comparison with the structure of the Scc2 subunit of the cohesin loader complex (Kikuchi et al., 2016) also revealed a strikingly similar overall shape despite the fact that this protein was not included in the list from the DALI server. A feature apparently unique to Ycs4 is an α-helical extension of HEAT-repeat motif 10 (“proboscis”; Figure 3B), which showed, however, no apparent primary sequence conservation among Ycs4 homologs (Figure S5A).

### Structure of the *Ct* Ycs4-Brn1_Y4_-Smc4_hd_-Brn1_C_ Complex

**Figure 4:**
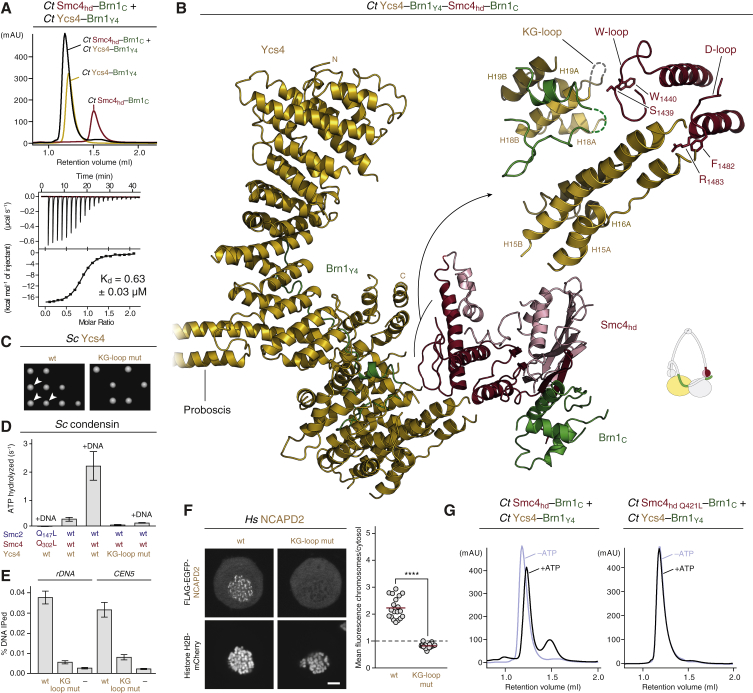
Structure and ATP-Dependent Release of the Ycs4-Smc4_hd_ Interaction (A) Size-exclusion chromatography of complexes formed between WT *Ct* Ycs4-Brn1_Y4_ and *Ct* Smc4_hd_-Brn1_C_ and ITC of *Ct* Ycs4-Brn1_Y4_ binding to *Ct* Smc4_hd_-Brn1_C_ (fit ± error of the fit). (B) Cartoon model of the co-crystal structure of *Ct* Ycs4-Brn1_Y4_ and *Ct* Smc4_hd_-Brn1_C_. A close-up view highlights contacts between the Smc4 W- and D-loops (red) with the Ycs4 KG-loop (yellow dotted line) and the helical extension of Ycs4 HEAT-repeat motif 15, respectively. (C) Spores from diploid *Sc YCS4/ycs4Δ* strains expressing an ectopic PK_6_-tagged copy of WT (strain C5005) or KVKGQL-DSDGDS KG-loop mutant (C5007) versions of Ycs4 were dissected and incubated for 3 days at 30°C. (D) ATPase assays with WT (purified from strain C4491), Smc2 and Smc4 Q-loop (C4724), or Ycs4 KG-loop mutant (C5050) *Sc* condensin holocomplexes in the absence or presence of DNA (mean ± SD of 3 independent experiments). (E) ChIP-qPCR at the rDNA and centromere V (*CEN5*) in diploid *Sc YCS4/ycs4Δ* strains expressing no (C5003) or an ectopic PK_6_-tagged copy of WT (C5005) or KG-loop mutant (C5007) Ycs4 (mean ± SD of 4 data points from 2 biological and 2 technical repeats each). (F) Snapshots of HeLa cells expressing mCherry-tagged histone H2B (bottom) and ectopic copies of FLAG-EGFP-tagged WT or KVKGQV-DSDGDS KG-loop mutant NCAPD2 (top). The graph shows the ratio of mean fluorescence EGFP signals in the chromosomal to cytosolic area for 20 data points (circles) and median (horizontal line) from 2 independent experiments (^∗∗∗∗^p < 0.0001, Kolmogorov-Smirnov test). (G) Size-exclusion chromatography of complexes formed between *Ct* Ycs4-Brn1_Y4_ and WT (left) or Q-loop mutant (right) versions of *Ct* Smc4_hd_-Brn1_C_ in the absence (–ATP, light blue) or presence (+ATP, black) of ATP. See also Figures S5 and S6.

Consistent with the finding that the *Sc* Smc4 W-loop cross-links to *Sc* Ycs4 and *Sc* Brn1 *in vivo* (Figure 2E), *Ct* Ycs4-Brn1_Y4_ and *Ct* Smc4_hd_-Brn1_C_ formed a stable stoichiometric complex *in vitro* (K_d_ = 0.63 μM; Figures 4A and S6A). We solved the co-crystal structure of this complex to 5.8-Å resolution (Table 1) and used the high-resolution structures of the individual subcomplexes (Figures 1A and 3B) and deformable elastic network refinement to build a coarse model of the *Ct* Ycs4-Brn1_Y4_-Smc4_hd_-Brn1_C_ complex (Figure 4B). As expected, the Smc4 W-loop mediates the majority of interactions with the HEAT-repeat subunit and does so by contacting the second lobe of the Ycs4 U-turn. In addition, a strictly conserved phenylalanine-arginine residue pair adjacent to the so-called D-loop of the *Ct* Smc4 ATPase head contacts a helical extension of Ycs4 HEAT repeat 15. Despite the low resolution in the C-terminal half of Ycs4, we identified the highly conserved peptide loop that connects HEAT repeats 18 and 19 and contains a conserved lysine-glycine pair (KG-loop; Figure S5A) as the most likely candidate to mediate the interaction with the Smc4 W-loop by correlating sequence and structure based on the anomalous scattering of the selenomethionine residues and the prediction of secondary structure and HEAT-repeat organization (Figure 4B).

Mutation of the relevant residues in the Smc4 W- or D-loops or in the Ycs4 KG-loop sequence disrupted complex formation between *Ct* Ycs4-Brn1_Y4_ and *Ct* Smc4_hd_-Brn1_C_ (*Ct* Smc4_hd SW-DA_, *Ct* Smc4_hd FR-AE_, or *Ct* Ycs4_KG loop mut_, respectively; Figure S6A). Similar to mutation of the Smc4 W-loop, replacement of the Ycs4 KG-loop sequence rendered condensin non-functional in budding yeast (*Sc* Ycs4_KG-loop mut_; Figure 4C) without affecting expression levels (Figure S6B). It furthermore severely compromised the basal and DNA-stimulated ATPase activities of condensin holocomplexes (Figures 4D and S6C) and strongly reduced condensin binding to chromosomes in budding yeast (Figure 4E) or mitotic human cells (*Homo sapiens* NCAPD2_KG-loop mut_; Figure 4F). Like the Ycs4 KG-loop, the high-affinity core binding region of Brn1_Y4_ is not visible in the structure because of crystallographic disorder. It nevertheless must contribute to the Smc4 interaction because its presence was essential for complex formation of *Ct* Ycs4-Brn1_Y4_ with *Ct* Smc4_hd_-Brn1_C_ in pull-down assays (Figure S6D). We conclude that a contact between the Smc4 head and the Ycs4 HEAT-repeat subunit is mediated via contacts of Smc4 W- and D-loop residues with the Ycs4 KG-loop and the core region of Brn1_Y4_. This contact is essential for condensin's abilities to complete a full ATPase cycle and to associate with chromosomes.

### ATP-Dependent Control of the Ycs4-Smc4 Interaction

When we superimposed the model of the engaged *Ct* Smc2_hd_-Smc4_hd_-Brn1_C_ ATPase head dimer (Figure S2B) onto the *Ct* Ycs4-Brn1_Y4_-Smc4_hd_-Brn1_C_ structure, we noticed a pronounced steric clash between Smc2 and Ycs4 (Figure S6E). This implies that ATP-dependent Smc2-Smc4 head dimerization is incompatible with simultaneous binding of Ycs4-Brn1_Y4_ to the Smc4 head. Consistent with this prediction, we found that addition of *Ct* Smc2_hd_-Brn1_N_ and ATP disrupted the interaction between *Ct* Ycs4-Brn1_Y4_ and *Ct* Smc4_hd_-Brn1_C_ in pull-down assays (Figure S6F). Surprisingly, addition of ATP alone was similarly sufficient to compete with complex formation in this assay (Figure S6F) and during size-exclusion chromatography (Figures 4G and S6G). This effect was not caused by Smc4_hd_ homodimerization in the presence of ATP, which has been observed for the homologous Smc1_hd_ of cohesin (Haering et al., 2004), because ATP addition similarly disrupted Ycs4-Brn1_Y4_ binding to a version of Smc4_hd_-Brn1_C_ that was unable to dimerize because of a mutation in the signature motif (*Ct* Smc4_hd S1447R_; Figure S6G).

Addition of ADP, but not of AMP or guanosine triphosphate (GTP), prevented *Ct* Ycs4-Brn1_Y4_ binding to *Ct* Smc4_hd_-Brn1_C_ with a similar efficiency as addition of ATP (Figure S6H). If ATP or ADP binding to the Smc4 RecA lobe induced dissociation of Ycs4-Brn1_Y4_ from the helical lobe via a flexion movement of the two lobes (Figure 1B), then mutation of the central Q-loop should render the Ycs4-Brn1_Y4_-Smc4_hd_-Brn1_C_ complex insensitive to nucleotide addition. This was indeed the case (*Ct* Smc4_hd Q421L_; Figures 4G and S6G). To rule out that the continued association of the Smc4 Q-loop mutant complex in the presence of ATP was not merely due to the reduced affinity for nucleotide binding that results from a failure to coordinate Mg^2+^ at the active site (Figure 1C), we repeated the experiment under conditions (10 mM MgCl_2_) that still allowed ATP binding by the Smc4_hd_ Q-loop mutant with micromolar affinity (K_d_ = 18.35 μM; Figure S6I). Even under these conditions, ATP addition (1 mM) failed to release *Ct* Ycs4-Brn1_Y4_ from *Ct* Smc4_hd_-Brn1_C_ (Figure S6G). These results strongly support a central role of the Q-loop motif in a conformational switch that transmits an allosteric change from the nucleotide-binding pocket of Smc4_hd_ to its Ycs4-Brn1_Y4_ W-loop interface.

### The Smc2 Neck Region Binds Brn1

**Figure 5:**
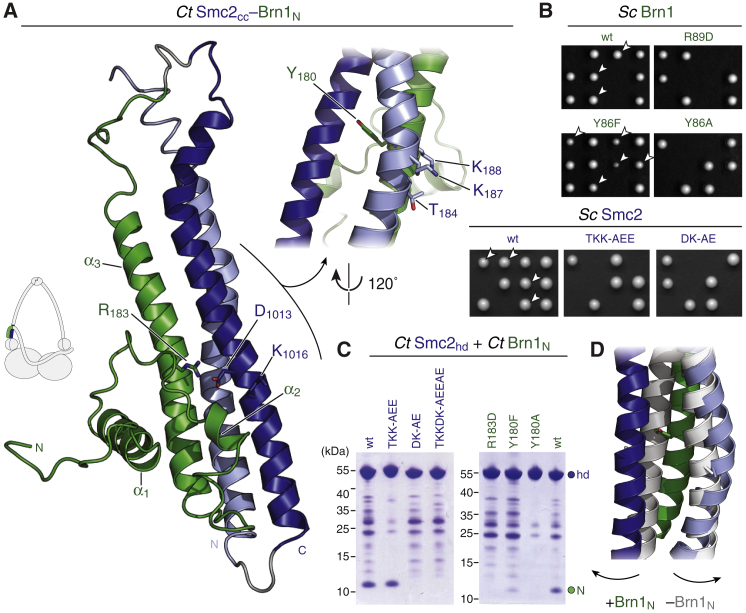
Structure of the Smc2_cc_-Brn1_N_ Interface (A) Cartoon model of the lowest-energy *Ct* Smc2_cc_-Brn1_N_ NMR structure. Residues chosen for mutational analysis are highlighted. The close-up view shows *Ct* Brn1_Y180_ intercalating into the Smc2 coiled coils and the positions of additional residues chosen for mutagenesis. (B) Diploid *Sc BRN1/brn1Δ* strains expressing an ectopic PK_6_-tagged copy of WT Brn1 (strain C5239) or Brn1_R89D_ (C5263), Brn1_Y86F_ (C5262) or Brn1_Y86A_ (C5261) mutant versions and diploid *Sc SMC2/smc2Δ* strains expressing an ectopic PK_6_-tagged copy of WT Smc2 (C5277), Smc2_TKK-AEE_ (C5278), or Smc2_DK-AE_ (C5279) mutant versions were dissected and incubated for 3 days at 30°C. (C) Co-elution of His_6_-tagged WT or mutant *Ct* Smc2_hd_ constructs (left) and untagged WT or mutant *Ct* Brn1_N_ constructs (right) from Ni-Sepharose beads tested by SDS-PAGE and Coomassie staining of elution fractions. (D) Comparison of Smc2_cc_ conformations in the *Ct* Smc2_cc_-Brn1_N_ NMR and *Ct* Smc2_hd_ crystal structures. C_β_ atoms of Smc2_A186_ and Smc2_H1015_, shown as sticks, serve as rotational markers. See also Figure S7.

Because the Smc2_hd_-Brn1_N_ complex was refractory to crystallization, we determined an NMR structure of this dynamic interface by fusing the *Ct* Brn1_N_ domain to the coiled-coil neck region of *Ct* Smc2 (Figures 5A and S7A; Table S5). Similar to the Smc3_hd_-Scc1_N_ interface in cohesin (Gligoris et al., 2014), the third “contact” helix (α_3_) of the kleisin subunit forms an ∼50-Å-long helical bundle with the SMC neck coiled coil (Figure S7B). This contact is stabilized by a salt bridge between highly conserved arginine and aspartate residues in the Brn1 contact helix and the C-terminal Smc2 neck helix, respectively (*Ct* Brn1_R183_, *Ct* Smc2_D1013_; Figures 5A and S7C). Mutations of these and neighboring residues rendered condensin nonfunctional in budding yeast (*Sc* Brn1_R89D_ and *Ct* Smc2_DK-AE_; Figures 5B and S7D) and disrupted the *Ct* Smc2_cc_-Brn1_N_ interaction (Figure 5C). Mutation of conserved residues in the N-terminal Smc2 neck helix that make no direct contact with Brn1 had, in contrast, no effect on the *Ct* Smc2_cc_-Brn1_N_ interaction (*Ct* Smc2_TKK-AEE_; Figure 5C) while nevertheless rendering condensin non-functional in yeast (Figure 5B). This suggests that the role of the Smc2 neck region might go beyond providing a binding platform for the kleisin subunit.

Comparison of the coiled-coil conformations in the *Ct* Smc2_hd_ and *Ct* Smc2_cc_-Brn1_N_ structures implies that the formation of the three-helix bundle requires rotation of one helix by 60°–90° relative to the other helix and spreading of the two helices by 2.4 Å (Figure 5D). The deformation of the coiled coil is caused by the insertion of a conserved tyrosine residue located within the Brn1 contact helix (*Ct* Brn1_Y180_; Figure 5A). A similar insertion of a conserved kleisin tyrosine residue between the coiled-coil helices can also be observed in the *Sc* Smc3_hd_-Scc1_N_ crystal structure of cohesin (*Sc* Scc1_Y83_; Figure S7B), which suggests that this binding mode might be generally conserved among kleisin-SMC protein complexes. Mutation of this tyrosine residue to alanine eliminated binding to Smc2 *in vitro* (*Ct* Brn1_Y180A_; Figure 5C) and condensin function *in vivo* (*Sc* Brn1_Y86A_; Figures 5B and S7D), whereas mutation to a chemically similar phenylalanine residue had a more gradual effect on the Smc2_cc_-Brn1_N_ interaction (*Ct* Brn1_Y180F_; Figure 5C) and cell proliferation (*Sc* Brn1_Y86F_; Figures 5B and S7D).

### ATP-Dependent Release of the Brn1 Kleisin from the Smc2 Coiled Coil

**Figure 6:**
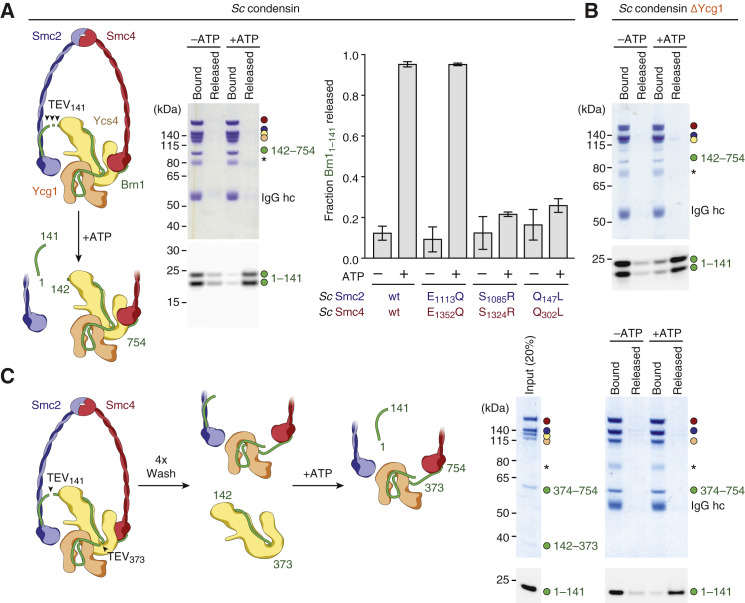
ATP-Binding-Dependent Release of the Smc2_cc_-Brn1_N_ Interaction (A) *Sc* condensin holocomplexes containing a Brn1 subunit labeled via an N-terminal ybbR tag with the fluorescent dye ATTO488 and a triple TEV protease cleavage site at position 141 were incubated with TEV protease before immunoprecipitation. Shown is Coomassie staining (top) and an in-gel fluorescence scan (bottom) of SDS-PAGE of bound or released fractions after washing immunoprecipitation beads with buffer without nucleotide (–ATP) or containing 1 mM ATP (+ATP). The graph shows fluorescence intensities of the Brn1_1–141_ fragment in the released fractions of WT (purified from strain C5066), Walker B (Smc2_E1113Q_, Smc4_E1352Q_; C5142), signature motif (Smc2_S1085R_, Smc4_S1324R_; C5139), or Q-loop (Smc2_Q147L_, Smc4_Q302L_; C5125) mutant condensin complexes (mean ± SD from 3 independent experiments). (B) As in (A), using purified condensin complexes that lack the Ycg1 subunit (C5110). (C) Brn1 of condensin holocomplexes (C5122) was cleaved simultaneously at positions 141 and 373. Extensive washing steps during immunoprecipitation removed Ycs4-Brn1_142–373_. Immunoprecipitated condensin complexes were then washed with buffer without nucleotide (–ATP) or containing 1 mM ATP (+ATP) as in (A). See also Figure S7.

Spreading of the Smc2 coiled-coil helices to allow insertion of a tyrosine residue of the Brn1 contact helix provides a potential mechanism for allosteric control of this crucial interface in the condensin ring. We therefore purified *Sc* condensin holocomplexes in which we had inserted a triple repeat of the tobacco etch virus (TEV) protease cleavage site succeeding the Brn1 N-terminal domain (*Sc* Brn1_1–141_) and followed the fate of this domain after TEV cleavage. In the absence of nucleotide, the majority of Brn1_N_ remained bound to immobilized condensin (Figure 6A) or co-eluted with the complex during size-exclusion chromatography (Figure S7E). ATP addition released most (∼95%) of the Brn1_N_ cleavage fragments from condensin, even when nucleotide hydrolysis was prevented by Walker B mutations (*Sc* Smc2_E1113Q_, *Sc* Smc4_E1352Q_; Figures 6A and S7E). In contrast, mutations in the Q-loop motifs or in the signature motifs that prevent head dimerization rendered the Smc2-Brn1_N_ interaction insensitive to ATP addition (*Sc* Smc2_Q147L_, *Sc* Smc4 _Q302L_ or *Sc* Smc2_S1085R_, *Sc* Smc4_S1323R_, respectively; Figure 6A). ATP-dependent Brn1_N_ dissociation was not affected by the absence of the Ycg1 HEAT-repeat subunit (Figure 6B) or release of the Ycs4-Brn1_Y4_ subcomplex prior to nucleotide addition (Figure 6C). We conclude that ATP-dependent Smc2-Smc4 head dimerization following nucleotide binding induces condensin ring opening at the Smc2-Brn1 interface independent of the presence of either HEAT-repeat subunit.

## Discussion

**Figure 7:**
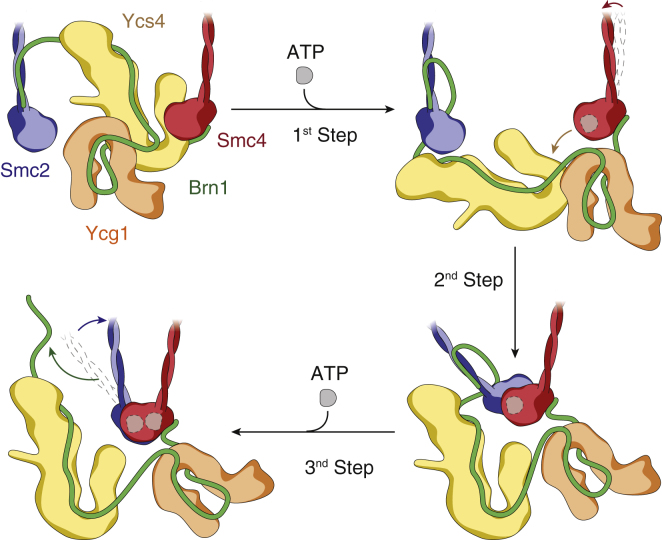
Model of the Condensin ATP-binding Cycle Binding of ATP to the Smc4_hd_ P loop site induces flexion of RecA and helical lobes that re-orient the coiled coils and W-loop regions and release the interaction with the Ycs4 subunit (step 1). Dissociation of Ycs4 allows Smc4_hd_ engagement with Smc2_hd_ via the bound ATP into an asymmetric dimer (step 2). This induces major flexion between the Smc2_hd_ RecA and helical lobes that render the Smc2_hd_ P loop capable of binding the second ATP molecule, resulting in a pseudo-symmetric Smc2_hd_-Smc4_hd_ dimer that sandwiches two ATP molecules between its catalytic sites (step 3). The simultaneous conformational change in the Smc2_hd_ coiled coil releases Brn1_N_ to open the condensin ring.

Based on the current work, we propose a multi-step model of the condensin ATPase cycle (Figure 7). In the nucleotide-free state, only the Smc4 head is able to bind an ATP molecule (Figure 1C). Correlation of the nucleotide-free Smc4_hd_-Brn1_C_ structure with the ATPγS-bound structure of the homologous Smc1_hd_-Scc1_C_ complex (Haering et al., 2004) suggests that, upon ATP binding to the P loop of the Smc4 RecA lobe, the Q-loop glutamine residue repositions to form hydrogen bonds with the Mg^2+^ ion and the γ-phosphate (Figure 1B). As a consequence, the helical lobe tilts, relative to the RecA lobe, by 15° degrees (Video S1), which is similar to the flexion recently described for bacterial SMC proteins (Kamada et al., 2017). The comparison of the Smc4_hd_-Brn1_C_ and Smc1_hd_-Scc1_C_ structures furthermore reveals an inversion of the side-chain conformations of a conserved tyrosine-(lysine or arginine) pair in a region of the helical lobe that we define as the W-loop (Figure S7F). It is likely that tilting and repositioning of W-loop residues dissociate Ycs4 (Figure 4G), which binds to this part of Smc4_hd_ (Figure 4B) and sterically blocks access of Smc2_hd_ (Figure S6E).

Video S1. Model of the Conformational Changes Induced by ATP Binding to Smc4_hd_, Related to Figure 7 ATP binding to the P-loop of Smc4_hd_-Brn1_C_ induces a Q-loop-mediated flexion between RecA and helical lobes to disengage Ycs4-Brn1_Y4_ (step 1).

Ycs4, whose role for condensin function has so far remained incompletely understood, binds to two distinct sequence stretches of the Brn1 kleisin subunit (Figure S4): the N-terminal, low-affinity binding stretch winds along the inner surface of the hook-shaped HEAT-repeat solenoid (Figure 3C), whereas the high-affinity core binding region stretch is not clearly resolved in the crystal structure, although it is essential for the interaction of the Ycs4-Brn1_Y4_ subcomplex with Smc4_hd_ (Figure S6D). It is conceivable that contacts with this part of the kleisin subunit support the essential contacts between the Ycs4 KG-loop and the Smc4 W-loop and between the helical extension of Ycs4 HEAT-repeat motif 15 and the conserved phenylalanine-arginine residue pair at the tip of the Smc4 D-loop. Notably, the conservation of the KG-loop sequence extends to the NCAPD3 HEAT-repeat subunits of metazoan condensin II complexes (Figure S5A) and the identity of several residues in the loop segment of Smc1_hd_ that corresponds to the Smc4_hd_ W-loop has been maintained throughout evolution (Figure S3A). These findings raise the possibility that the interaction of a HEAT-repeat subunit with the κ-SMC_hd_ is a central feature of all condensin and cohesin complexes. In cohesin, the most likely HEAT-repeat subunit to bind Smc1_hd_ might be Pds5, which is most similar in structure to Ycs4 (Figure S5B).

Smc2-Smc4 head dimerization commences in an asymmetric state in which only the Smc4 ATP-binding pocket is occupied (Figure 7). The Smc2 head fails to bind ATP on its own (Figure 1C), presumably because of the deformation of the Smc2 P loop observed in two different crystal forms (Figures 1B and S1C), but readily binds and hydrolyses ATP when in complex with the Smc4 head (Figure S2C). This transition is presumably the consequence of a pronounced reorientation of the Smc2 helical lobe and coiled coil by ∼25° upon head dimerization, which is transmitted via D-loop and adjacent helices to create a P loop conformation that is compatible with nucleotide binding (Figure 1B). The subsequent sandwiching of the second ATP by the Smc4 signature motif presumably also depends on the prior reorientation of the neighboring W-loop because mutation of either of the two motifs results in head dimers with distinct elution profiles during size-exclusion chromatography (Figure S2A).

Our data furthermore suggest that, during formation of a pseudo-symmetric Smc2-Smc4 head dimer with both active sites occupied by ATP, changes in the Smc2 coiled-coil conformation markedly reduce the binding affinity to the Brn1 N terminus (Figure 6A). Because we observe Brn1 dissociation in the context of the full-length Smc2-Smc4 dimers but not for the Smc2_hd_-Brn1_N_ subcomplex (Figure S1A), the conformational transition of the coiled coil might require a pivot point, which could be situated in the “joint” region approximately one-third up the length of the coiled coil (Diebold-Durand et al., 2017) or generated by folding back of the SMC hinge domain onto the coiled coils (Bürmann et al., 2019). In either case, helix rotation provides a straightforward mechanism to control the propensity of the coiled coils to spread apart and accommodate insertion of the kleisin tyrosine side chain (Figure 5A). The fact that the cohesin Scc1 kleisin subunit binds Smc3 in a similar fashion (Gligoris et al., 2014) and is also released upon ATP binding (Beckouët et al., 2016, Murayama and Uhlmann, 2015) strongly suggests that the reaction cycle we describe for condensin is fundamental to the action of all SMC protein complexes.

It seems reasonable to assume that the ATP-dependent conformational changes we describe provide the mechanistic basis for condensin to translocate along the DNA double helix and thereby extrude large DNA loops (Ganji et al., 2018, Terakawa et al., 2017), most likely by driving alternating DNA association and dissociation events. For example, binding of Ycs4 to the Smc4 head creates a topological compartment that might encircle DNA in a similar fashion as the Ycg1-Brn1 safety-belt compartment (Kschonsak et al., 2017). Because ADP, like ATP, is sufficient to dissociate Ycs4 from Smc4 (Figure S6H), it seems reasonable that such a compartment would only exist in a temporary nucleotide-free state. The structural transitions we report for the Smc2-Smc4 ATPase cycle would hence be reset not by hydrolysis, but only after nucleotide release, which is similar to many other ABC-type ATPases (Hopfner, 2016). Re-binding of Ycs4 to Smc4 at this point in the ATPase cycle might be an integral element because mutations that disrupt Ycs4 binding to Smc4 also affect ATPase rates (Figures 4D and S2E). The directionality that derives as a consequence of the strict asymmetry of this reaction cycle could resolve the conundrum how condensin is able to processively track along symmetric DNA molecules in one direction over distances of several kilobase pairs.

It is important to note that the asymmetry of condensin differs from that described for certain heterodimeric ABC transporters, which operate in an asymmetric fashion because only one of the two ATPase sites seems to be capable of hydrolyzing ATP (Procko et al., 2009). Furthermore, it has been suggested that many homodimeric ABC transporters use their two catalytic sites in an alternating fashion (Jones and George, 2013). Because even the two ATPase heads of bacterial SMC homodimers are embedded asymmetrically into the holocomplex by their binding to different ends of the kleisin subunit (Bürmann et al., 2013, Zawadzka et al., 2018), the asymmetric model put forward by our analysis of condensin might not only explain functional differences between the two ATPase sites of condensin’s Smc2-Smc4 subunits or cohesin’s Smc1-Smc3 subunits (Elbatsh et al., 2016) but, conceivably, apply to all SMC protein complexes. Future studies will need to complete the structural landscape of SMC holocomplexes to uncover how the asymmetric ATPase motor drives the diverse functions of this class of chromosome organizers.

## STAR★Methods

### Key Resources Table

**Table:** 

REAGENT OR RESOURCE	SOURCE	IDENTIFIER
**Antibodies**		

Mouse monoclonal anti V5-tag (anti PK6-tag)	AbD Serotec	Cat# MCA1360, RRID: AB_322378
Rabbit polyclonal anti HA-tag	Abcam	Cat# ab9110, RRID: AB_307019
Mouse monoclonal anti tubulin (TAT1)	Woods et al., 1989	N/A
Mouse monoclonal anti HA-tag (12CA5)	EMBL Protein Expression and Purification Core Facility	N/A
Rabbit polyclonal anti Sc Ycg1	Piazza et al., 2014	N/A

**Bacterial and Virus Strains**		

*Escherichia coli* Rosetta (DE3) pLysS	Merck	Cat# 70954

**Chemicals, Peptides, and Recombinant Proteins**		

*C. thermophilum* 6 × HIS–Brn1_112–204_ in complex with Smc2_2–224/981–1179_	This work	N/A
*C. thermophilum* 6 × HIS–Brn1_112–204_ in complex with Smc2_2-224/981–1179, E1116Q_	This work	N/A
*C. thermophilum* 6 × HIS–Brn1_112–204_ in complex with Smc2_2–224/981–1179, Q147L_	This work	N/A
*C. thermophilum* 6 × HIS–Brn1_112–204_ in complex with Smc2_2–224/981–1179, S1088R_	This work	N/A
*C. thermophilum* 6 × HIS–Brn1_112–204_ in complex with Smc2_2–224/981–1179, W1080A_	This work	N/A
*C. thermophilum* 6 × HIS–Brn1_112-204_ in complex with Smc2_2–215/990–1179_	This work	N/A
*C. thermophilum* Brn1_112–204_ in complex with Smc2_2–224/981–1179_–6 × HIS	This work	N/A
*C. thermophilum* Brn1_112–204_ in complex with Smc2_2–224/981–1179, T184A, K187E, K188E_–6 × HIS	This work	N/A
*C. thermophilum* Brn1_112-204_ in complex with Smc2_2–224/981–1179, D1013A, K1016E_–6 × HIS	This work	N/A
*C. thermophilum* Brn1_112-204, R183D_ in complex with Smc2_2–224/981–1179_–6 × HIS	This work	N/A
*C. thermophilum* Brn1_112-204, Y180A_ in complex with Smc2_2–224/981–1179_–6 × HIS	This work	N/A
*C. thermophilum* Brn1_112–204, Y180I_ in complex with Smc2_2-224/981–1179_–6 × HIS	This work	N/A
*C. thermophilum* Brn1_112-204, Y180F_ in complex with Smc2_2–224/981–1179_–6 × HIS	This work	N/A
*C. thermophilum* 6 × HIS–Brn1_112–204_ fused to Smc2_981–1031/170–224_	This work	N/A
*C. thermophilum* Brn1_765–898_ in complex with Smc4_264–466/1367–1542_–8 × HIS	This work	N/A
*C. thermophilum* Brn1_765–898_ in complex with Smc4_264–466/1367–1542, E1475Q_–8 × HIS	This work	N/A
*C. thermophilum* Brn1_765–898_ in complex with Smc4_264–466/1367–1542, Q421L_–8 × HIS	This work	N/A
*C. thermophilum* Brn1_765–898_ in complex with Smc4_264–466/1367–1542, E1475Q, S1447R_– 8 × HIS	This work	N/A
*C. thermophilum* Brn1_765–898_ in complex with Smc4_264–466/1367–1542, E1475Q, W1440A_–8 × HIS	This work	N/A
*C. thermophilum* Brn1_765–898_ in complex with Smc4_264–466/1367–1542, S1447R_–8 × HIS	This work	N/A
*C. thermophilum* Brn1_765–898_ in complex with Smc4_264–466/1367–1542, S1439D, W1440A_–8 × HIS	This work	N/A
*C. thermophilum* Brn1_765–898_ in complex with Smc4_264–466/1367–1542, F1482A, R1483D_–8 × HIS	This work	N/A
*C. thermophilum* Brn1_765–898_ in complex with Smc4_264–466/1367–1542, W1440A_–8 × HIS	This work	N/A
*C. thermophilum* 6 × HIS–Ycs4_3–1222_	modified from Piazza et al., 2014	N/A
*C. thermophilum* 6 × HIS–Ycs4_3-–827_	modified from Piazza et al., 2014	N/A
*C. thermophilum* 6 × HIS–Ycs4_3–689_	modified from Piazza et al., 2014	N/A
*C. thermophilum* 6 × HIS–Ycs4_3-–518_	modified from Piazza et al., 2014	N/A
*C. thermophilum* 6 × HIS–Brn1_225–512_ in complex with Ycs4_3–1222_	This work	N/A
*C. thermophilum* 6 × HIS–Brn1_336-512_ in complex with Ycs4_3–1222_	This work	N/A
*C. thermophilum* 6 × HIS–Brn1_225–512_	This work	N/A
*C. thermophilum* 6 × HIS–Brn1_336–512_	This work	N/A
*C. thermophilum* 6 × HIS–Brn1_225–418_ in complex with Ycs4_3–828, 869–915, 939–1222_	This work	N/A
*C. thermophilum* 6 × HIS–Brn1_225-418_ in complex with Ycs4_3–828, 869–915, 939–1222, K1094D, V1095S, K1096D, Q1098D, L1099S_	This work	N/A
*C. thermophilum* GST–Brn1_225–340_	modified from Kschonsak et al., 2017	N/A
*C. thermophilum* GST Brn1_225–512_	modified from Kschonsak et al., 2017	N/A
*C. thermophilum* GST Brn1_336–512_	modified from Kschonsak et al., 2017	N/A
*C. thermophilum* GST Brn1_336–714_	modified from Kschonsak et al., 2017	N/A
*C. thermophilum* GST Brn1_513–714_	modified from Kschonsak et al., 2017	N/A
*C. thermophilum* GST Brn1_636–714_	modified from Kschonsak et al., 2017	N/A
*S. cerevisiae* Smc2 in complex with Smc4–3 × StrepII, Brn1–12 × HIS–3 × HA, Ycg1, Ycs4	modified from Kschonsak et al., 2017	N/A
*S. cerevisiae* Smc2 in complex with Smc4–3 × StrepII, Brn1–12 × HIS–3 × HA, Ycg1, Ycs4_K1049D, V1050S, K1051D, Q1053D, L1054S_	This work	N/A
*S. cerevisiae* Smc2_Q147L_ in complex with Smc4_Q302L_–3 × StrepII, Brn1–12 × HIS–3 × HA, Ycg1, Ycs4	This work	N/A
*S. cerevisiae* Smc2 in complex with Smc4–3 × StrepII, Brn1(ybbR tag replacing residues 13–23, 3 × TEV site inserted at residue 141)–12 × HIS–3 × HA, Ycg1, Ycs4	Ganji et al., 2018	N/A
*S. cerevisiae* Smc2 in complex with Smc4–3 × StrepII, Brn1(ybbR tag replacing residues 13–23, 3 × TEV site inserted at residue 141)–12 × HIS–3 × HA tag, Ycs4	This work	N/A
*S. cerevisiae* Smc2 in complex with Smc4–3 × StrepII, Brn1(ybbR tag replacing residues 13–23, 1 × TEV sites inserted at residues 141 and 373)– 12 × HIS-3 × HA, Ycg1, Ycs4	This work	N/A
*S. cerevisiae* Smc2_Q147L_ in complex with Smc4_Q302L_–3 × StrepII, Brn1(ybbR tag replacing residues 13–23, 3 × TEV site inserted at residue 141)–12 × HIS–3 × HA, Ycg1, Ycs4	This work	N/A
*S. cerevisiae* Smc2_S1085R_ in complex with Smc4_S1324R_–3 × StrepII, Brn1(ybbR tag replacing residues 13–23, 3 × TEV site inserted at residue 141)–12 × HIS–3 × HA, Ycg1, Ycs4	This work	N/A
*S. cerevisiae* Smc2_E1113Q_ in complex with Smc4_E1352Q–_3 × StrepII, Brn1(ybbR tag replacing residues 13–23, 3 × TEV-site inserted at residue 141)–12 × HIS–3 × HA, Ycg1, Ycs4	This work	N/A

**Deposited Data**		

*C. thermophilum* Smc2_hd_ (I)	This work	PDB: 6QJ1
*C. thermophilum* Smc2_hd_ (II)	This work	PDB: 6QJ0
*C. thermophilum* Smc4_hd_–Brn1_C_	This work	PDB: 6QJ2
*C. thermophilum* Ycs4–Brn1_Y4_	This work	PDB: 6QJ3
*C. thermophilum* Ycs4–Brn1_Y4_–Smc4_hd_–Brn1_C_	This work	PDB: 6QJ4
*C. thermophilum* Smc2_cc_–Brn1_N_	This work	PDB: 6Q6E
NMR chemical shifts and restraints	This work	BMRB: 34336
Original image files	This work	https://doi.org/10.17632/rk9hdmj8tk.1

**Experimental Models: Cell Lines**		

HeLa Kyoto H2B-mCherry	Neumann et al., 2010	N/A

**Experimental Models: Organisms/Strains**		

See Table S3		

**Oligonucleotides**		

*rDNA* 5′-TTTCTGCCTTTTTCGGTGAC-3′	Cuylen et al., 2011	Oligo SC41
*rDNA* 5′-TGGCATGGATTTCCCTTTAG-3′	Cuylen et al., 2011	Oligo SC42
*CEN5* 5′-AGCAGTATTAGATTTCCGAAAAGA-3′	This work	Oligo SC71
*CEN5* 5′-CGTTTAGTTTTTCTTTTCCTTTTCTTG-3′	This work	Oligo SC72

**Recombinant DNA**		

See Table S1		

**Software and Algorithms**		

X-ray Detector Software (XDS)	Kabsch, 2010	http://xds.mpimf-heidelberg.mpg.de/
autoSHARP	Vonrhein et al., 2007	https://www.globalphasing.com
Phenix suite	Adams et al., 2010	https://www.phenix-online.org/
CCP4 suite	Winn et al., 2011	http://www.ccp4.ac.uk/
SBGrid DEN/CNS	O’Donovan et al., 2012	https://portal.sbgrid.org/d/apps/den/
COOT v0.8.2	Emsley et al., 2010	https://www2.mrc-lmb.cam.ac.uk/personal/pemsley/coot/
PyMOL	Schrödinger, LLC	https://www.pymol.org/2/
ConSurf	Ashkenazy et al., 2016	http://bental.tau.ac.il/new_ConSurfDB/
APBS	Baker et al., 2001	http://www.poissonboltzmann.org/

### Contact for Reagent and Resource Sharing

Further information and requests for resources and reagents should be directed to and will be fulfilled by the Lead Contact, Christian H. Haering (christian.haering@embl.de).

### Experimental Model and Subject Details

#### Cell Lines

HeLa Kyoto H2B-mCherry cells (Neumann et al., 2010) were cultivated in DMEM (Life Technologies) containing 10% FBS (Life Technologies), 1% PenStrep (Invitrogen), and 1% glutamine (Invitrogen) at 37°C, 5% CO_2_.

#### Yeast Strains

*Saccharomyces cerevisiae* strains are derived of W303. Genotypes are listed in the Table S3.

#### Bacterial Strains

Proteins for crystallography and biochemistry were expressed in *Escherichia coli* Rosetta (DE3) pLysS cells (Merck, 70954) pre-grown at 37°C and grown at 18°C for induction in 2 × TY or Terrific Broth (TB) medium.

### Method Details

#### Protein Expression and Purification

Expression of *Ct* Ycs4, *Ct* Ycs4–Brn1, or *Ct* Smc2_hd_–Brn1_N_ constructs (see Table S1) was induced for 18 h from pET-MCN vectors (Romier et al., 2006) in *Escherichia coli* Rosetta (DE3) pLysS (Merck) grown at 18°C in 2 × TY medium or M9 minimal medium (for NMR). The *Ct* Smc4_hd_–Brn1_C_ constructs were expressed in Sf21 cells using the Multibac expression system (Fitzgerald et al., 2006). Cells were lysed by sonication at 4°C in lysis buffer (50 mM TRIS-HCl pH 7.5, 200–500 mM NaCl, 20 mM imidazole, 5 mM β-mercaptoethanol) containing cOmplete protease inhibitor cocktail tablets without EDTA (cOm–EDTA, Roche). The lysate was cleared by centrifugation at 45,000 × g_max_ and loaded onto Ni-Sepharose 6FF (GE Healthcare). After washing with 30–40 column volumes (cv) lysis buffer, proteins were eluted in 7–10 cv elution buffer (lysis buffer plus 300 mM imidazole). The eluate was dialyzed overnight in dialysis buffer (25 mM TRIS-HCl pH 7.5, 200–300 mM NaCl, 1 mM DTT) at 4°C and amino-terminal His_6_-tags were cleaved by addition of TEV protease, where applicable.

*Ct* Smc2_hd_–Brn1_N_ was loaded onto a Superdex 200 26/60 column (GE Healthcare) equilibrated in SEC-buffer (25 mM TRIS-HCl pH 7.5, 200 mM NaCl, 1 mM DTT). Peak fractions were pooled and diluted with low-salt buffer (25 mM TRIS-HCl pH 7.5, 100 mM NaCl, 1 mM DTT) to a final salt concentration of 100 mM NaCl and loaded onto a 1.7-mL Source S (GE Healthcare) cation exchange column pre-equilibrated with low-salt buffer. After washing with 3–5 cv low-salt buffer, proteins were eluted by increasing NaCl concentrations to 1 M in a linear gradient of 60 mL. Peak fractions were pooled and concentrated by ultrafiltration (Vivaspin 10,000 MWCO, Sartorius).

*Ct* Smc4_hd_–Brn1_C_ was loaded onto a Superdex 200 26/60 column (GE Healthcare) equilibrated in SEC-buffer (25 mM TRIS-HCl pH 7.5, 300 mM NaCl, 1 mM DTT). Peak fractions were pooled and diluted with low-salt buffer (25 mM TRIS-HCl pH 7.5, 100 mM NaCl, 1 mM DTT) to a final salt concentration of 100 mM NaCl and loaded onto a 6-mL Resource Q (GE Healthcare) anion exchange column pre-equilibrated with low-salt buffer. Flow through fractions containing *Ct* Smc4_hd_–Brn1_C_ were pooled and concentrated by ultrafiltration (Vivaspin 10,000 MWCO, Sartorius).

*Ct* Ycs4 and *Ct* Ycs4–Brn1 were diluted with low-salt buffer (25 mM TRIS-HCl pH 7.5, 100 mM NaCl, 1 mM DTT) to a final salt concentration of 150 mM NaCl and loaded onto a 6 mL RESOURCE Q (GE Healthcare) anion exchange column pre-equilibrated with low-salt buffer. After washing with 3–5 cv low-salt buffer, proteins were eluted by increasing NaCl concentrations to 1 M in a linear gradient of 60 mL. Peak fractions were pooled and loaded onto a Superdex 200 26/60 column (GE Healthcare) equilibrated in SEC-buffer (25 mM TRIS-HCl pH 7.5, 500 mM NaCl, 1 mM DTT). Peak fractions were pooled and concentrated by ultrafiltration (Vivaspin 30,000 MWCO, Sartorius).

His_6_-tagged *Ct* Brn1 fragments spanning residues 225–512 or 336–512 where co-expressed and co-purified with untagged *Ct* Ycs4 using the above protocol. Excess *Ct* Brn1 was separated from the *Ct* Ycs4–Brn1_Y4_ complex during the size-exclusion chromatography step.

*Ct* Smc2_cc_–Brn1_N_ fusion protein (NMR) was dialyzed overnight in dialysis buffer (10 mM Na/K phosphate pH 6.5, 50 mM NaCl, 1 mM DTT) at 4°C and the amino-terminal His_6_-tag was cleaved by addition of TEV protease before loading onto a Superdex 75 26/60 column (GE Healthcare) equilibrated in SEC-buffer (10 mM Na/K phosphate pH 6.5, 50 mM NaCl, 1 mM DTT). Peak fractions were pooled and concentrated by ultrafiltration (Vivaspin 10,000 MWCO, Sartorius).

Various *Ct* Brn1 fragments (see Table S1) were expressed as amino-terminal GST fusion constructs from a pGEX6P-1 vector (Smith and Johnson, 1988) as described above. Cells were lysed at 4°C by sonication in lysis buffer (50 mM TRIS-HCl pH 7.5, 500 mM NaCl, 2 mM DTT containing cOm–EDTA). The lysate was cleared by centrifugation at 45,000 × g _max_ and loaded onto Glutathione Sepharose 4B beads (GE Healthcare). The GST-fusion protein was eluted from the beads with lysis buffer containing 10 mM L-glutathione. The eluate was dialyzed and purified over RESOURCE Q as described above. Peak fractions were pooled and concentrated by ultrafiltration (Vivaspin 10,000 MWCO).

Condensin holocomplexes were expressed (St-Pierre et al., 2009) and purified as described previously (Kschonsak et al., 2017). YbbR-tagged holocomplexes were covalently coupled to Coenzyme A-ATTO488 (New England Biolabs) with Sfp synthase as described previously (Ganji et al., 2018). Selenomethionine-labeled *Ct* Ycs4–Brn1_Y4_ and *Ct* Smc2_hd_–Brn1_N_ were expressed applying methionine pathway inhibition (Doublié, 1997) and purified as described above.

#### Crystallization and Data Collection

Crystals of selenomethionine-labeled *Ct* Smc2_hd_ (form I) (Table 1) were grown at 20°C by hanging-drop vapor diffusion. A volume of 1 μL protein *Ct* Smc2hd_2-224/983-1179_–Brn1_112-204_ (8 mg/mL in 25 mM TRIS-HCl 7.5, 100 mM NaCl, 1 mM DTT) was mixed with 1 μL crystallization solution (18% (w/v) PEG3350, 0.2 M Succinate pH 7.0, 2 mM MnCl_2_). Crystals were cryo-protected by addition of crystallization solution containing 20% (v/v) glycerol before flash freezing in liquid nitrogen. Single-wavelength anomalous dispersion data were collected at a wavelength of 0.979 Å (peak) at beamline ID29, European Synchrotron Radiation Facility (ESRF, Grenoble, France) (de Sanctis et al., 2012). Data were processed with XDS and XSCALE (Kabsch, 2010).

*Ct* Smc2_hd_ crystals (form II) (Table 1) were grown by hanging-drop vapor diffusion after mixing 1 μL protein Smc2_2–215/990–1179_– Brn1_112-204_ (12.7 mg/mL in 25 mM TRIS-HCl pH 7.5, 100 mM NaCl, 1 mM DTT) and 1 μL crystallization solution (10%–17% (w/v) PEG 8,000, 0.1 M Na cacodylate pH 6.0) at 20°C. Crystals were cryo-protected by addition of 20% (v/v) glycerol before flash freezing in liquid nitrogen. The dataset was collected at a wavelength of 0.976 Å at beamline ID29, ESRF (de Sanctis et al., 2012). Data were processed with XDS (Kabsch, 2010) and POINTLESS (Evans, 2011) and scaled with SCALA of the CCP4 suite (Evans and Murshudov, 2013, Winn et al., 2011).

*Ct* Smc4_hd_–Brn1_C_ crystals (Table 1) were grown by hanging-drop vapor diffusion after mixing 1 μL protein Smc4_264–466/1367–1542_–Brn1_765-898_ (6.5 mg/mL in 25 mM TRIS-HCl pH 7.5, 150 mM NaCl, 1 mM DTT, 1 mM MgCl_2_, 1 mM ATPγS) and 1 μL crystallization solution (3% (v/v) EtOH, 0.1 M Na citrate pH 6.0, 1.5 M LiSO_4_) at 20°C. Crystals were cryo-protected in 2 M LiSO_4_ before flash freezing in liquid nitrogen. The dataset was collected at a wavelength of 1.000 Å at beamline ID29, ESRF (de Sanctis et al., 2012). Data were processed as described above.

Native and selenomethionine-labeled *Ct* Ycs4–Brn1_Y4_ crystals (Table 1) were grown by sitting drop vapor diffusion after mixing 100 nL Ycs4_Δloops_–Brn1_225–418_ and 100 nL crystallization solution in an MRC 2-well plate (Hampton Research). Native crystals were harvested after 30 days (5 mg/mL in 10 mM TRIS–HCl pH 7.5, 200 mM NaCl, 1 mM DTT) with crystallization buffer (12% (w/v) PEG 8,000, 0.1 M ADA pH 6.8, 0.1 M NaCl) at 7°C. Selenomethionine-labeled crystals were harvested after 30 days (6 mg/mL in 10 mM TRIS-HCl pH 7.5, 200 mM NaCl, 1 mM DTT) with crystallization buffer (13% (w/v) PEG 8,000, 0.1 M ADA pH 7.1, 0.13 M NaCl) at 7°C. All crystals were flash frozen in liquid nitrogen after addition of 2 μL crystallization buffer containing 37% (v/v) PEG 400. Datasets of selenomethionine-labeled *Ct* Ycs4–Brn1_Y4_ were collected at a wavelength of 0.966 Å at beamline MASSIF1 (ID30A-1), ESRF (Bowler et al., 2015, Svensson et al., 2015). The dataset of native *Ct* Ycs4–Brn1_Y4_ was collected at a wavelength of 1.0 Å at beamline ID29, ESRF (de Sanctis et al., 2012). All datasets were processed as described above using AIMLESS (Evans and Murshudov, 2013).

*Ct* Ycs4–Brn1_Y4_–Smc4_hd_–Brn1_C_ crystals (Table 1) were grown by sitting drop vapor diffusion after mixing 100 nL sample (10 mg/mL complex in 25 mM TRIS–HCl pH 7.5, 150 mM NaCl, 1 mM DTT) and 100 nL crystallization solution (0.1 M TRIS-HCl pH 8.5, 8% PEG 8,000, 1 mM TCEP) in an MRC 2-well plate (Hampton Research) at 7°C. Crystals were cryo-protected by addition of 2 μl crystallization buffer containing 37% (v/v) PEG 400 before flash freezing in liquid nitrogen. The dataset was collected at a wavelength of 0.976 Å at beamline ID30B, ESRF (Mueller-Dieckmann et al., 2015). Data were processed as described above using SCALA (Evans, 2006).

#### Structure Determination and Refinement

Single anomalous dispersion data for *Ct* Smc2_hd_ (crystal form I), was used to locate 16 selenium sites with autoSHARP (Vonrhein et al., 2007) followed by site refinement, phasing, and density modification. An initial model was built using Phenix AutoBuild and manual adjustment in Coot (Emsley et al., 2010, Terwilliger et al., 2008). The model was further improved by iterative rounds of restrained refinement with phenix.refine and manual adjustment with Coot (Afonine et al., 2012, Emsley et al., 2010).

The *Ct* Smc2_hd_ (crystal form II) structure was solved by molecular replacement with an adapted *Ct* Smc2_hd_ (crystal form I) as search model using Phenix Phaser-MR (McCoy, 2007). The structure was finalized in iterative rounds of manual correction with Coot (Emsley et al., 2010) and restrained refinement with phenix.refine (Afonine et al., 2012).

The *Ct* Smc4_hd_–Brn1_C_ structure was solved by molecular replacement with an adapted *Sc* Smc1_hd_–Scc1_C_ structure (pdb 1W1W) as search model using Phenix Phaser-MR (McCoy, 2007). An initial model was built using Phenix AutoBuild and manual adjustments with Coot (Emsley et al., 2010, Terwilliger et al., 2008). The structure was further improved in iterative rounds of manual correction with Coot (Emsley et al., 2010) and restrained refinement with phenix.refine (Afonine et al., 2012).

Single anomalous dispersion data, merged from two independent datasets, and native data for *Ct* Ycs4–Brn1_Y4_ were used to locate 34 selenium sites with Phenix AutoSol (Adams et al., 2010), followed by site refinement, phasing, and density modification. An initial model was built using Phenix AutoBuild and manual adjustment in Coot (Emsley et al., 2010, Terwilliger et al., 2008). The model was further improved by iterative rounds of manual adjustments with Coot (Emsley et al., 2010) and restrained refinements with phenix.refine (Afonine et al., 2012) against the anomalous dataset.

The *Ct* Ycs4–Brn1_Y4_–Smc4_hd_–Brn1_C_ low-resolution co-structure was solved by a molecular replacement search with adapted *Ct* Ycs4–Brn1_Y4_ and *Ct* Smc4_hd_–Brn1_C_ as individual search components using Phenix Phaser-MR (McCoy, 2007). The initial model was refined using the deformable elastic network (DEN) protocol with CNS over a grid-enabled web server hosted by SBGrid (O’Donovan et al., 2012, Schröder et al., 2010) using standard settings and the input structure as both starting and reference models (Brunger et al., 2012). Out of the resulting models, the one with the lowest R_free_ value was used for a final round of manual adjustments with Coot (Emsley et al., 2010) and real-space refinement with phenix.refine (Afonine et al., 2012).

All structures were refined with hydrogens (‘riding’ model) and validated using MolProbity (Chen et al., 2010). Structures (Table 1) have the following Ramachandran statistics: *Ct* Smc2_hd_ (crystal form I) favored 96.0%, outliers 0.2%; *Ct* Smc2_hd_ (crystal form II) favored 99.0%, outliers 0%; *Ct* Smc4_hd_–Brn1_C_ favored 94.0%, outliers 0.5%; *Ct* Ycs4–Brn1_Y4_ favored 92.0%, outliers 0.8%; *Ct* Ycs4–Brn1–Smc4_hd_ favored 90.0%, outliers 0.1%.

Structures were visualized with PyMOL (Schrödinger, LLC). The electrostatic surface potential graph was created with APBS (Baker et al., 2001).

#### NMR spectroscopy and structure calculation

NMR experiments were recorded on Bruker AVIII NMR spectrometers operating at field strengths corresponding to proton Larmor frequencies of 600 and 800 MHz equipped with a cryogenic TXI probe. All spectra were acquired at 298 K, processed with NMRPipe (Delaglio et al., 1995), and analyzed using NMRview (Johnson and Blevins, 1994). Initial backbone assignments were achieved from TROSY-HNCA, -HN(CO)CA, -HNCACB and -HN(CO)CACB recorded on a ^2^H-^13^C-^15^N labeled sample (Pervushin et al., 1997, Salzmann et al., 1998). Backbone and side-chain assignments were completed mainly on a set of 3D NOESY spectra – ^1^H-NOESY-^1^H,^15^N-HSQC, (^1^H),^13^C-HMQC-NOESY-^1^H,^15^N-HSQC, ^1^H,^13^C-HMQC-NOESY-^1^H and (^1^H),^13^C-HMQC-NOESY-^1^H,^13^C-HMQC. The same experiments were used for deriving NOE-based distance restraints to feed structure calculation using CNS1.2 (Brunger, 2007) and ARIA1.2 (Linge et al., 2003). Due to the size and for NMR disadvantageous tumbling behavior of coiled-coil proteins, conventional side chain assignment experiments yielded too low signal-to-noise. Therefore, side chain assignments have been achieved using the above listed NOESY-type experiments. Consequently, NOEs were thereby manually assigned followed by the iterative ARIA approach to quantify, merge, and decrease assignment ambiguities, with the (^1^H),^13^C-HMQC-NOESY-^1^H,^13^C-HMQC data included as a 4D peak list. Backbone torsion angles were determined from chemical shifts using TALOS+ (Shen et al., 2009). Structural quality after refinement of the ten lowest energy structures out of 100 calculated structures in iteration 8 was validated using PROCHECK (Laskowski et al., 1996) and WHATIF (Vriend, 1990) (Table S5).

#### Multiple-sequence alignments

Smc2, Smc4, Brn1 and Ycs4 sequences from 40 divergent species (10 animals, 10 plants, 10 yeasts, 10 protists; Table S2) were aligned with MAFFT (Katoh et al., 2002) using the Smith-Waterman local algorithm (L-INS-i) with default settings. The Ycs4 alignments were used to map surface sequence conservation with Consurf (relaxed conservation scores) (Ashkenazy et al., 2016). To account for the overall higher level of sequence conservation, the column scores of the Smc2, Smc4 or Brn1 alignments were calculated with ClustalX (Larkin et al., 2007) using a PAM 250 matrix, binned to 10 steps and mapped onto *Ct* Smc2_hd_ or *Ct* Smc4 _hd_ surface models (strict conservation scores).

#### ATP Hydrolysis Assays

Reactions (10 μL) were set up with 5 μM SMC head proteins, as indicated, in ATPase buffer (50 mM TRIS-HCl pH 7.5, 215 mM NaCl, 2% (v/v) glycerol, 10 mM MgCl_2_, 5 mM ATP, 1.3 mM DTT and 33 nM [α^32^P]-ATP; Hartmann Analytic). ATP hydrolysis reactions were initiated by addition of ATP and incubated at 30°C. A volume of 1.0 μL of the reaction mix was spotted onto PEI cellulose F TLC plates (Merck) every 3 min for a total of 15 min. The reaction products were resolved on TLC plates using 0.5 M LiCl and 1 M formic acid solution and detected by exposing the TLC plates to a phosphorimager screen and analysis on a Typhoon FLA 9,500 scanner (GE Healthcare). ATP hydrolysis rates were calculated from the ADP/ATP ratios from time points in the linear range of the reaction.

ATPase assays with condensin holocomplexes were carried out as described previously (Kschonsak et al., 2017).

#### Isothermal Titration Calorimetry

*Ct* Smc2_hd_–Brn1_N_ or *Ct* Smc4_hd_–Brn1_C_ proteins were dialyzed to ITC buffer 1 (25 mM TRIS-HCl pH 7.5, 200 mM NaCl, 1 mM MgCl_2_) or ITC buffer 2 (25 mM TRIS-HCl pH 7.5, 200 mM NaCl, 10 mM MgCl_2_, 2% glycerol, 0.5 mM DTT). ATP was dissolved in ITC buffer 1 or buffer 2. ATP was injected at a concentration of 340 μM into 37.5–42.0 μM protein at 25°C (buffer 1) or 190–400 μM into 23.6–40.0 μM protein at 20°C (buffer 2). For the interaction studies of *Ct* Brn1 and *Ct* Ycs4 or of *Ct* Smc4_hd_-Brn1_C_ and *Ct* Ycs4-Brn1_Y4_, proteins were dialyzed against ITC buffer 3 (25 mM TRIS-HCl pH 7.5, 300 mM NaCl, 0.5 mM DTT) and injected at 25°C or 10°C.

ITC measurements were performed on a MicroCal iTC200 or a PEAQ-ITC microcalorimeter (Malvern Panalytical). ITC data were corrected for the dilution heat and fitted with the MicroCal Origin software package applying one set of binding sites model. Standard deviation values of the fit were calculated from the original data points.

#### Analytical Size-Exclusion Chromatography

For *Ct* constructs, aliquots of 80 μL of protein samples at a concentration of 15 μM where incubated (with 1 mM ATP where indicated) on ice for 15 min and injected onto a Superdex 200 Increase 3.2/300 column (GE Healthcare) and separated in a buffer containing 175 mM NaCl, 25 mM TRIS-HCl pH 7.5, 1 mM MgCl_2_ and 1 mM DTT (and 100 μM ATP where indicated) at a flow rate of 0.05 mL/min using the ÄKTA Ettan System (GE Healthcare). Fractions of 100 μL were collected and analyzed by SDS-PAGE and Coomassie staining.

For *Sc* condensin holocomplexes, 20 μL aliquots at a concentration of ∼3 μM were incubated 16 h on ice with 1.5 μg TEV protease in the presence of 1 mM EDTA, 0.2 mM PMSF and 0.01% (v/v) Tween-20. After adjustment to 1.0 μM condensin concentration and 125 mM NaCl, 50 mM KCl, 50 mM TRIS-HCl pH 7.5, 5 mM MgCl_2_ and 1 mM DTT (with 1 mM ATP where indicated), 50 μL was injected onto a Superose 6 Increase 3.2/300 column (GE Healthcare) and separated in same buffer (with 100 μM ATP where indicated) at a flow rate of 0.05 mL/min using the ÄKTA Ettan System (GE Healthcare). Fractions of 100 μL were collected and protein precipitated with 10% (w/v) trichloroacetic acid before SDS-PAGE and silver staining.

#### GST Pulldown

40 μg of glutathione S-transferase (GST) fusion protein was incubated with 60 μg of each untagged protein and 30 μL Glutathione Sepharose 4B (GE Healthcare) in 200 mM NaCl, 50 mM TRIS-HCl pH 7.5, 1 mM DTT, 1 mM MgCl_2_ in a total volume of 0.5 mL for 1 h at 4°C. The beads were gently centrifuged at 1,200 rpm for 3 min and washed 6 times with the same volume of buffer before boiling and analysis by SDS-PAGE.

#### Release of the amino-terminal Brn1fragment

Aliquots of 10-20 μL of ∼3 μM *Sc* condensin holocomplexes with CoA-ATTO488-labeled Brn1 were treated 16 h on ice with 1.5 μg TEV protease in the presence of 1 mM EDTA, 0.2 mM PMSF and 0.01% (v/v) Tween-20. Next, 5.5 pmol of condensin was immobilized on 20 μL Protein A-coupled Dynabeads (ThermoFisher Scientific) that had been pre-bound to 3 μg anti-HA antibody (12CA5). Beads were washed four times with 50 mM TRIS-HCl pH 7.5, 125 mM NaCl, 50 mM KCl, 5 mM MgCl_2_, 5% (v/v) glycerol, 1 mM DTT, 0.2 mM PMSF and 0.01% (v/v) Tween-20. Release of the Brn1 amino-terminal cleavage fragment was subsequently assayed by washing three times with same buffer (including 1 mM ATP where indicated) with 5 min incubation at 25°C each. The three washes were collected and protein precipitated with 10% (w/v) trichloroacetic acid. Proteins bound to beads were eluted in 2 × SDS loading buffer (100 mM TRIS–HCl pH 6.8, 4% (w/v) SDS, 20% glycerol (v/v) 0.2% (w/v) bromophenol blue, 0.2 M DTT) by heating to 65°C for 5 min. Individual proteins were resolved by SDS-PAGE and fluorescence was analyzed in-gel on a Typhoon FLA9500 imager (GE Healthcare) with a 473-nm laser and a 510-nm long pass filter.

#### Condensin Immunoprecipitation and Western Blotting

Immunoprecipitation of endogenous condensin complexes from yeast was performed as described previously (Kschonsak et al., 2017). Yeast strains were grown at 30°C in 2 L YPAD media to an OD_600_ of 1.0, harvested by centrifugation and lysed by cryogenic grinding (SPEX Freezer/Mill 6970) in lysis buffer (50 mM TRIS-HCl pH 8.0, 100 mM NaCl, 2.5 mM MgCl_2_, 0.25% (v/v) Triton X-100, 1 mM DTT, 1 mM PMSF) containing 2 × cOm–EDTA. The lysate was cleared by centrifugation at 20,400 × g_max_ and incubated with 100 μL Protein A-coupled Dynabeads (ThermoFisher Scientific) that had been pre-bound to anti-PK (V5) tag antibody (Abd Serotec, MCA1360) for 2 h at 4°C. Beads were washed with IP buffer (50 mM TRIS-HCl pH 8.0, 100 mM NaCl, 1 mM DTT, 5 mM EDTA, 0.25% (v/v) Triton X-100) and eluted in 20 μL 2 × SDS loading buffer (100 mM TRIS-HCl pH 6.8, 4% (w/v) SDS, 20% glycerol (v/v) 0.2% (w/v) bromophenol blue, 0.2 M DTT) by heating to 90°C for 5 min, prior to SDS-PAGE and Coomassie staining or western blotting with antibodies against the PK (V5) tag (Abd Serotec, MCA1360), *Sc* Ycg1 (Piazza et al., 2014) or α-tubulin (TAT1) (Woods et al., 1989).

#### Bpa Crosslinking

Yeast strains expressing Smc4_bpa_ constructs were generated by plasmid shuffle, replacing a *URA3*-based episomal plasmid containing a wild-type *SMC4* allele in a *smc4Δ* background strain with the *TRP1*-based pLH157 encoding *E. coli* TyrRS and tRNA CUA (Chen et al., 2007) with a *LEU2*-based centromeric plasmid containing an *SMC4*_*bpa*_ allele with an amber stop codon at the indicated position.

For analysis by western blotting, yeast strains were grown in 25 mL –LEU–TRP synthetic drop-out media containing 1 mM *p*-benzoyl-L-phenylalanine (bpa; Bachem 4017646) at 30°C to an OD_600_ of 0.6. Cells were harvested by centrifugation, resuspended in 1 mL PBS in a Petri dish and exposed to a total of 5 J 365-nm light using a Stratalinker 2400 UV cross-linker (∼25 min exposure time) at room temperature. Cells were collected by centrifugation, resuspended in 0.5 mL 100 mM NaOH and incubated for 10 min at room temperature. Cells were collected by centrifugation and lysed in SDS loading buffer (50 mM TRIS-HCl pH 6.8, 2% (w/v) SDS, 10% glycerol (v/v) 0.1% (w/v) bromophenol blue, 0.1 M DTT) by at 65°C for 5 min prior to SDS-PAGE and western blotting with antibodies against the PK (V5) tag (Abd Serotec, MCA1360) or the HA tag (Abcam, ab9110).

For analysis by mass spectrometry, cells were harvested from 1.5 L –LEU–TRP media containing 1 mM *p*-benzoyl-l-phenylalanine at an OD_600_ of 2.9 and resuspended in 250 mL PBS. One half of the sample was exposed in Petri dishes to 10 J 365-nm light (∼50 min exposure time), whereas the other half was kept in the dark. Cells were harvested by centrifugation, washed with 45 mL lysis buffer (50 mM TRIS-HCl pH 8.0, 100 mM NaCl, 2.5 mM MgCl_2_, 0.25 (v/v) % Triton X-100) and resuspended in 15 mL lysis buffer containing 1 mM DTT, 1 mM PMSF and 2 × cOm–EDTA before lysis by cryogenic grinding (SPEX Freezer/Mill 6970). Condensin complexes were immunoprecipitated as described above, using 100 μL Protein A-coupled Dynabeads that had been pre-bound to 10 μg anti-PK antibody. After elution and SDS-PAGE, gels were silver stained using a formaldehyde-free protocol. The cross-linked band and a band at the same height in the –UV control were excised for analysis by mass spectrometry.

#### Mass Spectrometry

Silver-stained bands were excised, chopped into small pieces and transferred to 0.5-mL tubes. For all following steps, buffers were exchanged by two consecutive 15 min incubation steps in 200 μL of acetonitrile, which was removed after each step. Proteins were reduced by the addition of 200 μL of 10 mM DTT, 100 mM (NH_4_)HCO_3_ at 56°C for 30 min and alkylated by the addition of 200 μL of 55 mM iodoacetamide, 100 mM (NH_4_)HCO_3_ for 20 min in the dark. A volume of 50 μL of 1 ng/μL trypsin in 50 mM (NH_4_)HCO_3_ was added and samples were incubated for 30 min on ice and then over night at 37°C. Gel pieces were sonicated for 15 min, spun down and the supernatant was transferred into a glass vial (VDS Optilab, 93908556). The gel pieces were washed once with 50 μL of an aqueous solution of 50% acetonitrile and 1% formic acid and sonicated for 15 min. The combined supernatants were dried and reconstituted in 10 μL of 0.1% (v/v) formic acid.

Peptides were separated using the nanoAcquity UPLC system (Waters) with nanoAcquity trapping (nanoAcquity Symmetry C18, 5 μm, 180 μm × 20 mm) and analytical (nanoAcquity BEH C18, 1.7μm, 75 μm × 200 mm) columns, which were coupled to an LTQ Orbitrap Velos (Thermo Fisher Scientific) using the Proxeon nanospray source. Peptides were loaded for 6 min using a constant flow of solvent A (0.1% formic acid) at 5 μL min^-1^. Peptides were then separated via the analytical column using a constant flow of 0.3 μL min^-1^. The percentage of solvent B (acetonitrile, 0.1% formic acid) was increased from 3 to 10% within 5 min, followed by an increase to 40% within 10 min. Eluting peptides were ionized with a Pico-Tip Emitter 360 μm OD × 20 μm ID (10 μm tip, New Objective), applying a spray voltage of 2.2 kV at 300°C. Peptides were analyzed with an Orbitrap Velos Pro system (Thermo). Full scan MS spectra with a mass range of 300–1,700 m/z were acquired in profile mode with a resolution of 30.000 and a filling time of 500 ms, applying a limit of 106 ions. The 15 most intense ions were fragmented in the LTQ using a normalized collision energy of 40%. Three times 104 ions were selected within 100 ms and fragmented upon accumulation of selected precursor ions. MS/MS data were acquired in centroid mode of multiple charged (2+, 3+, 4+) precursor ions. The dynamic exclusion list was restricted to 500 entries with a maximum retention period of 30 s and relative mass window of 10 ppm. In order to improve the mass accuracy, a lock mass correction using a background ion (m/z 445.12003) was applied.

Data were processed using IsobarQuant (Franken et al., 2015) and Mascot (v2.2.07), including carbamidomethyl (C), acetyl (N-term) and oxidation (M) modifications. The mass error tolerance for full scan MS spectra was set to 10 ppm and for MS/MS spectra to 0.02 Da. A maximum of 2 missed cleavages were allowed. A minimum of two unique peptides with a peptide length of at least seven amino acids and a false discovery rate below 0.01 were required on the peptide and protein level (Table S4).

#### ChIP-qPCR

ChIP-qPCR experiments were performed as described previously (Cuylen et al., 2011, Kschonsak et al., 2017). Yeast strains were grown in 42 mL YPAD at 30°C to an OD_600_ of 0.6 and fixed with 4.7 mL fixation buffer (9.5 mM TRIS-HCl pH 8.0, 19 mM NaCl, 0.095 mM EGTA, 3% (v/v) formaldehyde, 0.19 mM EDTA) for 30 min at 16°C. Fixation was stopped by addition of glycine to 125 mM (final concentration), followed by washing steps in PBS and PIPES buffer (100 mM PIPES-KOH pH 8.3). Cells were lysed by spheroplasting with 0.5 mg/mL zymolase T-100 (AMS Biotechnology) in HEMS buffer (100 mM HEPES-KOH pH 7.5, 1 mM EGTA, 1 mM MgSO_4_, 1.2 M Sorbitol, 1 mM PMSF) containing cOm–EDTA, followed by resuspension of cells in 1.5 mL lysis buffer (50 mM HEPES-KOH pH 7.5, 140 mM NaCl, 1 mM EDTA, 1% (v/v) Triton X-100, 0.1% (w/v) sodium deoxycholate, 1 mM PMSF) containing cOm–EDTA. Chromatin was sheared by sonication to a length of ∼500 bp using a Bioruptor UCD-200 (Diagenode) for 9 min, ‘high level’ setting (30 s on, 60 s off).

Lysates were cleared by centrifugation at 16,800 × g_max_ and pre-cleared with 50 μL Protein A Dynabeads (ThermoFisher Scientific) for 90 min at 4°C. 10% of the cleared lysate was used to check sonication, 12% was kept on ice as input sample. 2 μg anti-PK (V5) tag antibody (Abd Serotec MCA1360) was added to the remaining lysate and samples were incubated at 4°C for 16 h before addition of 100 μl Protein A Dynabeads (ThermoFisher Scientific) for another 4 h at 4°C. Beads were washed with lysis buffer, wash buffer (10 mM TRIS-HCl pH 8.0, 0.25 M LiCl, 0.5% (w/v) sodium deoxycholate, 1 mM EDTA, 1 mM PMSF) containing cOm–EDTA and TE buffer (10 mM TRIS-HCl pH 8.0, 1 mM EDTA) containing cOm–EDTA. Samples were eluted in 320 μl TES buffer (50 mM TRIS–HCl pH 8.0, 10 mM EDTA, 1% (w/v) SDS) at 65°C for 8 h. After addition of 30 μg RNaseA (Roche) for 90 min at 37°C and 200 μg Proteinase K (Roche) for 90 min at 65°C, DNA was purified via a spin column (QIAGEN) and eluted in 50 μl EB buffer.

qPCR reactions were set up for 5 μl of 1:5 and 1:25 dilutions for immunoprecipitated samples and 1:5, 1:50, 1:500 and 1:5,000 dilutions for input samples with SYBR green PCR Master mix (Applied Biosystems) and 5 μM qPCR primers (see Key Resources Table) on an Applied Biosystems 7,500 Fast Real-Time PCR System. Data were calculated from two independent experiments with two qPCR runs each.

#### Microscopy of Human Condensin Complexes

HeLa Kyoto H2B-mCherry cells (Neumann et al., 2010) were transiently transfected with pC1 FLAG-EGFP-NCAPH or SMC4-FLAG-EGFP as described previously (Kschonsak et al., 2017).

Images were analyzed with Fiji (Schindelin et al., 2012). First, background was subtracted using the rolling ball algorithm. Chromatin regions were segmented based on the mCherry fluorescence signal and the whole cell was segmented based on the bright field image. Cytoplasmic regions were selected after subtracting the areas of chromatin from the whole cell regions. Mean fluorescence intensities of EGFP images were measured for chromatin and cytoplasmic regions. Data were calculated from two independent experiments. Cells were tested for mycoplasma contamination.

### Quantification and Statistical Analysis

Statistical details of experiments can be found in the figure legends or Method Details section.

### Data and Software Availability

The accession numbers for the coordinate files reported in this paper are PDB: 6Q6E, 6QJ0, 6QJ1, 6QJ2, 6QJ3, 6QJ4. The accession number for the NMR chemical shifts and restraints reported in this paper is BMRB: 34336. Original image files are available at Mendeley Data: https://doi.org/10.17632/rk9hdmj8tk.1.

## Supplemental Information

Document S1. Figures S1–S7 and Tables S1–S5

Document S2. Article plus Supplemental Information
