# Supplementary material for: Structural Basis of an Asymmetric Condensin ATPase Cycle
Source: Mol Cell. 2019 Jun 20;74(6):1175–1188.e9. doi: 10.1016/j.molcel.2019.03.037 (PMC6591010; doi:10.1016/j.molcel.2019.03.037)
Supplement: Document S1. Figures S1–S7 and Tables S1–S5 [file mmc1.pdf]

Molecular Cell, Volume *74*

Supplemental Information

Structural Basis of an Asymmetric Condensin ATPase Cycle

Markus Hassler, Indra A. Shaltiel, Marc Kschonsak, Bernd Simon, Fabian Merkel, Lena Thärichen, Henry J. Bailey, Jakub Macošek, Sol Bravo, Jutta Metz, Janosch Hennig, and Christian H. Haering

Molecular Cell, Volume *74*

Supplemental Information

Structural Basis of an Asymmetric Condensin ATPase Cycle

Markus Hassler, Indra A. Shaltiel, Marc Kschonsak, Bernd Simon, Fabian Merkel, Lena Thärichen, Henry J. Bailey, Jakub Macošek, Sol Bravo, Jutta Metz, Janosch Hennig, and Christian H. Haering

SUPPLEMENTAL FIGURES

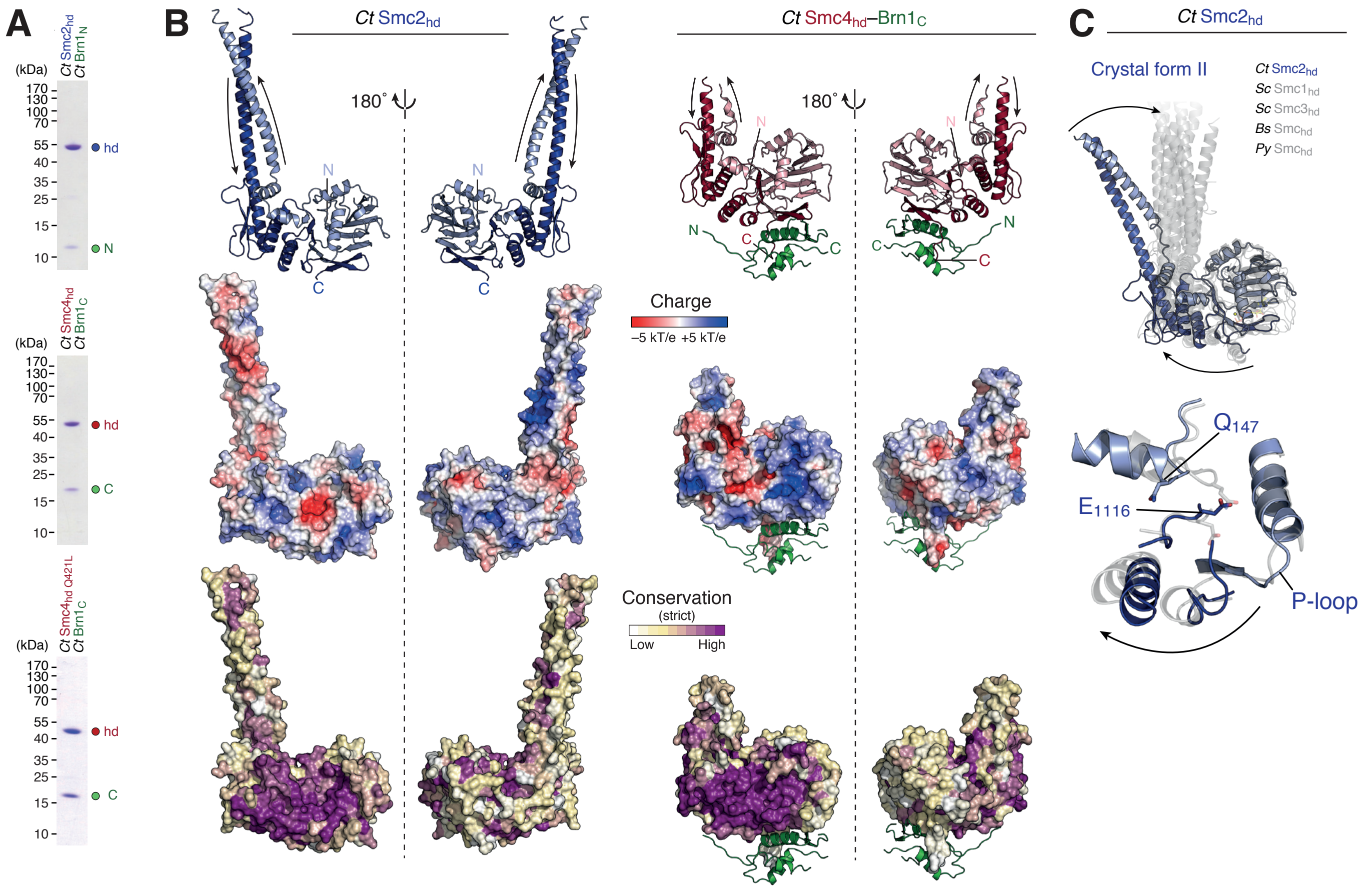

Figure S1. Structures of Smc2 and Smc4 ATPase Head Domains (related to Figure 1)

A Coomassie-stained SDS-PAGE lanes of wild-type *Ct* $Smc2_{hd}$–$Brn1_N$ and wild-type or Q-loop mutant *Ct* $Smc4_{hd}$–$Brn1_C$ complexes used for crystallization and ITC. **B** Cartoon, electrostatic surface potential and surface conservation models of *Ct* $Smc2_{hd}$ and *Ct* $Smc4_{hd}$–$Brn1_C$. **C** Structural alignment based on the RecA lobe of *Ct* $Smc2_{hd}$ (crystal form II) to ATPγS-bound structures of the *Sc* cohesin Smc1 (pdb 1W1W, C_α RMSD = 0.866) and Smc3 (pdb 4UX3, C_α RMSD = 2.880) or the nucleotide-free structures of *B. subtilis* (*Bs*) SMC (pdb 3ZGX, C_α RMSD = 2.866) and *P. yayanosii* (*Py*) SMC (pdb 5XEI, C_α RMSD = 0.988). Close-up views highlight the position of the conserved Q-loop glutamine and Walker B glutamate residues.

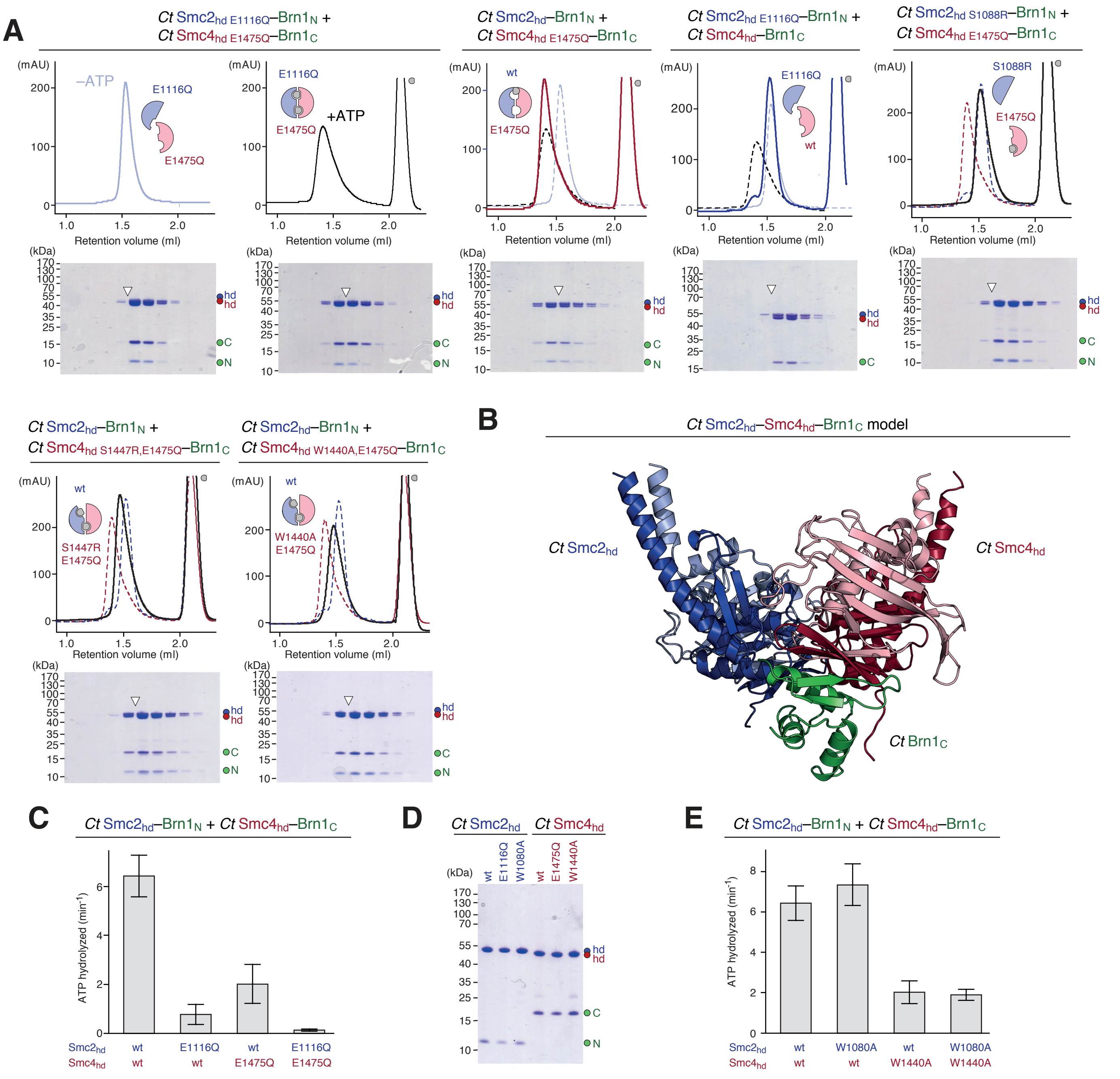

Figure S2. Smc2–Smc4 ATPase Head Dimerization (related to Figure 1)

A Size exclusion chromatography and Coomassie-stained SDS-PAGE analysis of elution fractions of wild-type, Walker B, signature motif or W-loop mutant combinations of *Ct* $Smc2_{hd}$–$Brn1_N$ and *Ct* $Smc4_{hd}$–$Brn1_C$. Dotted lines indicate elution profiles of *Ct* $Smc2_{hd\ E1116Q}$–$Brn1_N$ and *Ct* $Smc4_{hd\ E1475Q}$–$Brn1_C$ in the presence (black) or absence (light blue) of ATP, *Ct* $Smc2_{hd\ E1116Q}$–$Brn1_N$ and *Ct* $Smc4_{hd}$–$Brn1_C$ (blue) or *Ct* $Smc2_{hd}$–$Brn1_N$ and *Ct* $Smc4_{hd\ E1475Q}$–$Brn1_C$ (red) combinations. **B** Model of a *Ct* $Smc2_{hd}$–*Ct* $Smc4_{hd}$–$Brn1_C$ complex built on the ATPγS-dimerized *Sc* $Smc1_{hd}$–$Scc1_C$ homodimer structure (pdb 1W1W). **C** ATPase assays with wild-type or Walker B mutant combinations of *Ct* $Smc2_{hd}$–$Brn1_N$ and *Ct* $Smc4_{hd}$–$Brn1_C$ (mean ± SD of 3 independent experiments). **D** Coomassie-stained SDS-PAGE of wild-type, Walker B or W-loop mutants of *Ct* $Smc2_{hd}$–$Brn1_N$ and *Ct* $Smc4_{hd}$–$Brn1_C$. **E** ATPase assays with wild-type and W-loop mutant combinations of purified *Ct* $Smc2_{hd}$–$Brn1_N$ and *Ct* $Smc4_{hd}$–$Brn1_C$ complexes (mean ± SD of 3 independent experiments).

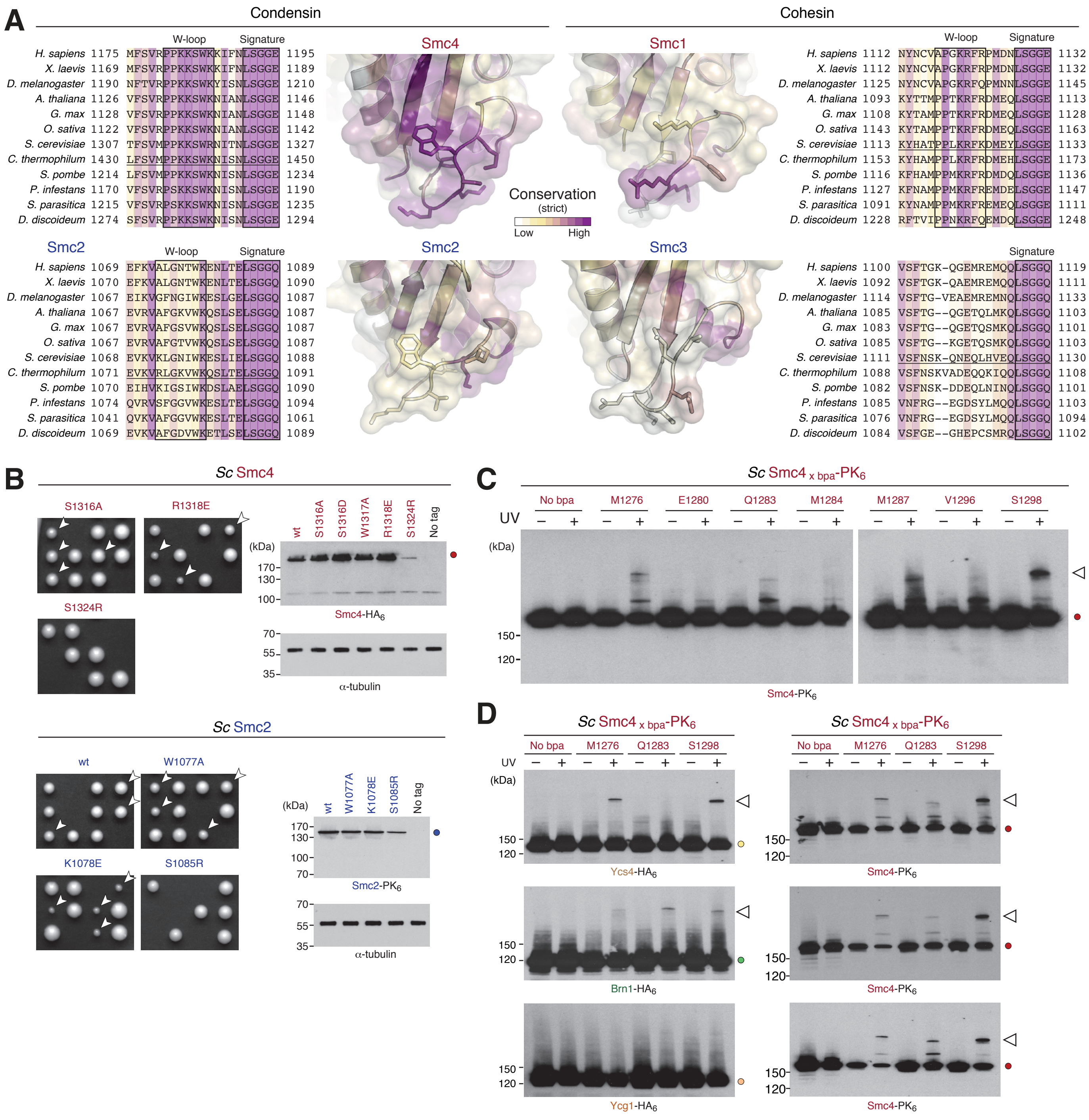

Figure S3. Conservation and Functional Analysis of the Smc4 W-loop (related to Figure 2)

A Partial alignment of W-loop sequences in Smc2 and Smc4 condensin and Smc1 and Smc3 cohesin subunits from 12 divergent species. Colors indicate conservation scores calculated from an alignment of sequences from 40 species (Table S1) and mapped onto the structures of *Ct* $Smc4_{hd}$, *Ct* $Smc2_{hd}$, *Sc* $Smc1_{hd}$ (pdb 1W1W) or *Sc* $Smc3_{hd}$ (pdb 4UX3). **B** Spores of diploid *S. cerevisiae SMC4/smc4Δ* strains expressing an ectopic HA_6-tagged copy of W-loop mutants $Smc4_{S1316A}$ (C4592), $Smc4_{R1318E}$ (C4590) or signature motif mutant $Smc4_{S1324R}$ (C4589) or *SMC2/smc2Δ* strains expressing an ectopic PK_6-tagged copy of W-loop mutants $Smc2_{W1077A}$ (C4567) or $Smc2_{K1078E}$ (C4608) or signature motif mutant $Smc2_{S1085R}$ (C4582) were dissected and incubated for 3 days at 30°C. Protein expression levels were tested by western blotting of whole cell extracts against HA or PK epitope tags. **C** Western blot analysis of Smc4 cross-linking products by probing against the PK epitope of whole cells extracts of strains (C4656, C4672, C4669, C4657, C4670, C4671, C4673, C4681) expressing Smc4-PK_6 with bpa substitutions at the indicated position before (–UV) or after (+UV) exposure to 365-nm light. **D** As in C, of strains co-expressing bpa-substituted versions of Smc4 with endogenously HA_6-tagged versions of Ycs4 (C4715, C4697, C4700, C4754), Brn1 (C4713, C4695, C4698, C4752) or Ycg1 (C4714, C4696, C4699, C4753).

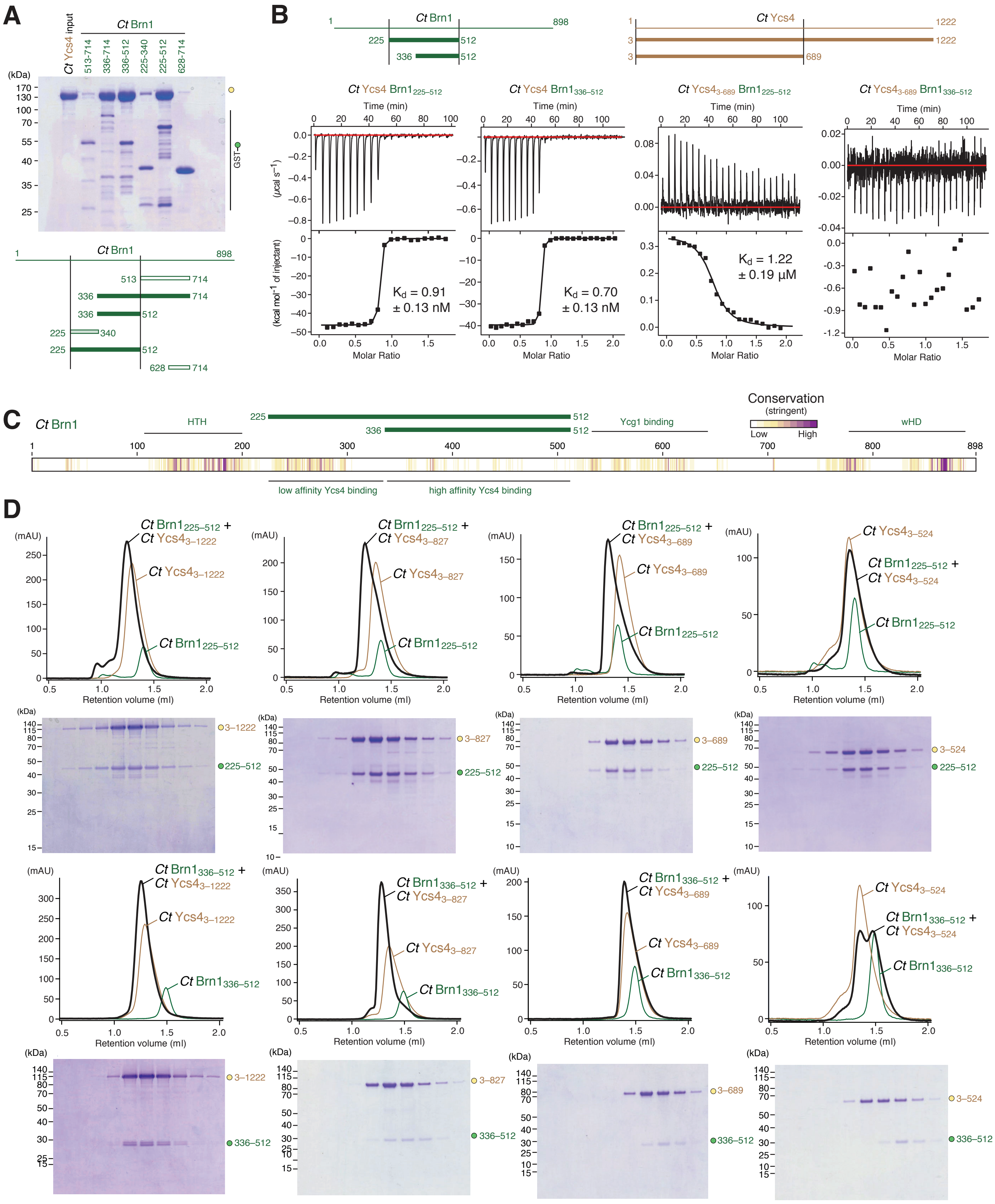
A
B
C
D
Ct Ycs4 input
Ct Brn1
513-714
336-714
336-512
225-340
225-512
628-714
(kDa)
GST-
Ct Brn1
Ct Ycs4
Ct Ycs4 Brn1225–512
Ct Ycs4 Brn1336–512
Ct Ycs43-689 Brn1225–512
Ct Ycs43-689 Brn1336–512
Time (min)
Molar Ratio
(μcal s−1)
(kcal mol−1 of injectant)
Kd = 0.91 ± 0.13 nM
Kd = 0.70 ± 0.13 nM
Kd = 1.22 ± 0.19 μM
Ct Brn1
HTH
Ycg1 binding
wHD
Conservation (stringent)
Low
High
low affinity Ycs4 binding
high affinity Ycs4 binding
(mAU)
Retention volume (ml)
Ct Brn1225–512 + Ct Ycs43–1222
Ct Brn1225–512 + Ct Ycs43–827
Ct Brn1225–512 + Ct Ycs43–689
Ct Brn1225–512 + Ct Ycs43–524
Ct Brn1336–512 + Ct Ycs43–1222
Ct Brn1336–512 + Ct Ycs43–827
Ct Brn1336–512 + Ct Ycs43–689
Ct Brn1336–512 + Ct Ycs43–524
3–1222
3–827
3–689
3–524
225–512
336–512

(Previous page)

Figure S4. Mapping of *Ct* Ycs4–Brn1 Interaction Domains (related to Figure 3)

A Binding of purified *Ct* Ycs4 protein (input) to glutathione beads pre-bound with various GST-*Ct* Brn1 fragments tested by SDS-PAGE and Coomassie staining of bound fractions. **B** ITC of the binding of full-length *Ct* Ycs4$_{3\text{-}1222}$ or truncated Ycs4$_{3\text{-}689}$ to *Ct* Brn1$_{225\text{-}512}$ or *Ct* Brn1$_{336\text{-}512}$ (fit ± error of the fit). **C** Graphical representation of the Brn1 sequence conservation. Colors indicate conservation scores calculated from an alignment of sequences from 40 species (Table S1). Brn1 stretches used for detailed Ycs4 mapping and regions interacting with other condensin subunits are highlighted. **D** Size exclusion chromatography of complexes formed between full-length or truncationed versions of *Ct* Ycs4 and *Ct* Brn1$_{225\text{-}512}$ or *Ct* Brn1$_{336\text{-}512}$. Coomassie-stained SDS-PAGE of elution fractions.

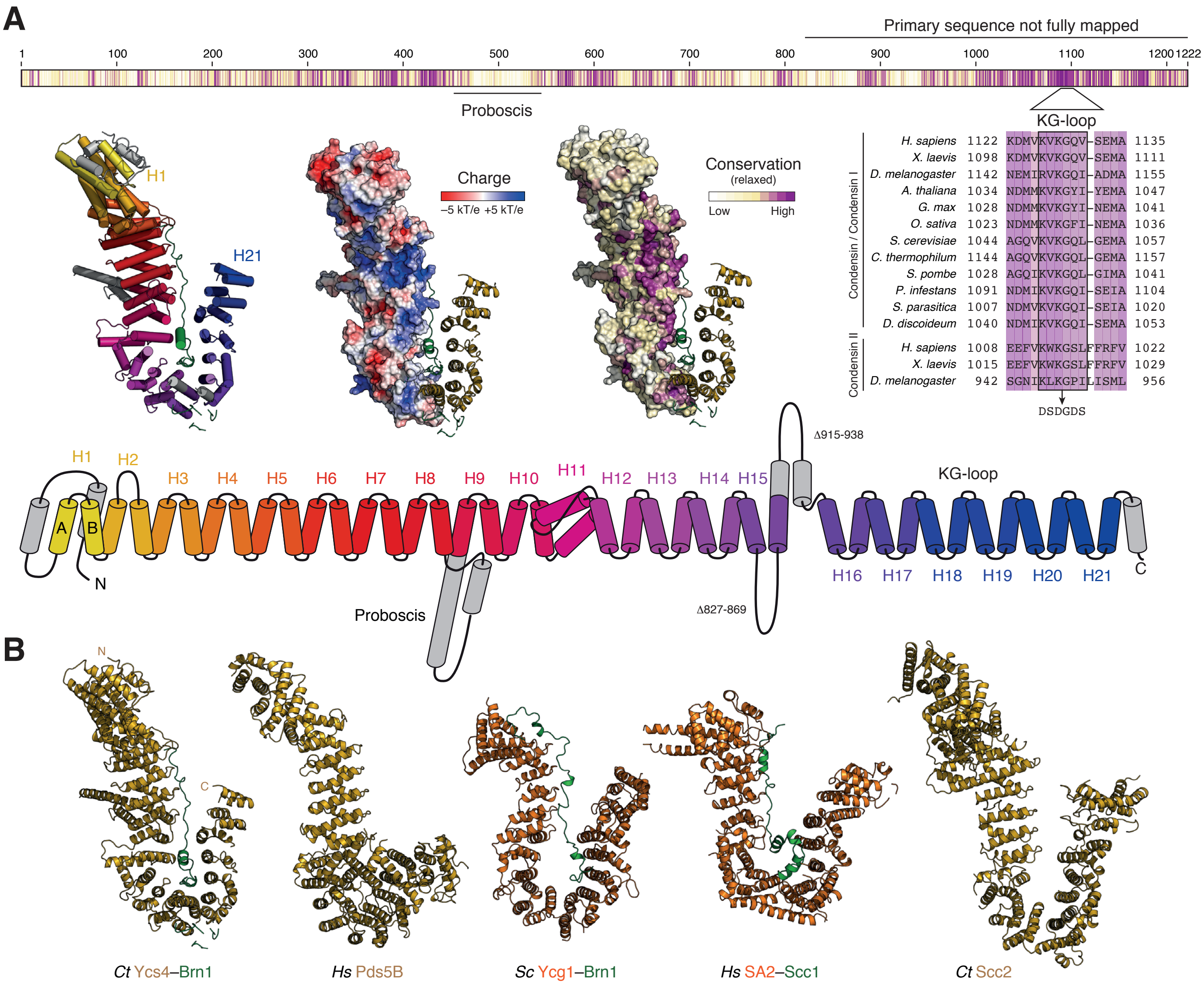

Figure S5. Structure of the *Ct* Ycs4–Brn1$_{Y4}$ Complex (related to Figure 3)

A Cartoon, electrostatic surface potential and surface conservation models of the *Ct* Ycs4–Brn1$_{Y4}$ complex. Surfaces were not built for the carboxy-terminal portion of Ycs4 due to the crystallographic disorder of this part of the complex. The colored box indicates conservation scores in Ycs4 calculated from an alignment of sequences from 40 species and the positions of the Proboscis and conserved KG-loop are highlighted (Table S1). The cartoon model indicates the arrangement of the 21 HEAT-repeat motifs in *Ct* Ycs4 (H1–H21). **B** Side-by-side comparison of the *Ct* Ycs4–Brn1 structure to structures of the *Hs* Pds5 HEAT-repeat subunit and the *Hs* SA2–Scc1 HEAT-repeat-kleisin complex of cohesin (pdb 5HDT, 4PJU), the *Ct* Ycg1–Brn1 HEAT-repeat-kleisin complex of condensin (pdb 5OQQ) and the *Ct* Scc2 subunit of the cohesin loading complex (pdb 5T8V).

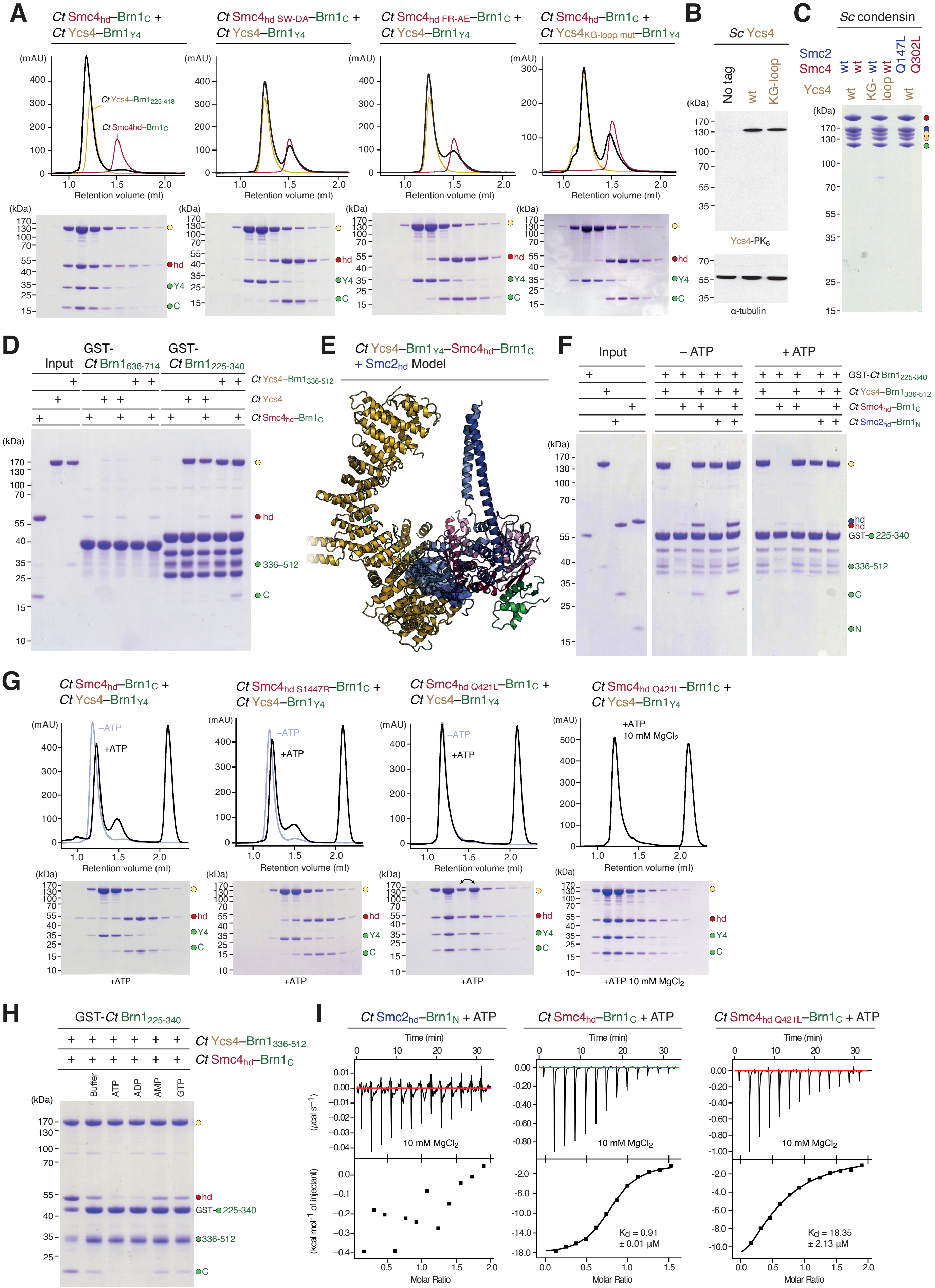
A
Ct Smc4hd–Brn1C +
Ct Ycs4–Brn1Y4
Ct Smc4hd SW-DA–Brn1C +
Ct Ycs4–Brn1Y4
Ct Smc4hd FR-AE–Brn1C +
Ct Ycs4–Brn1Y4
Ct Smc4hd–Brn1C +
Ct Ycs4KG-loop mut–Brn1Y4
Ct Ycs4–Brn1225-418
Ct Smc4hd–Brn1C
(mAU)
Retention volume (ml)
(kDa)
hd
Y4
C
B
Sc Ycs4
No tag
wt
KG-loop
Ycs4-PK6
α-tubulin
C
Sc condensin
Smc2
Smc4
Ycs4
wt
KG-loop
Q147L
Q302L
D
Input
GST-Ct Brn1636-714
GST-Ct Brn1225-340
Ct Ycs4–Brn1336-512
Ct Ycs4
Ct Smc4hd–Brn1C
336–512
E
Ct Ycs4–Brn1Y4–Smc4hd–Brn1C
+ Smc2hd Model
F
Input
– ATP
+ ATP
GST-Ct Brn1225-340
Ct Ycs4–Brn1336-512
Ct Smc4hd–Brn1C
Ct Smc2hd–Brn1N
GST- 225-340
336-512
N
G
Ct Smc4hd–Brn1C +
Ct Ycs4–Brn1Y4
Ct Smc4hd S1447R–Brn1C +
Ct Ycs4–Brn1Y4
Ct Smc4hd Q421L–Brn1C +
Ct Ycs4–Brn1Y4
Ct Smc4hd Q421L–Brn1C +
Ct Ycs4–Brn1Y4
–ATP
+ATP
+ATP 10 mM MgCl2
+ATP
+ATP 10 mM MgCl2
H
GST-Ct Brn1225-340
Ct Ycs4–Brn1336-512
Ct Smc4hd–Brn1C
Buffer
ATP
ADP
AMP
GTP
GST- 225-340
336-512
I
Ct Smc2hd–Brn1N + ATP
Ct Smc4hd–Brn1C + ATP
Ct Smc4hd Q421L–Brn1C + ATP
Time (min)
(μcal s−1)
(kcal mol−1 of injectant)
10 mM MgCl2
Molar Ratio
Kd = 0.91 ± 0.01 μM
Kd = 18.35 ± 2.13 μM

(Previous page)

Figure S6. ATP-dependent Ycs4–Brn1$_{Y4}$ release from Smc2$_{hd}$–Brn1$_{N}$ (related to Figure 4)

A Size exclusion chromatography of complexes formed between *Ct* wild-type, Smc4 W-loop mutant (Smc4$_{S1439D, W1440A}$), Smc4 D-loop mutant (Smc4$_{F1482A, R1483D}$) or Ycs4 KG-loop mutant (Ycs4$_{KG\text{-}loop}$) versions of Smc4$_{hd}$–Brn1$_{C}$ with Ycs4–Brn1$_{225\text{-}418}$. **B** *Sc* Ycs4–PK$_{6}$ protein expression levels tested by western blotting of whole cell extracts against the PK epitope tag (strains C5003, C5005, C5007). **C** Coomassie-stained SDS-PAGE of wild-type, Ycs4 KG-loop and Smc2–Smc4 Q-loop mutant *Sc* condensin holocomplexes used for ATPase assays. **D** Binding of combinations of purified *Ct* Ycs4–Brn1$_{336\text{-}512}$, *Ct* Ycs4 and *Ct* Smc4hd–Brn1$_{C}$ proteins (input) to glutathione beads pre-bound with GST-*Ct* Brn1$_{636\text{-}714}$ or GST-*Ct* Brn1$_{225\text{-}340}$ tested by SDS-PAGE and Coomassie staining of bound fractions. **E** Model of the *Ct* Ycs4–Brn1$_{Y4}$–Smc4$_{hd}$–Brn1$_{C}$ complex bound to *Ct* Smc2$_{hd}$ based on the ATPγS-dimerized *Sc* Smc1$_{hd}$–Scc1$_{C}$ homodimer structure (pdb 1W1W). Steric clashes are indicated by blue surfaces. **F** Binding of purified *Ct* Ycs4–Brn1$_{336\text{-}512}$, *Ct* Ycs4, *Ct* Smc2$_{hd}$–Brn1$_{N}$ and *Ct* Smc4$_{hd}$–Brn1$_{C}$ protein combinations (input) to glutathione beads pre-bound with GST-*Ct* Brn1$_{225\text{-}340}$ in the absence (–ATP) or presence of 600 μM ATP (+ATP) tested by SDS-PAGE and Coomassie staining of bound fractions. **G** Size exclusion chromatography of complexes formed between *Ct* Ycs4–Brn1$_{Y4}$ and wild-type, signature motif mutant (Smc4$_{hd\ S1447R}$) or Q-loop mutant (Smc4$_{hd\ Q421L}$) versions of *Ct* Smc4$_{hd}$–Brn1$_{C}$ in the absence (–ATP) or presence of nucleotide (+ATP), or in the presence of ATP and 10 mM $MgCl_2$. Coomassie-stained SDS-PAGE of elution fraction in the presence of ATP. **H** Effect of different nucleotides on the interaction between *Ct* Smc4–Brn1$_{C}$ and *Ct* Ycs4–Brn1$_{336\text{-}512}$ immobilized on glutathione beads via its binding to GST-*Ct* Brn1$_{225\text{-}340}$. **I** ITC in buffer containing 10 mM $MgCl_2$ of ATP binding by wild-type *Ct* Smc2$_{hd}$–Brn1$_{N}$ and wild-type or Q-loop mutant *Ct* Smc4$_{hd}$–Brn1$_{C}$ (fit ± error of the fit).

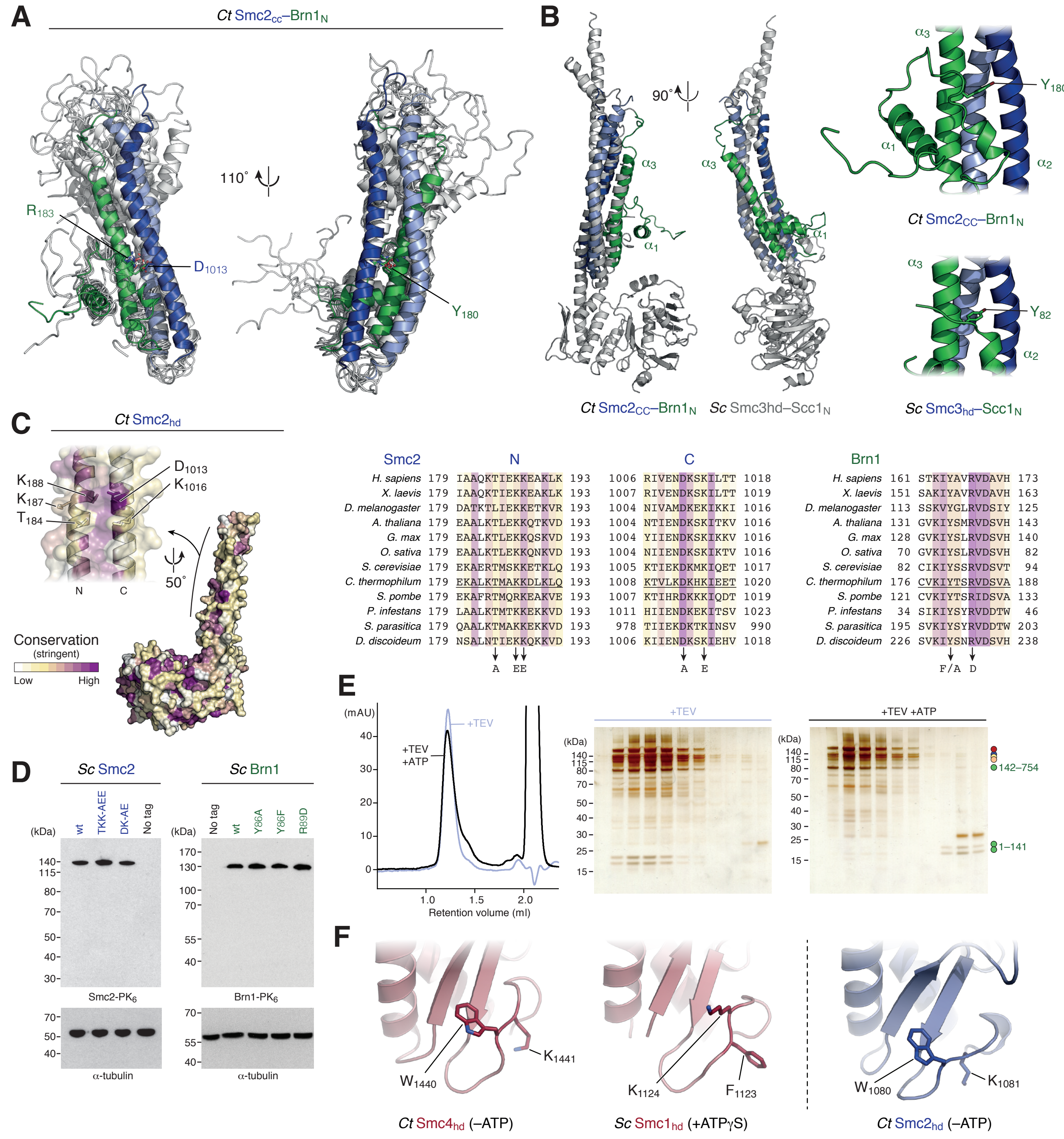

Figure S7. Structure and ATP-dependent release of the $Smc2_{cc}$–$Brn1_N$ from condensin (related to Figures 5 and 6)

A Ensemble of the 10 lowest energy NMR conformers of *Ct* $Smc2_{cc}$–$Brn1_N$. **B** Superimposition of the lowest energy *Ct* $Smc2_{CC}$–$Brn1_N$ NMR structure onto the *Sc* Smc3hd–$Scc1_N$ crystal structure (pdb 4UX3), using Smc2 coiled coil and Brn1 α_3 helices as guide. Close-up views highlight the intercalation of the conserved kleisin tyrosine residues between the SMC coiled coils. **C** Partial alignment of Smc2 amino-terminal (left) and carboxy-terminal (middle) neck regions as well as $Brn1_N$ α_3 regions from 12 divergent species. Colors indicate conservation scores calculated from an alignment of sequences from 40 species (Table S1). **D** Expression levels of wild-type (C5277) and mutant (C5278, C5279) versions of Smc2–PK_6 or of wild-type (C5239) and mutant (C5261–C5263) versions of Brn1–PK_6 in budding yeast probed by western blotting of whole cell extracts against the PK epitope tag. **E** Size exclusion chromatography of purified *Sc* condensin complexes cleaved by TEV protease at position 141 in Brn1 in the absence of nucleotide or presence of ATP. Silver-stained SDS-PAGE of TCA-precipitated elution fractions. **F** Comparison of W-loop residue conformations in nucleotide-free condensin *Ct* Smc2 and *Ct* Smc4 and ATPγS-bound *Sc* Smc1 (pdb 1W1W) head structures.

SUPPLEMENTAL TABLES

Table S1. Recombinant DNA (related to Figures 1 to 6)

1771	pETMCN 6×HIS-TEV-Brn1$_{112–204}$-Smc2$_{hd}$ (res.112–204 of *Ct* Brn1 and res. 2–224, 981–1179 of *Ct* Smc2)
2858	pETMCN 6×HIS-TEV-Brn1$_{112–204}$-Smc2$_{hd\ EQ}$ (res. 112–204 of *Ct* Brn1 and res. 2–224, 981–1179 of *Ct* Smc2 with E1116Q)
3138	pETMCN 6×HIS-TEV-Brn1$_{112–204}$-Smc2$_{hd\ QL}$ (res. 112–204 of *Ct* Brn1 and res. 2–224, 981–1179 of *Ct* Smc2 with Q147L)
3388	pETMCN 6×HIS-TEV-Brn1$_{112–204}$-Smc2$_{hd\ SR}$ (res. 112–204 of *Ct* Brn1 and res. 2–224, 981–1179 of *Ct* Smc2 with S1088R)
2936	pETMCN 6×HIS-TEV-Brn1$_{112–204}$-Smc2$_{hd\ WA}$ (res. 112–204 of *Ct* Brn1 and res. 2–224, 981–1179 of *Ct* Smc2 with W1080A)
1911	pETMCN 6×HIS-TEV-Brn1$_{112–204}$-Smc2$_{hd\ II}$ (res. 112–204 of *Ct* Brn1 and res. 2–215, 990–1179 of *Ct* Smc2)
3427	pETMCN Brn1$_{112–204}$-Smc2$_{hd}$-6×HIS (res. 112–204 of *Ct* Brn1 and res. 2–224, 981–1179 of *Ct* Smc2)
3428	pETMCN Brn1$_{112–204}$-Smc2$_{hd\ TKK-AEE}$-6×HIS (res. 112–204 of *Ct* Brn1 and res. 2–224, 981–1179 of *Ct* Smc2 with T184A, K187E, K188E)
3426	pETMCN Brn1$_{112–204}$-Smc2$_{hd\ DK-AE}$-6×HIS (res. 112-204 of *Ct* Brn1 and res. 2–224, 981–1179 of *Ct* Smc2 with D1013A, K1016E)
3577	pETMCN Brn1$_{112–204,\ R183D}$-Smc2$_{hd}$-6×HIS (res. 112–204 of *Ct* Brn1 with R183D and res. 2–224, 981–1179 of *Ct* Smc2)
3578	pETMCN Brn1$_{112-204,\ Y180A}$-Smc2$_{hd}$-6×HIS (res. 112–204 of *Ct* Brn1 with Y180A and res. 2–224, 981–1179 of *Ct* Smc2)
3627	pETMCN Brn1$_{112-204,\ Y180F}$-Smc2$_{hd}$-6×HIS (res. 112–204 of *Ct* Brn1 with Y180F and res. 2–224, 981–1179 of *Ct* Smc2)
2676	pETMCN 6×HIS–Brn1$_{112-204}$–Smc2$_{cc}$ fusion (res. 112–204 of *Ct* Brn1 and res. 981–1031, 170–224 of *Ct* Smc2)
2566	Multibac cre pIDC Brn1$_{765–898}$-pFL Smc4$_{hd}$-8×HIS (res. 765–898 of *Ct* Brn1 and res. 264–466, 1367–1542 *Ct* Smc4)
2775	Multibac cre pIDC Brn1$_{765–898}$-pFL Smc4$_{hd\ EQ}$-8×HIS (res. 765–898 of *Ct* Brn1 and res. 264–466, 1367–1542 of *Ct* Smc4 with E1475Q)
3224	Multibac cre pIDC Brn1$_{765–898}$-pFL Smc4$_{hd\ QL}$-8×HIS (res. 765–898 of *Ct* Brn1 and res. 264–466, 1367–1542 of *Ct* Smc4with Q421L)
3580	Multibac cre pIDC Brn1$_{765–898}$-pFL Smc4$_{hd\ EQ,\ SR}$-8×HIS (res. 765–898 of *Ct* Brn1 and res. 264–466, 1367–1542 of *Ct* Smc4 with E1475Q, S1447R)
2944	Multibac cre pIDC Brn1$_{765–898}$-pFL Smc4$_{hd\ EQ,\ WA}$-8×HIS (res. 765–898 of *Ct* Brn1 and res. 264–466, 1367–1542 of *Ct* Smc4 with E1475Q, W1440A)
3405	Multibac cre pIDC Brn1$_{765–898}$-pFL Smc4$_{hd\ SR}$-8×HIS (res. 765–898 of *Ct* Brn1 and res. 264–466, 1367–1542 of *Ct* Smc4 with S1447R)
3406	Multibac cre pIDC Brn1$_{765–898}$-pFL Smc4$_{hd\ SD,\ WA}$-8×HIS (res. 765–898 of *Ct* Brn1 and res. 264–466, 1367–1542 of *Ct* Smc4 with S1439D, W1440A)
3404	Multibac cre pIDC Brn1$_{765–898}$-pFL Smc4$_{hd\ FA,\ RD}$-8×HIS (res. 765–898 of *Ct* Brn1 and res. 264–466, 1367–1542 of *Ct* Smc4 with F1482A, R1483D)
2943	Multibac cre pIDC Brn1$_{765–898}$-pFL Smc4$_{hd\ WA}$-8×HIS (res. 765–898 of *Ct* Brn1 and res. 264–466, 1367–1542 of *Ct* Smc4 with W1440A)
1896	pETMCN 6×HIS-TEV-Ycs4$_{3–1222}$ (res. 3–1222 of *Ct* Ycs4)
2319	pETMCN 6×HIS-TEV-Ycs4$_{3–827}$ (res. 3–827 of *Ct* Ycs4)
2322	pETMCN 6×HIS-Ycs4$_{3–689}$ (res. 3–689 of *Ct* Ycs4)
1857	pETMCN 6×HIS-Ycs4$_{3–518}$ (res. 3–518 of *Ct* Ycs4)
2221	pETMCN 6×HIS-Brn1$_{225–512}$-Ycs4 (res. 225–512 of *Ct* Brn1 and res. 3–1222 of *Ct* Ycs4)
2220	pETMCN 6×HIS-Brn1$_{336–512}$-Ycs4 (res. 336–512 of *Ct* Brn1 and res. 3–1222 of *Ct* Ycs4)
2777	pETMCN 6×HIS-Brn1$_{225–418}$-Ycs4$_{\Delta loops}$ (res. 225–418 of *Ct* Brn1 and res. 3–828, 869–915, 939–1222 of *Ct* Ycs4)
3176	pETMCN 6×HIS-Brn1$_{225–418}$-Ycs4$_{\Delta loops,\ KG\text{-}loop\ mut}$ (res. 225–418 of *Ct* Brn1 and res. 3–828, 869–915, 939–1222 of *Ct* Ycs4 with K1094D, V1095S, K1096D, Q1098D, L1099S)
1786	pGEX6PI GST-HRV3C-Brn1$_{225–340}$ (residues 225–340 of *Ct* Brn1)
1877	pGEX6PI GST-HRV3C-Brn1$_{225–512}$ (residues 225–512 of *Ct* Brn1)
1785	pGEX6PI GST-HRV3C-Brn1$_{336–512}$ (residues 336–512 of *Ct* Brn1)
1780	pGEX6PI GST-HRV3C-Brn1$_{336–714}$ (residues 336–714 of *Ct* Brn1)
1784	pGEX6PI GST-HRV3C-Brn1$_{513–714}$ (residues 513–714 of *Ct* Brn1)
1879	pGEX6PI GST-HRV3C-Brn1$_{636–714}$ (residues 636–714 of *Ct* Brn1)
2648	2µ pGAL7 Smc4-3×StrepII, pGAL10 Smc2, pGAL1 Brn1-12×HIS-3×HA, TRP1 (*Sc* Smc4, Smc2, Brn1)
3029	2µ pGAL7 Smc4$_{Q302L}$-3×StrepII, pGAL10 Smc2$_{Q147L}$, pGAL1 Brn1–12×HIS–3×HA, TRP1 (*Sc* Smc4 with Q302L, Smc2 with Q147L, Brn1)
2843	2µ pGAL1 Ycg1, pGAL10 Ycs4, URA3 (*Sc* Ycg1, Ycs4)
3308	2µ pGAL1 Ycg1, pGAL10 Ycs4$_{KG\text{-}loop\ mut}$, URA3 (*Sc* Ycg1, Ycs4 with K1049D, V1050S, K1051D, Q1053D, L1054S)
2845	2µ pGAL10 Ycs4, URA3 (*Sc* Ycs4)

Continued on next page

Table S1. Recombinant DNA (related to Figures 1 to 6) *continued from previous page*

3314	2μ pGAL7 Smc4-3×StrepII, pGAL10 Smc2, pGAL1 Brn1(ybbR$_{13–23}$, 3×TEV$_{141}$)-12×HIS-3×HA, TRP1 (*Sc* Smc4, Smc2, Brn1 with ybbR tag replacing res. 13–23, 3×TEV site inserted at res. 141)
3349	2μ pGAL7 Smc4$_{E1352Q}$-3×StrepII, pGAL10 Smc2$_{E1113Q}$, pGAL1 Brn1(ybbR$_{13–23}$, 3×TEV$_{141}$)-12×HIS-3×HA, TRP1 (*Sc* Smc4 with E1352Q, Smc2 with E1352Q, Brn1 with ybbR tag replacing res. 13–23, 3×TEV site inserted at res.141)
3371	2μ pGAL7 Smc4$_{Q302L}$-3×StrepII, pGAL10 Smc2$_{Q147L}$, pGAL1 Brn1(ybbR$_{13–23}$, 3×TEV$_{141}$)-12×HIS-3×HA, TRP1 (*Sc* Smc4 with Q302L, Smc2 with Q147L, Brn1 with ybbR tag replacing res. 13–23, 3×TEV site inserted at res. 141)
3378	2μ pGAL7 Smc4$_{S1324R}$-3×StrepII, pGAL10 Smc2$_{S1085R}$, pGAL1 Brn1(ybbR$_{13–23}$, 3×TEV$_{141}$)-12×HIS-3×HA, TRP1 (*Sc* Smc4 with S1324R, Smc2 with S1085R, Brn1 with ybbR tag replacing res. 13–23, 3×TEV site inserted at res. 141)
3373	2μ pGAL7 Smc4-3×StrepII, pGAL10 Smc2, pGAL1 Brn1(ybbR13–23, 1×TEV$_{141}$, 1×TEV$_{373}$)-12×HIS-3×HA, TRP1 (*Sc* Smc4, Smc2, Brn1 with ybbR tag replacing residues 13–23, 1×TEV sites inserted at res. 141 and 373)
1999	pSMC4-FLAG-EGFP (*Hs* SMC4)
1725	pSMC4-FLAG-EGFPWA (*Hs* SMC4 with W1185A)
1732	pSMC4-FLAG-EGFPSD, WA (*Hs* SMC4 with S1184D, W1185A)
1455	pFLAG-EGFP-NCAPD2 (*Hs* NCAPD2)
3649	pFLAG-EGFP-NCAPD2 (*Hs* NCAPD2 with K1384D, V1385S, K1386D, Q1388D, V1389S)
1288	6.4kb (plasmid backbone with *E. coli* ori and amp^{R})

Table S2. Uniprot Identifiers for Sequence Alignments (related to Figures 2, S1, S3, S5 and S7)

				Smc2	Smc4	Brn1	Ycs4
Animals	Vertebrates	Mammals	*Homo sapiens*	O95347	Q9NTJ3	Q15003	Q15021
			Mus musculus	Q8CG48	Q8CG47	Q8C156	Q8K2Z4
		Birds	*Gallus gallus*	Q90988	Q8AWB9	A0A1D5P3B2	A0A1L1RND5
		Reptiles	*Chelonia mydas*	M7AYY6	M7B6H5	M7BB33	M7BQD0
		Amphibians	*Xenopus laevis*	P50533	P50532	O13067	Q9YHY6
		Fish	*Danio rerio*	B8A5K9	E7FGC2	A1L231	B0V123
	Arthropods	Insects	*Drosophila melanogaster*	Q7KK96	Q9V3A7	P91663	Q9VAJ1
	Nematodes		*Trichinella spiralis*	A0A0V1B7P9	A0A0V1B923	E5SDR4	A0A0V1BS59
			Caenorhabditis elegans	Q09591	Q20060	G5EGE9	Q9U2M1
	Flatworms		*Schistosoma mansoni*	G4VP73 (gap)	G4LZF5	G4VCQ8	G4VHF7
Plants	Eudicots	Mustard family	*Arabidopsis thaliana*	Q9C5Y4	Q9FJL0	Q564K3	Q9M1J4
		Mallow family	*Theobroma cacao*	A0A061FZA3	A0A061EEP1	A0A061FCQ2	A0A061EVA8
		Myrtle family	*Eucalyptus grandis*	A0A059DKA1	A0A059AQS4	A0A059DDB4	A0A059DI45
		Pea family	*Glycine max*	I1M0W9	I1LWK5	I1KFG2	K7K8M4
		Grape family	*Vitis vinifera*	F6HAI6	D7SXB5	D7TEB5	F6HLQ7
		Nightshade family	*Solanum lycopersicum*	A0A097PJ88	K4BBV4	K4CF69	K4D2L4
	Monocots	Grass family	*Oryza sativa*	Q8GU55	Q8L6H8	B9EXC2	Q7XAM6
	Basal magnoliophyta	Amborella family	*Amborella trichopoda*	W1PPA4	W1PTT0	W1NZC1	U5CSJ8
	Mosses		*Physcomitrella patens*	A9S6L3	A9RDI2	A9TD22	A9TB92
	Red algae		*Galdieria sulphuraria*	M2XLH5	M2XMB5	M2XK80	M2WUI9
Fungi	Ascomycetes	Saccharomycetes	*Saccharomyces cerevisiae*	P38989	Q12267	P38170	Q06156
			Candida albicans	A0A1D8PI59	Q5A4Y2	A0A1D8PMC8	A0A1D8PI01
		Sordariomycetes	*Chaetomium thermophilum*	G0S5H7	G0S2G2	G0SBJ6	G0SB82
			Neurospora crassa	Q7S9M2	Q7S1T6	Q7SCS0	Q7S1I4
		Eurotiomycetes	*Emericella nidulans*	Q5B0N1	C8V7U2	Q5B5B5	C8VHB5
			Neosartorya fumigata	Q4X159	Q4WIE1	Q4WG60	A0A0J5ST09
		Dothideomycetes	*Botryosphaeria parva*	R1E6Z9	R1GIF9	R1GN90	R1GUT6
		Schizosaccharomycetes	*Schizosaccharomyces pombe*	P41003	P41004	Q9Y7R3	O94679
	Basidiomycetes		*Cryptococcus neoformans*	J9VUT8	J9VMA0	Q5K864	Q5K972
	Microsporidians		*Encephalitozoon cuniculi*	Q8SSJ9	Q8SRK4	Q8SWA2	Q8SSE2
Protists	Choanoflagellates		*Salpingoeca rosetta*	F2UFL3	F2U3W0	F2TXB9	F2UF62
	Amoebozoa	Dictyostelium	*Dictyostelium discoideum*	Q54PK4	Q54LV0	Q54DR4	Q54B17
	Alveolates	Ciliates	*Emericella nidulans*	Q22ST6	Q6PUA5	Q24BA4	Q233H3
	Stramenopiles	Diatoms	*Neosartorya fumigata*	B7GAL2	B5Y5J8	B7G0X0	B5Y4W4
			Thalassiosira pseudonana	B8BQT7	B8CCA2	B8BWW0	B8BQQ4
		Oomycetes	*Phytophthora infestans*	D0NY62	D0NXB1	D0MV02	D0P2X5
			Saprolegnia parasitica	A0A067CIY4	A0A067CB22	A0A067CV32	A0A067CDW8
	Cryptomonads		*Guillardia theta*	L1J804	L1JUU7	L1INS6	L1JQ47
	Euglenozoa	Kinetoplasts	*Trypanosoma brucei*	Q389U3	Q38CG6	Q57ZI0	Q38F58
			Leishmania major	Q4QJG2	Q4QC62	Q4QJ31	Q4QF22

Table S3. Yeast Genotypes (related to Figures 2, 4, 5, 6, S3, S6 and S7)

C4568	MATa/α, smc4::HIS3/SMC4, ura3::SMC4-HA$_6$::URA3/ura3
C4592	MATa/α, smc4::HIS3/SMC4, ura3::SMC4$_{S1316A}$-HA$_6$::URA3/ura3
C4595	MATa/α, smc4::HIS3/SMC4, ura3::SMC4$_{S1316D}$-HA$_6$::URA3/ura3
C4570	MATa/α, smc4::HIS3/SMC4, ura3::SMC4$_{W1317A}$-HA$_6$::URA3/ura3
C4590	MATa/α, smc4::HIS3/SMC4, ura3::SMC4$_{R1318E}$-HA$_6$::URA3/ura3
C4589	MATa/α, smc4::HIS3/SMC4, ura3::SMC4$_{S1324R}$-HA$_6$::URA3/ura3
C4564	MATa/α, smc2::hphMX4/SMC2, trp1::SMC2-PK$_6$::TRP1/trp1
C4567	MATa/α, smc2::hphMX4/SMC2, trp1::SMC2$_{W1077A}$-PK$_6$::TRP1/trp1
C4608	MATa/α, smc2::hphMX4/SMC2, trp1::SMC2$_{K1078E}$-PK$_6$::TRP1/trp1
C4582	MATa/α, smc2::hphMX4/SMC2, trp1::SMC2$_{S1085R}$-PK$_6$::TRP1/trp1
C4656	MATa, smc4::natMX, [YCplac111 SMC4-PK$_6$ LEU2], [pLH157::TRP1]
C4672	MATa, smc4::natMX, [YCplac111 SMC4$_{M1276amb}$-PK$_6$ LEU2], [pLH157 TRP1]
C4669	MATa, smc4::natMX, [YCplac111 SMC4$_{E1280amb}$-PK$_6$ LEU2], [pLH157 TRP1]
C4657	MATa, smc4::natMX, [YCplac111 SMC4$_{Q1283amb}$-PK$_6$ LEU2], [pLH157 TRP1]
C4670	MATa, smc4::natMX, [YCplac111 SMC4$_{M1284amb}$-PK$_6$ LEU2], [pLH157 TRP1]
C4671	MATa, smc4::natMX, [YCplac111 SMC4$_{M1287amb}$-PK$_6$ LEU2], [pLH157 TRP1]
C4673	MATa, smc4::natMX, [YCplac111 SMC4$_{V1296amb}$-PK$_6$ LEU2], [pLH157 TRP1]
C4681	MATa, smc4::natMX, [YCplac111 SMC4$_{S1298amb}$-PK$_6$ LEU2], [pLH157 TRP1]
C4715	MATa, smc4::natMX, YCS4-HA6::HIS3, [YCplac111 SMC4-PK$_6$ LEU2], [pLH157 TRP1]
C4697	MATa, smc4::natMX, YCS4-HA6::HIS3, [YCplac111 SMC4$_{M1276amb}$-PK$_6$ LEU2], [pLH157 TRP1]
C4700	MATa, smc4::natMX, YCS4-HA6::HIS3, [YCplac111 SMC4$_{Q1283amb}$-PK$_6$ LEU2], [pLH157 TRP1]
C4754	MATa, smc4::natMX, YCS4-HA6::HIS3, [YCplac111 SMC4$_{S1298amb}$-PK$_6$ LEU2], [pLH157 TRP1]
C4713	MATa, smc4::natMX, BRN1-HA6::HIS3, [YCplac111 SMC4-PK$_6$ LEU2], [pLH157 TRP1]
C4695	MATa, smc4::natMX, BRN1-HA6::HIS3, [YCplac111 SMC4$_{M1276amb}$-PK$_6$ LEU2], [pLH157 TRP1]
C4698	MATa, smc4::natMX, BRN1-HA6::HIS3, [YCplac111 SMC4$_{Q1283amb}$-PK$_6$ LEU2], [pLH157 TRP1]
C4752	MATa, smc4::natMX, BRN1-HA6::HIS3, [YCplac111 SMC4$_{S1298amb}$-PK$_6$ LEU2], [pLH157 TRP1]
C4714	MATa, smc4::natMX, YCG1-HA6::HIS3, [YCplac111 SMC4-PK$_6$ LEU2], [pLH157 TRP1]
C4696	MATa, smc4::natMX, YCG1-HA6::HIS3, [YCplac111 SMC4$_{M1276amb}$-PK$_6$ LEU2], [pLH157 TRP1]
C4699	MATa, smc4::natMX, YCG1-HA6::HIS3, [YCplac111 SMC4$_{Q1283amb}$-PK$_6$ LEU2], [pLH157 TRP1]
C4753	MATa, smc4::natMX, YCG1-HA6::HIS3, [YCplac111 SMC4$_{S1298amb}$-PK$_6$ LEU2], [pLH157 TRP1]
C5003	MATa/α, ycs4::kanMX6/YCS4, URA3/ura3
C5005	MATa/α, ycs4::kanMX6/YCS4, ura3::YCS4-PK$_6$::URA3/ura3
C5007	MATa/α, ycs4::kanMX6/YCS4, ura3::YCS4$_{K1048D, V1049S, K1050D, Q1052D, L1053S}$-PK$_6$::URA3/ura3
C5277	MATa/α, smc2::HIS3/SMC2, ura3::SMC2-PK$_6$::URA3/ura3
C5278	MATa/α, smc2::HIS3/SMC2, ura3::SMC2$_{T184A, K187E, K188E}$-PK$_6$::URA3/ura3
C5279	MATa/α, smc2::HIS3/SMC2, ura3::SMC2$_{D1010A, K1013E}$-PK$_6$::URA3/ura3
C4239	MATa/α, brn1::HIS3/BRN1, ura3::BRN1-PK$_6$::URA3/ura3
C5261	MATa/α, brn1::HIS3/BRN1, ura3::BRN1$_{Y86A}$-PK$_6$::URA3/ura3
C5262	MATa/α, brn1::HIS3/BRN1, ura3::BRN1$_{Y86F}$-PK$_6$::URA3/ura3
C5263	MATa/α, brn1::HIS3/BRN1, ura3::BRN1$_{R89D}$-PK$_6$::URA3/ura3

Continued on next page

Table S3. Yeast Genotypes (related to Figures 2, 4, 5, 6, S3, S6 and S7) *continued from previous page*

C4491	MATa, lys2::pGAL1 GAL4::LYS2, pep4::HIS3, bar1::hisG, [2µ, pGAL7 SMC4-(StrepII)$_3$, pGAL10 SMC2, pGAL1 BRN1-HA$_3$-His$_{12}$ TRP1], [2µ, pGAL1 YCG1, pGAL10 YCS4 URA3]
C4724	MATa, lys2::pGAL1 GAL4::LYS2, pep4::HIS3, bar1::hisG, [2µ, pGAL7 SMC4$_{Q302L}$-(StrepII)$_3$, pGAL10 SMC2$_{Q147L}$, pGAL1 BRN1-HA$_3$-His$_{12}$ TRP1], [2µ, pGAL1 YCG1, pGAL10 YCS4 URA3]
C5050	MATa, lys2::pGAL1 GAL4::LYS2, pep4::HIS3, bar1::hisG, [2µ, pGAL7 SMC4-(StrepII)$_3$, pGAL10 SMC2, pGAL1 BRN1-HA$_3$-His$_{12}$ TRP1], [2µ, pGAL1 YCG1, pGAL10 YCS4$_{K1048D, V1049S, K1050D, Q1052D, L1053S}$ URA3]
C4896	MATa, lys2::pGAL1 GAL4::LYS2, pep4::HIS3, bar1::hisG, [2µ, pGAL7 SMC4-(StrepII)$_3$, pGAL10 SMC2, pGAL1 BRN1(TEV141)$_3$-HA$_3$-His$_{12}$ TRP1], [2µ, pGAL1 YCG1, pGAL10 YCS4 URA3]
C5066	MATa, lys2::pGAL1 GAL4::LYS2, pep4::HIS3, bar1::hisG, [2µ, pGAL7 SMC4-(StrepII)$_3$, pGAL10 SMC2, pGAL1 ybbR(12-24)-BRN1(TEV141)$_3$-HA$_3$-His$_{12}$ TRP1], [2µ, pGAL1 YCG1, pGAL10 YCS4 URA3]
C5125	MATa, lys2::pGAL1 GAL4::LYS2, pep4::HIS3, bar1::hisG, [2µ, pGAL7 SMC4$_{Q302L}$-(StrepII)$_3$, pGAL10 SMC2$_{Q147L}$, pGAL1 ybbR$_{12–24}$-BRN1(TEV141)$_3$-HA3-His$_{12}$ TRP1], [2µ, pGAL10 YCS4,, pGAL1 YCG1 URA3]
C5139	MATa, lys2::pGAL1 GAL4::LYS2, pep4::HIS3, bar1::hisG, [2µ, pGAL7 SMC4$_{S1324R}$-(StrepII)$_3$, pGAL10 SMC2$_{S1085R}$, pGAL1 ybbR$_{12–24}$-BRN1(TEV141)$_3$-HA$_3$-His$_{12}$ TRP1], [2µ, pGAL10 YCS4, pGAL1 YCG1 URA3]
C5142	MATa, lys2::pGAL1 GAL4::LYS2, pep4::HIS3, bar1::hisG, [2µ, pGAL7 SMC4$_{E1352Q}$-(StrepII)$_3$, pGAL10 SMC2$_{E1113Q}$, pGAL1 ybbR$_{12–24}$-BRN1(TEV141)$_3$-HA$_3$-His$_{12}$ TRP1], [2µ, pGAL10 YCS4, pGAL1 YCG1 URA3]
C5110	MATa, lys2::pGAL1 GAL4::LYS2, pep4::HIS3, bar1::hisG, [2µ, pGAL7 SMC4-(StrepII)$_3$, pGAL10 SMC2, pGAL1 ybbR$_{12–24}$-BRN1(TEV141)3-HA$_3$-His$_{12}$ TRP1], [2µ, pGAL10 YCS4 URA3]
C5122	MATa, lys2::pGAL1 GAL4::LYS2, pep4::HIS3, bar1::hisG, [2µ, pGAL7 SMC4-(StrepII)$_3$, pGAL10-SMC2, pGAL1 ybbR$_{12–24}$-BRN1(TEV141, TEV373)-HA$_3$-His$_{12}$ TRP1], [2µ, pGAL10 YCS4, pGAL1 YCG1 URA3]

Table S4. Mass Spectrometry Data (related to Figure 2E)

Protein Name	Uniprot Identifier	Mass (kDa)	Score	Number Peptides	Sequence Coverage (%)
**Smc4$_{S1298bpa}$ –UV**					
None					
**Smc4$_{S1298bpa}$ +UV**					
Sc Smc4	Q12267	162.1	2,236	59	35.4
Sc Ycs4	Q06156	132.9	1,638	38	26.1
Sc Brn1	P38170	86.2	392	10	13.9
Sc Smc2	P38989	133.8	272	5	4.6

Table S5. NMR Statistics (related to Figure 5)

**Experimental restraints**	
Distance restraints	
Total NOEs (unambiguous/ambiguous)	2527/317
Short range (|i–j| ≤ 1)	1884/202
Medium range (|i–j| < 5)	265/66
Long range (|i–j| > 5)	378/49
Hydrogen bonds	61
Dihedral restraints (φ/ψ)	170/170
**Structural quality**	
Coordinate precision (Å, residues 58–80,130–160,167–193)	
Backbone (N, C_α, C')	0.65 ± 0.16
Heavy atoms	1.16 ± 0.15
Coordinate precision (Å, residues 16-46,51–56,58–80,130–160,167–193)	
Backbone (N, C_α, C')	0.79 ± 0.15
Heavy atoms	1.25 ± 0.15
Restraint RMSD	
Distance restraints (Å)	0.024 ± 0.005
Dihedral restraints (°)	0.91 ± 0.47
Deviation from idealized geometry	
Bond lengths (Å)	0.0032 ± 0.0001
Bond angles (°)	0.46 ± 0.02
**Ramachandran analysis (%)**	
Favoured regions	91.1 ± 1.4
Allowed regions	8.0 ± 1.5
Generously allowed	0.4 ± 0.3
Disallowed	0.6 ± 0.5
**Whatcheck analysis**	
1st generation packing	–0.717 ± 0.311
2nd generation packing	–2.019 ± 0.342
Ramachandran plot appearance	–1.689 ± 0.427
Chi^{-1}/Chi^{-2} rotamer normality	–2.958 ± 0.385
Backbone conformation	–0.666 ± 0.435
