# Supplementary material for: Structural Basis of an Asymmetric Condensin ATPase Cycle
Source: Mol Cell. 2019 Jun 20;74(6):1175–1188.e9. doi: 10.1016/j.molcel.2019.03.037 (PMC6591010; doi:10.1016/j.molcel.2019.03.037)
Supplement: Document S2. Article plus Supplemental Information [file mmc3.pdf]

Article

Molecular Cell

Structural Basis of an Asymmetric Condensin ATPase Cycle

Graphical Abstract

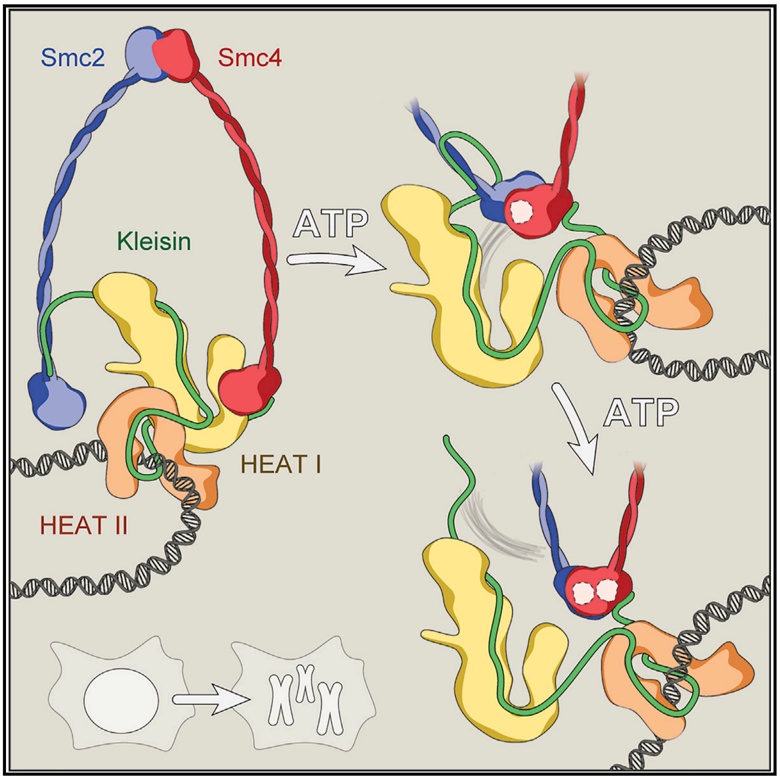

Highlights

- Smc4 and Smc2 ATPase head structures reorganize upon ATP binding and dimerization
- A Q-loop-mediated switch releases the Ycs4 HEAT-repeat subunit from the Smc4 head
- The Smc2 head engages with the ATP-bound Smc4 head into an asymmetric heterodimer
- Head dimerization releases the Brn1 kleisin from Smc2 via coiled-coil rotation

Authors

Markus Hassler, Indra A. Shaltiel, Marc Kschonsak, ..., Jutta Metz, Janosch Hennig, Christian H. Haering

Correspondence

christian.haering@embl.de

In Brief

Hassler et al. report structural and functional insights into the enzymatic core of the condensin protein complex that reveal large-scale conformational changes upon ATP binding by and subsequent dimerization of its catalytic SMC head domains. These movements presumably power the condensin-mediated extrusion of DNA loops during mitotic chromosome formation.

Hassler et al., 2019, Molecular Cell *74*, 1175–1188
June 20, 2019 © 2019 The Author(s). Published by Elsevier Inc.
https://doi.org/10.1016/j.molcel.2019.03.037

CellPress

Molecular Cell
Article

Structural Basis of an Asymmetric Condensin ATPase Cycle

Markus Hassler,[1] Indra A. Shaltiel,[1,4] Marc Kschonsak,[1,4,5] Bernd Simon,[2,4] Fabian Merkel,[1,3] Lena Thärichen,[1] Henry J. Bailey,[1,6] Jakub Macošek,[2] Sol Bravo,[1] Jutta Metz,[1,7] Janosch Hennig,[2] and Christian H. Haering[1,2,8,*]

[1]Cell Biology and Biophysics Unit, European Molecular Biology Laboratory, 69117 Heidelberg, Germany
[2]Structural and Computational Biology Unit, European Molecular Biology Laboratory, 69117 Heidelberg, Germany
[3]Collaboration for Joint PhD degree between EMBL and Heidelberg University, Faculty of Biosciences, Heidelberg, Germany
[4]These authors contributed equally
[5]Present address: Structural Biology, Genentech, South San Francisco, CA 94080, USA
[6]Present address: Structural Genomics Consortium, Nuffield Department of Medicine, University of Oxford, Oxford OX3 7DQ, UK
[7]Present address: Heidelberg University Biochemistry Center, 69120 Heidelberg, Germany
[8]Lead Contact
*Correspondence: christian.haering@embl.de
https://doi.org/10.1016/j.molcel.2019.03.037

SUMMARY

The condensin protein complex plays a key role in the structural organization of genomes. How the ATPase activity of its SMC subunits drives large-scale changes in chromosome topology has remained unknown. Here we reconstruct, at near-atomic resolution, the sequence of events that take place during the condensin ATPase cycle. We show that ATP binding induces a conformational switch in the Smc4 head domain that releases its hitherto undescribed interaction with the Ycs4 HEAT-repeat subunit and promotes its engagement with the Smc2 head into an asymmetric heterodimer. SMC head dimerization subsequently enables nucleotide binding at the second active site and disengages the Brn1 kleisin subunit from the Smc2 coiled coil to open the condensin ring. These large-scale transitions in the condensin architecture lay out a mechanistic path for its ability to extrude DNA helices into large loop structures.

INTRODUCTION

Multi-subunit protein complexes of the structural maintenance of chromosomes (SMC) family direct large-scale organizational changes in genome architecture that are essential for all aspects of chromosome biology. In addition to their central functions in the segregation of replicated genomes during prokaryotic and eukaryotic cell divisions (Hirano, 2016; Uhlmann, 2016), SMC protein complexes also determine the three-dimensional landscape of interphase nuclei to regulate gene expression (Albritton and Ercan, 2018; Merkenschlager and Nora, 2016) and contribute to DNA damage repair, recombination, and replication (Wood et al., 2010; Wu and Yu, 2012). The unifying principle for the diverse actions of SMC complexes, foremost condensin and cohesin, might be their ability to extrude DNA into loop structures (Goloborodko et al., 2016; Nasmyth, 2001). This hypothesis is consistent with the recent discoveries that condensin complexes purified from budding yeast are able to translocate along DNA double helices and processively expand loops of DNA of several kilobase pairs in length in a fashion that depends on their ability to hydrolyze ATP (Ganji et al., 2018; Terakawa et al., 2017). How the energy of nucleotide binding, hydrolysis, and release is converted into DNA translocation and looping movements has remained unknown, but currently available models rely on mechanochemical coupling of the ATPase cycle to large conformational transitions that affect chromosome interactions by creating topological compartments or contact sites that entrap or directly bind DNA, respectively (Gruber, 2017; Hassler et al., 2018).

Based on their homology to ATP binding cassette (ABC) transmembrane transporters and the Rad50 DNA damage repair protein (Hopfner, 2016), the two globular ATPase "head" domains situated at the ends of ~50-nm-long intra-molecular coiled coils of a heterodimer of condensin's Smc2 and Smc4 subunits are thought to sandwich a pair of ATP molecules between composite catalytic sites, each composed of Walker A (P loop) and Walker B motifs of one head and a so-called ABC signature motif of the opposite head. Each head domain (hd) can be subdivided into a "RecA"-like lobe that contains the ATP-binding pocket and a "helical" lobe that merges into the coiled coils; they connect the heads to a half-doughnut-shaped "hinge" dimerization domain. $Smc2_{hd}$ and $Smc4_{hd}$ are furthermore connected by their binding to opposite ends of the $Brn1^{Cnd2/NCAPH}$ kleisin subunit (Onn et al., 2007). Several crystal structures have revealed the formation of a helical bundle between the kleisin N-terminal domain and the coiled-coil "neck" region immediately adjacent to one SMC head (the ν-SMC_{hd}) and the interaction of the kleisin C-terminal winged helix domain (wHD) with the "cap" region located at the distal surface of the other SMC head (the κ-SMC_{hd}) of cohesin (Gligoris et al., 2014; Haering et al., 2004) or bacterial SMC complexes (Bürmann et al., 2013; Diebold-Durand et al., 2017; Kamada et al., 2017; Woo et al., 2009). No structural information has so far been available for the

This is an open access article under the CC BY license (http://creativecommons.org/licenses/by/4.0/).

homologous ATPase domains or interfaces of the condensin complex. The condensin SMC-kleisin ring structure has been proposed to topologically encircle chromosomal DNA (Cuylen et al., 2011), which is consistent with the finding that other tripartite SMC-kleisin rings that have been covalently circularized retain their association with chromosomal DNA even after protein denaturation (Gligoris et al., 2014; Haering et al., 2008; Wilhelm et al., 2015). Data from cohesin suggest that DNA release from these rings relies on the opening of the interface between the kleisin N terminus and the SMC coiled coil (cc), which serves as a DNA exit (Buheitel and Stemmann, 2013; Chan et al., 2012; Huis in 't Veld et al., 2014) and, potentially, also as an entry gate (Murayama and Uhlmann, 2015).

The central role of the ATPase cycle to SMC complex function is underscored by the fact that the majority of mutations that affect nucleotide binding, head dimerization, or ATP hydrolysis render cohesin or condensin non-functional (Arumugam et al., 2003; Hudson et al., 2008; Kinoshita et al., 2015; Palou et al., 2018; Thadani et al., 2018; Weitzer et al., 2003). Although these mutations largely abolish the association of cohesin with chromosomes, some of the homologous mutations in condensin have less dramatic effects on the chromosomal levels of condensin. This difference might be due to the presence of an ATPase-independent DNA binding site formed at the interface between the Brn1 kleisin subunit and the Ycg1$^{Cnd3/NCAPG}$ subunit, which is composed of multiple repeats of α-helical HEAT (huntingtin, elongation factor 3, protein phosphatase 2A, Tor1 kinase) motifs (Kschonsak et al., 2017). The function of the second HEAT-repeat subunit, named Ycs4$^{Cnd1/NCAPD2}$, has so far remained unclear, despite its presence being similarly essential for the association of condensin with chromosomes (Kinoshita et al., 2015; Lavoie et al., 2002).

Here we report high-resolution structures of the Smc2 and Smc4 heads, including their interfaces with the N- and C-terminal domains of the Brn1 kleisin subunit, and of the Ycs4-Brn1 complex. We provide evidence for rearrangements of key residues that take place during sequential ATP binding to the two catalytic sites and describe how these structural transitions trigger large-scale conformational changes that result in the dissociation of the Ycs4 subunit from a highly conserved binding site within the Smc4 head, Smc2-Smc4 head dimerization, and, ultimately, release of the Brn1 kleisin subunit from the Smc2 coiled coil.

RESULTS

Structural Basis for Asymmetric ATP Binding by the Condensin SMC Head Domains

To gain functional insights into the condensin ATPase cycle, we solved the crystal structures of Smc2$_{hd}$ and the Smc4$_{hd}$-Brn1$_C$ complex of the thermophilic yeast *Chaetomium thermophilum* (*Ct*; Figure S1A; Table S1) to 2.6- or 3.0-Å resolution, respectively (Figure 1A; Table 1). As expected, both structures revealed canonical two-lobed SMC ATPase folds that display a high degree of evolutionary surface conservation at their ATP-binding and head dimerization interfaces (Figure S1B). Although included in the crystallization construct, crystals of the *Ct* Smc2$_{hd}$ domain showed no electron density for the N-terminal Brn1 region, which presumably dissociated during crystallization (see below). In contrast, the *Ct* Smc4$_{hd}$ crystal structure displayed distinct density for the C-terminal Brn1 region, which folds into a wHD and binds to the cap' face of the SMC ATPase.

A comparison of the nucleotide-free *Ct* Smc2$_{hd}$ and Smc4$_{hd}$–Brn1$_C$ structures of condensin to the adenosine 5′-[γ-thio] triphosphate (ATPγS)-bound *Saccharomyces cerevisiae* (*Sc*) Smc1$_{hd}$-Scc1$_C$ and Smc3$_{hd}$-Scc1$_N$ structures of cohesin or to bacterial SMC ATPase head structures revealed differences in the orientations of the helical lobes and attached coiled coils relative to the RecA lobes, which can be explained by flexion movements of $\sim$25° or $\sim$15°, respectively (Figure 1B). These motions displace strictly conserved glutamine residues in the Smc2 and Smc4 Q loops, which are thought to coordinate the catalytic Mg^{2+} ion and contribute to nucleotide binding. Mutation of this residue in Smc4 to leucine indeed drastically reduced the low micromolar affinity of *Ct* Smc4$_{hd}$-Brn1$_C$ for ATP (Figure 1C). In contrast, even the wild-type (WT) version of *Ct* Smc2$_{hd}$-Brn1$_N$ was unable to bind ATP. This finding is readily explained by the *Ct* Smc2$_{hd}$ structure, where the more pronounced flexion of the Smc2 helical lobe not only repositions the Q loop but also induces a cascade of structural displacement events that alter the P loop of the ATP-binding pocket into a conformation that is incompatible with nucleotide binding (Figure 1B). This incompatibility with ATP binding is even more obvious in a second crystal form of *Ct* Smc2$_{hd}$ (Figure S1C).

Distinct Contributions of the Two ATPase Sites to SMC Head Dimerization

Despite these structural differences, we were able to trap a stable heterodimer of *Ct* Smc2$_{hd}$-Brn1$_N$ and *Ct* Smc4$_{hd}$-Brn1$_C$ when we prevented ATP hydrolysis by mutation of the catalytic Walker B glutamate residues in both heads (*Ct* Smc2$_{hd\ E1116Q}$, *Ct* Smc4$_{hd\ E1475Q}$; Figures 1D and S2A). This is consistent with the absence of discernible steric clashes in a structural model of an ATP-dimerized *Ct* Smc2$_{hd}$-Smc4$_{hd}$-Brn1$_C$ complex (Figure S2B). Consistent with the inability of *Ct* Smc2$_{hd}$ to bind ATP (Figure 1C), preventing ATP hydrolysis only at the Smc2 active site was insufficient for dimer formation, whereas mutation of only the Smc4 active site was sufficient (Figures 1D and S2A). Mutation of the Smc2 signature motif serine residue that contacts the nucleotide bound at the Smc4 active site (*Ct* Smc2$_{hd\ S1088R}$) prevented dimerization with Walker B mutant *Ct* Smc4$_{hd\ E1475Q}$, whereas simultaneous mutation of the Smc4 signature motif (*Ct* Smc4$_{hd\ S1447R,\ E1475Q}$) still allowed formation of a dimer that, however, eluted at a different retention volume during size-exclusion chromatography (Figure S2A). This suggests that a dimer with a distinct conformation can be mediated solely by ATP sandwiched between the Smc4 Walker A, Walker B, and Smc2 signature motifs. However, the second site formed by the Smc2 Walker A, Walker B, and Smc4 signature motifs must nevertheless be capable of binding and hydrolyzing ATP in the context of the heterodimer because mutation of the Smc2 Walker B motif had an even more severe effect on the basal ATPase activity of *Ct* Smc2$_{hd}$-Brn1$_N$ and *Ct* Smc4$_{hd}$-Brn1$_C$ complexes than mutation of the Smc4 Walker B motif (Figures S2C and S2D). ATP binding to the Smc4 active site is therefore sufficient to induce Smc2-Smc4 head dimerization, which then renders the Smc2 active site capable of binding and hydrolyzing ATP.

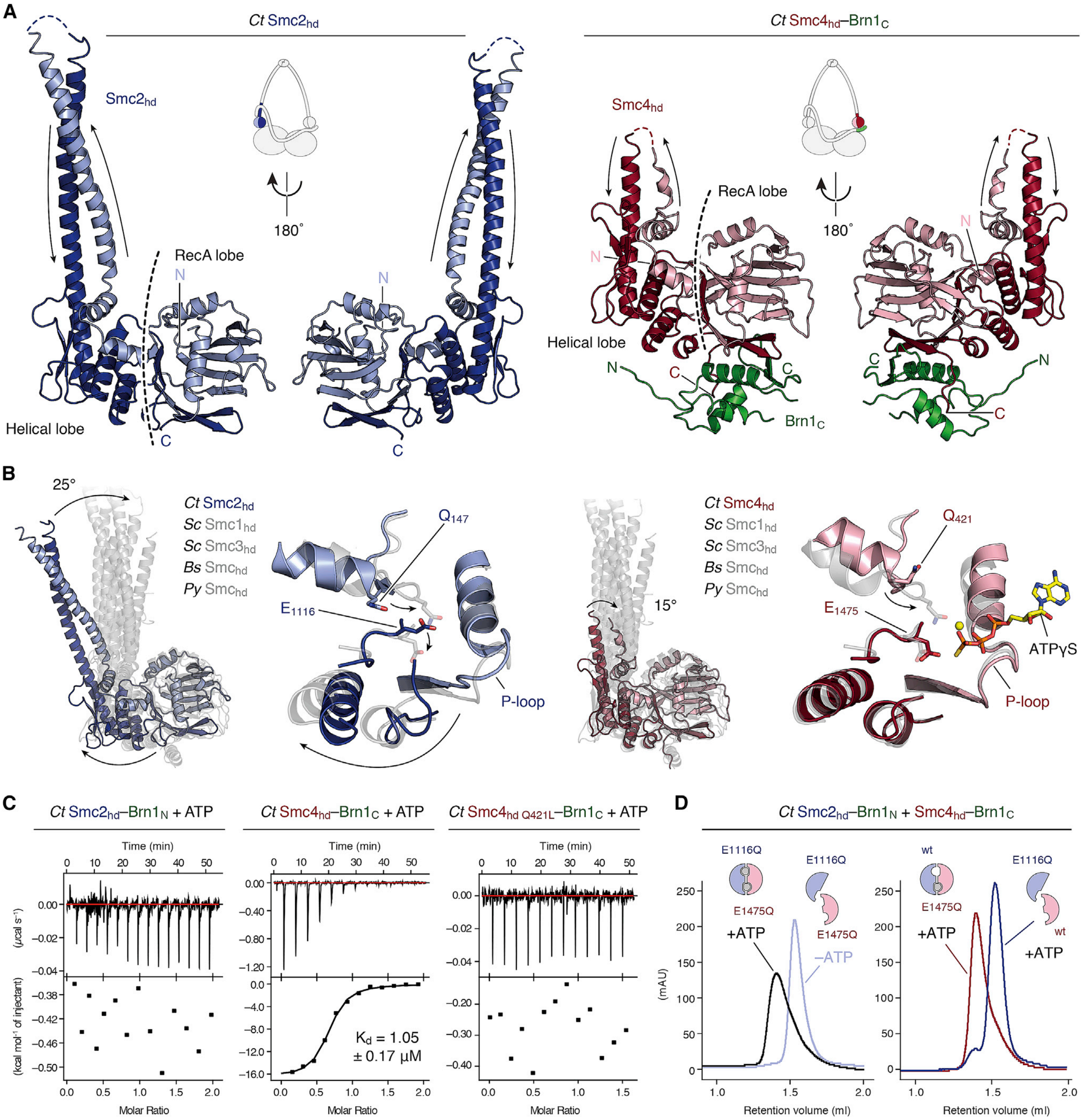

Figure 1. Structures and Dimerization of Smc2 and Smc4 ATPase Head Domains

(A) Cartoon models of the *Ct* Smc2$_{hd}$ (crystal form I) and the *Ct* Smc4$_{hd}$-Brn1$_{C}$ complex.

(B) Structural alignment based on the RecA-like lobe of *Ct* Smc2$_{hd}$ (I) and *Ct* Smc4$_{hd}$ structures to ATPγS-bound structures of the *Sc* cohesin Smc1$_{hd}$ (PDB: 1W1W; C$_{\alpha}$ root-mean-square deviation [RMSD] = 0.892 and 0.839) and Smc3$_{hd}$ (PDB: 4UX3; C$_{\alpha}$ RMSD = 2.582 and 1.068) or the nucleotide-free structures of *B. subtilis* (*Bs*) SMC$_{hd}$ (PDB: 3ZGX; C$_{\alpha}$ RMSD = 1.828 and 1.914) and *P. yayanosii* (*Py*) SMC$_{hd}$ (PDB: 5XEI; C$_{\alpha}$ RMSD = 0.977 and 0.818). Close-up views highlight the positions of the conserved Q-loop glutamine and Walker B glutamate residues aligned to *Sc* cohesin Smc1$_{hd}$ with ATPγS (gray).

(C) Isothermal titration calorimetry (ITC) of ATP binding by WT *Ct* Smc2$_{hd}$-Brn1$_{N}$ and WT or Q-loop mutant *Ct* Smc4$_{hd}$-Brn1$_{C}$ (fit ± error of the fit).

(D) Size exclusion chromatography profiles of double or single Walker B mutant combinations of *Ct* Smc2$_{hd}$-Brn1$_{N}$ and *Ct* Smc4$_{hd}$-Brn1$_{C}$ in the absence (–ATP) or presence (+ATP) of nucleotide.

See also Figures S1 and S2.

CellPress

Table 1. Crystallography Data Collection and Refinement Statistics

	Ct $Smc2_{hd}$ (I) (SeMet- SAD)	*Ct* Smc_{2hd} (II) (Native)	*Ct* $Smc4_{hd}$-$Brn1_{C}$	*Ct* Ycs4-$Brn1_{Y4}$ *(SeMet-SIRAS)**	*Ct* Ycs4-$Brn1_{Y4}$ (Native-SIRAS)	*Ct* Ycs4-$Brn1_{Y4}$-$Smc4_{hd}$-$Brn1_{C}$
Data collection						
Space group	P 2_1 2_1 2_1	P 6_5	P 6_4	P 2_1	P 2_1	P 2_1
Molecules per asymmetric unit	1	1	1	1	1	1
Cell dimensions (Å,°)						
a	47.32	93.75	132.72	86.61	86.07	84.40
b	107.98	93.75	132.72	81.76	80.79	82.38
c	174.25	117.97	75.55	132.88	130.84	177.83
α	90.00	90.00	90.00	90.00	90.00	90.00
β	90.00	90.00	90.00	93.15	93.40	98.77
γ	90.00	120.00	120.00	90.00	90.00	90.00
Resolution (Å)	45.94–2.50 (2.57–2.50)	81.19–2.00 (2.11–2.00)	45.74–2.90 (3.06–2.90)	45.11–3.30 (3.50–3.30)	44.63–3.38 (3.56–3.38)	47.74–5.50 (5.80–5.50)
R_{merge}	0.082 (1.854)	0.103 (1.733)	0.086 (1.497)	0.175 (1.108)	0.096 (0.763)	0.121 (1.359)
$I/\sigma I$	20.09 (1.50)	12.3 (1.40)	12.4 (1.10)	11.5 (2.1)	8.8 (1.7)	8.1 (0.9)
CC (½)	1.0 (0.515)	1.0 (0.558)	1.0 (0.497)	1.0 (0.704)	1.0 (0.456)	0.99 (0.358)
Completeness (%)	99.8 (97.6)	99.9 (99.2)	99.9 (99.7)	99.3 (96.1)	99.4 (99.5)	97.6 (99.6)
Redundancy	16.09 (15.64)	10.3 (10.5)	6.9 (7.1)	12.6 (7.2)	3.4 (3.4)	3.3 (3.5)
Refinement						
Resolution (Å)	45.90–2.56	66.88–2.00	43.45–3.00	45.11–3.30		47.74–5.80
No. reflections (total)	29,681	39,658	15,305	27,912		6,655
R_{work}/R_{free}	0.23/0.26	0.19/0.22	0.22/0.24	0.23/0.28		0.29/0.30
No. atoms						
Protein	3,205	3,178	3,474	7,704		10,778
Ligand or ion	0	0	30	0		0
Water	41	163	0	0		0
B-factors						
Protein	88.71	59.86	124.61	121.21		210.41
Ligand or ion	NA	NA	172.37	NA		NA
Water	74.31	56.64	NA	NA		NA
RMSDs						
Bond lengths (Å)	0.003	0.007	0.004	0.064		0.004
Bond angles (°)	0.629	0.855	0.771	1.220		0.692

Values in parentheses are for the highest-resolution shell; *from two merged datasets (used for refinement). NA, not available; SAD, single-wavelength anomalous diffraction; SeMEt, selenomethionine; SIRAS, single isomorphous replacement with anomalous scattering.

CellPress

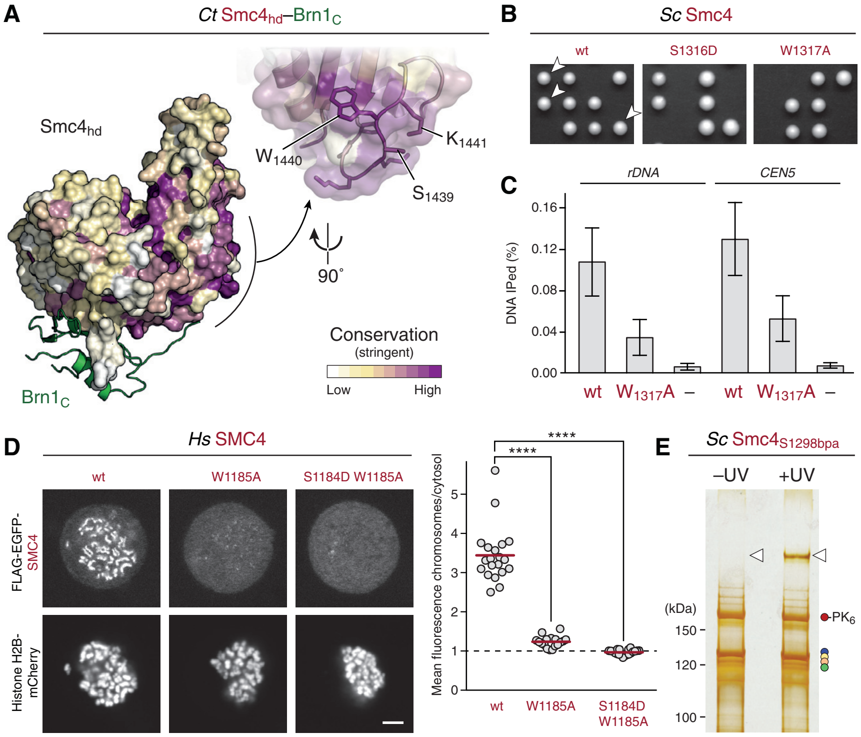

Figure 2. The Smc4 W-loop Is Essential for Condensin Function and Binds the Ycs4 Subunit

(A) Surface conservation of the *Ct* Smc4$_{hd}$-Brn1$_C$ structure with a close-up view of the W-loop region. Residues chosen for mutational analysis are indicated.

(B) Spores from diploid *Sc SMC4/smc4Δ* strains expressing an ectopic HA$_6$-tagged copy of WT Smc4 (strain C4568), Smc4$_{S1316D}$ (C4595), or Smc4$_{W1317A}$ (C4570) W-loop mutant versions were dissected and incubated for 3 days at 30°C.

(C) ChIP-qPCR at sites within the rDNA and at centromere V (*CEN5*) in diploid *Sc SMC4/smc4Δ* strains expressing no additional (C4936) or an ectopic HA$_6$-tagged copy of WT Smc4 (C4568) or Smc4$_{W1317A}$ (C4570) W-loop mutant versions (mean ± SD of 4 data points from 2 biological and 2 technical repeats each).

(D) Snapshots of live HeLa cells expressing mCherry-tagged histone H2B (bottom) and ectopic copies of FLAG-EGFP-tagged WT SMC4, SMC4$_{W1185A}$ single, or SMC4$_{S1184A,\ W1185A}$ double mutant versions (top). The graph shows the ratio of mean fluorescence EGFP signals in the chromosomal to cytosolic area for 20 data points (circles) and median (horizontal line) from 2 independent experiments (****$p < 0.0001$, Kolmogorov-Smirnov test).

(E) Silver stain of immunoprecipitated *Sc* condensin complexes (C4681) expressing a bpa-modified version as the only copy of Smc4 without (–UV) or after (+UV) crosslinking.

See also Figure S3.

A Conserved Patch on the Smc4 Head Binds to the Ycs4 HEAT-Repeat Subunit

A striking feature of the Smc4$_{hd}$ helical lobe is a highly conserved surface patch formed by residues within a loop that surround a strictly conserved tryptophan residue (W-loop; Figure 2A). The corresponding region in the homologous Smc1$_{hd}$ of cohesin also displays some degree of conservation, whereas the regions in the Smc2$_{hd}$ or Smc3$_{hd}$ structures show no obvious sequence conservation (Figure S3A; Table S2). Mutation to alanine of the strictly conserved tryptophan residue of Smc4 rendered budding yeast cells non-viable (*Sc* Smc4$_{W1317A}$), as did mutation to aspartate of the neighboring serine residue (*Sc* Smc4$_{S1316D}$; Figure 2B; Table S3). Mutation of the latter to alanine (*Sc* Smc4$_{S1316A}$), of the arginine residue following the tryptophan residue to aspartate (*Sc* Smc4$_{R1318E}$), or of the corresponding tryptophan or lysine residues in *Sc* Smc2 (*Sc* Smc2$_{W1077A}$, *Sc* Smc2$_{K1078E}$) had a less dramatic effect on cell proliferation (Figure S3B). None of these mutations affected Smc2 or Smc4 expression levels (Figure S3B). The loss of viability upon mutation of the Smc4 but not upon mutation of the Smc2 W-loop tryptophan residue can be explained by their differential effect on ATP hydrolysis rates because only the former (*Ct* Smc4$_{hd\ W1440A}$) but not the latter (*Ct* Smc2$_{hd\ W1080A}$) dramatically reduced ATP turnover by *Ct* Smc2$_{hd}$-Brn1$_N$ and *Ct* Smc4$_{hd}$-Brn1$_C$ complexes (Figures S2D and S2E). Because the heterodimer formed between *Ct* Smc2$_{hd}$-Brn1$_N$ and W-loop mutant Smc4$_{hd}$-Brn1$_C$ eluted during size-exclusion chromatography at the same retention volume as the dimer formed between *Ct* Smc2$_{hd}$-Brn1$_N$ and signature motif mutant Smc4$_{hd}$-Brn1$_C$ (Figure S2A), it is likely that the Smc4 W-loop mutation prevents the neighboring signature motif from sandwiching ATP bound to the Smc2 active site, which explains the reduction in ATPase rates. As an expected consequence of their inability to complete a full ATPase cycle, chromosome binding of yeast (Figure 2C) and human (Figure 2D) condensin complexes with Smc4 W-loop mutations was dramatically reduced when measured by chromatin immunoprecipitation followed by qPCR (ChIP-qPCR) or live-cell microscopy, respectively. We conclude that the conserved W-loop of Smc4 is essential for condensin's ATPase activity and for the stable association of condensin complexes with chromosomes.

To identify proteins that potentially interact with the W-loop surface patch of Smc4, we introduced the non-natural amino acid *p*-benzoyl-L-phenylalanine (bpa) into one of several positions surrounding the *Sc* Smc4 W-loop for *in vivo* photo-crosslinking (Chen et al., 2007). Three of seven different *Sc* Smc4$_{bpa}$ constructs produced upshifted bands in immunoblots for the PK$_6$ epitope tag fused to the C terminus of *Sc* Smc4 after UV crosslinking in live yeast cells (Figure S3C). Mass spectrometry of the upshifted band identified, in addition to *Sc* Smc4 itself, peptides of the *Sc* Ycs4 HEAT-repeat subunit and, to a lesser extent, the *Sc* Brn1 kleisin subunit (Figure 2E; Table S4). Western blotting against HA$_6$ epitope tags fused to the C termini of *Sc* Ycs4 or *Sc* Brn1 confirmed the presence of these subunits in

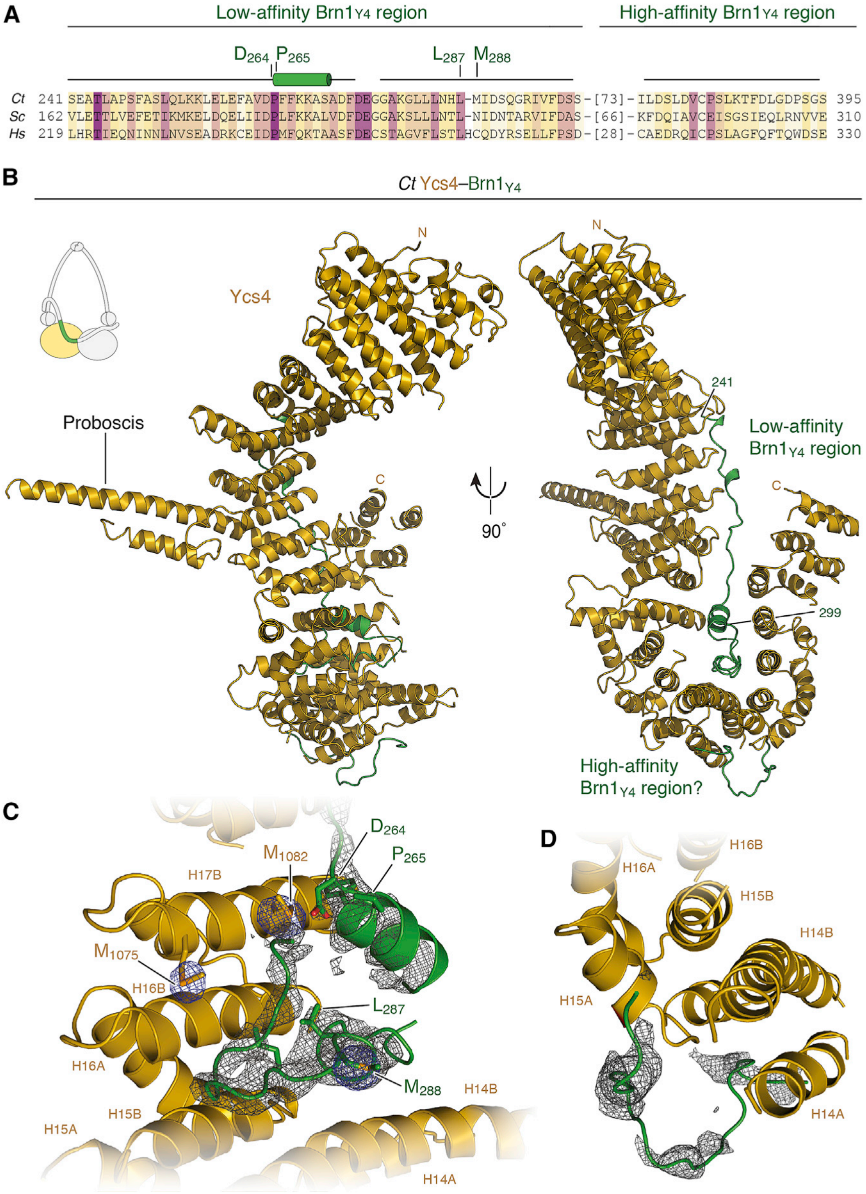

Figure 3. Structure of the Ycs4-Brn1$_{Y4}$ Complex

(A) Partial alignment of the extended Brn1$_{Y4}$ region that binds Ycs4. Colors indicate conservation scores calculated from an alignment of sequences from 40 species. Residues and secondary elements highlighted in the *Ct* Ycs4–Brn1$_{Y4}$ structure are indicated.

(B) Cartoon model of the 21 HEAT-repeat motifs of *Ct* Ycs4 (yellow) bound to residues 241 to 299 of *Ct* Brn1$_{Y4}$ (green). A helical insertion between Ycs4 HEAT-repeat motifs 8 and 9 creates an extended "trunk" (proboscis).

(C) Close-up view of Brn1$_{Y4}$ electron density in the Ycs4 U-turn. Anomalous difference density marking selenomethionine positions is shown in blue.

(D) Close-up view of additional electron density at the distal tip of the Ycs4 U-turn that presumably matches residues of the Brn1$_{Y4}$ high-affinity interaction region included in the crystallization construct.

See also Figures S4 and S5.

the upshifted bands, whereas the Ycg1 HEAT-repeat subunit did not shift under these conditions (Figure S3D).

Ycs4 Forms a Complex with Brn1 via Two Independent Binding Interfaces

Because Ycs4 has so far been thought to assemble into the condensin complex exclusively through its constitutive interaction with the Brn1 kleisin subunit (Onn et al., 2007; Piazza et al., 2014), we mapped the part of *Ct* Brn1 required for *Ct* Ycs4 binding to a high-affinity (K_d = 0.70 nM) core region of ~175 residues (*Ct* Brn1$_{336–512}$) and to an extended region that includes the preceding ~110 residues (*Ct* Brn1$_{225–512}$; Figures S4A and S4B). The additional segment of the extended region displays a higher degree of sequence conservation (Figures 3A and S4C) but lower affinity for *Ct* Ycs4 binding (Figure S4). Further truncation experiments showed that the N-terminal ~690 residues of *Ct* Ycs4 are sufficient for binding to the extended *Ct* Brn1 segment (Figure S4D) but with strongly reduced affinity (K_d = 1.22 μM) when compared to full-length *Ct* Ycs4 (Figure S4B). These results point to the presence of two distinct sites of contact between *Ct* Ycs4 and *Ct* Brn1: a high-affinity interface between the conserved C-terminal region of Ycs4 and the less conserved kleisin core binding region (*Ct* Brn1$_{336–512}$), and a low-affinity interface between the less conserved N-terminal part of Ycs4 and the more highly conserved extension of the kleisin core region (*Ct* Brn1$_{225–335}$).

Structure of the *Ct* Ycs4-Brn1 Complex

To reveal the interaction between the Brn1 kleisin and Ycs4 HEAT-repeat subunits at near-atomic resolution, we solved the co-crystal structure of *Ct* Ycs4 bound to *Ct* Brn1$_{225–418}$ to 3.3-Å resolution (*Ct* Ycs4-Brn1$_{Y4}$; Figure 3B; Table 1). The structure revealed a hook-shaped conformation of the 21 HEAT-repeat motifs of Ycs4 (Figure S5A), with the low-affinity binding region of the kleisin subunit (*Ct* Brn1$_{241–299}$) winding along the concave surface of the HEAT-repeat solenoid (Figure 3B) and entirely filling the space between the two lobes of the sharp U-turn in the C-terminal part of the Ycs4 subunit (Figure 3C). A marked drop in the quality of the electron density map in the second lobe because of crystallographic disorder only allowed modeling of a polyalanine chain into unaccounted electron density alongside the tip of the Ycs4 U-turn, which presumably corresponds to the high-affinity kleisin core binding region (Figure 3D). Localization of selenomethionine residues based on anomalous difference maps

and secondary structure prediction nevertheless allowed the assignment of residue numbers for almost all of Ycs4.

A search for similar structures in the PDB using the DALI server (Holm and Laakso, 2016) returned several HEAT-repeat proteins that fold into similarly curved shapes, including the cohesin subunits Pds5 and Scc3^{SA2} (Hara et al., 2014; Ouyang et al., 2016) and the condensin subunit Ycg1 (Kschonsak et al., 2017; Figure S5B). Comparison with the structure of the Scc2 subunit of the cohesin loader complex (Kikuchi et al., 2016) also revealed a strikingly similar overall shape despite the fact that this protein was not included in the list from the DALI server. A feature apparently unique to Ycs4 is an α-helical extension of HEAT-repeat motif 10 ("proboscis"; Figure 3B), which showed, however, no apparent primary sequence conservation among Ycs4 homologs (Figure S5A).

Structure of the *Ct* Ycs4-Brn1$_{Y4}$-Smc4$_{hd}$-Brn1$_C$ Complex

Consistent with the finding that the *Sc* Smc4 W-loop cross-links to *Sc* Ycs4 and *Sc* Brn1 *in vivo* (Figure 2E), *Ct* Ycs4-Brn1$_{Y4}$ and *Ct* Smc4$_{hd}$-Brn1$_C$ formed a stable stoichiometric complex *in vitro* (K_d = 0.63 μM; Figures 4A and S6A). We solved the co-crystal structure of this complex to 5.8-Å resolution (Table 1) and used the high-resolution structures of the individual sub-complexes (Figures 1A and 3B) and deformable elastic network refinement to build a coarse model of the *Ct* Ycs4-Brn1$_{Y4}$-Smc4$_{hd}$-Brn1$_C$ complex (Figure 4B). As expected, the Smc4 W-loop mediates the majority of interactions with the HEAT-repeat subunit and does so by contacting the second lobe of the Ycs4 U-turn. In addition, a strictly conserved phenylalanine-arginine residue pair adjacent to the so-called D-loop of the *Ct* Smc4 ATPase head contacts a helical extension of Ycs4 HEAT repeat 15. Despite the low resolution in the C-terminal half of Ycs4, we identified the highly conserved peptide loop that connects HEAT repeats 18 and 19 and contains a conserved lysine-glycine pair (KG-loop; Figure S5A) as the most likely candidate to mediate the interaction with the Smc4 W-loop by correlating sequence and structure based on the anomalous scattering of the selenomethionine residues and the prediction of secondary structure and HEAT-repeat organization (Figure 4B).

Mutation of the relevant residues in the Smc4 W- or D-loops or in the Ycs4 KG-loop sequence disrupted complex formation between *Ct* Ycs4-Brn1$_{Y4}$ and *Ct* Smc4$_{hd}$-Brn1$_C$ (*Ct* Smc4$_{hd\ SW\text{-}DA}$, *Ct* Smc4$_{hd\ FR\text{-}AE}$, or *Ct* Ycs4$_{KG\ loop\ mut}$, respectively; Figure S6A). Similar to mutation of the Smc4 W-loop, replacement of the Ycs4 KG-loop sequence rendered condensin non-functional in budding yeast (*Sc* Ycs4$_{KG\text{-}loop\ mut}$; Figure 4C) without affecting expression levels (Figure S6B). It furthermore severely compromised the basal and DNA-stimulated ATPase activities of condensin holocomplexes (Figures 4D and S6C) and strongly reduced condensin binding to chromosomes in budding yeast (Figure 4E) or mitotic human cells (*Homo sapiens* NCAPD2$_{KG\text{-}loop\ mut}$; Figure 4F). Like the Ycs4 KG-loop, the high-affinity core binding region of Brn1$_{Y4}$ is not visible in the structure because of crystallographic disorder. It nevertheless must contribute to the Smc4 interaction because its presence was essential for complex formation of *Ct* Ycs4-Brn1$_{Y4}$ with *Ct* Smc4$_{hd}$-Brn1$_C$ in pull-down assays (Figure S6D). We conclude that a contact between the Smc4 head and the Ycs4 HEAT-repeat subunit is mediated via contacts of Smc4 W- and D-loop residues with the Ycs4 KG-loop and the core region of Brn1$_{Y4}$. This contact is essential for condensin's abilities to complete a full ATPase cycle and to associate with chromosomes.

ATP-Dependent Control of the Ycs4-Smc4 Interaction

When we superimposed the model of the engaged *Ct* Smc2$_{hd}$-Smc4$_{hd}$-Brn1$_C$ ATPase head dimer (Figure S2B) onto the *Ct* Ycs4-Brn1$_{Y4}$-Smc4$_{hd}$-Brn1$_C$ structure, we noticed a pronounced steric clash between Smc2 and Ycs4 (Figure S6E). This implies that ATP-dependent Smc2-Smc4 head dimerization is incompatible with simultaneous binding of Ycs4-Brn1$_{Y4}$ to the Smc4 head. Consistent with this prediction, we found that addition of *Ct* Smc2$_{hd}$-Brn1$_N$ and ATP disrupted the interaction between *Ct* Ycs4-Brn1$_{Y4}$ and *Ct* Smc4$_{hd}$-Brn1$_C$ in pull-down assays (Figure S6F). Surprisingly, addition of ATP alone was similarly sufficient to compete with complex formation in this assay (Figure S6F) and during size-exclusion chromatography (Figures 4G and S6G). This effect was not caused by Smc4$_{hd}$ homodimerization in the presence of ATP, which has been observed for the homologous Smc1$_{hd}$ of cohesin (Haering et al., 2004), because ATP addition similarly disrupted Ycs4-Brn1$_{Y4}$ binding to a version of Smc4$_{hd}$-Brn1$_C$ that was unable to dimerize because of a mutation in the signature motif (*Ct* Smc4$_{hd\ S1447R}$; Figure S6G).

Addition of ADP, but not of AMP or guanosine triphosphate (GTP), prevented *Ct* Ycs4-Brn1$_{Y4}$ binding to *Ct* Smc4$_{hd}$-Brn1$_C$ with a similar efficiency as addition of ATP (Figure S6H). If ATP or ADP binding to the Smc4 RecA lobe induced dissociation of Ycs4-Brn1$_{Y4}$ from the helical lobe via a flexion movement of the two lobes (Figure 1B), then mutation of the central Q-loop should render the Ycs4-Brn1$_{Y4}$-Smc4$_{hd}$-Brn1$_C$ complex insensitive to nucleotide addition. This was indeed the case (*Ct* Smc4$_{hd\ Q421L}$; Figures 4G and S6G). To rule out that the continued association of the Smc4 Q-loop mutant complex in the presence of ATP was not merely due to the reduced affinity for nucleotide binding that results from a failure to coordinate Mg^{2+} at the active site (Figure 1C), we repeated the experiment under conditions (10 mM $MgCl_2$) that still allowed ATP binding by the Smc4$_{hd}$ Q-loop mutant with micromolar affinity (K_d = 18.35 μM; Figure S6I). Even under these conditions, ATP addition (1 mM) failed to release *Ct* Ycs4-Brn1$_{Y4}$ from *Ct* Smc4$_{hd}$-Brn1$_C$ (Figure S6G). These results strongly support a central role of the Q-loop motif in a conformational switch that transmits an allosteric change from the nucleotide-binding pocket of Smc4$_{hd}$ to its Ycs4-Brn1$_{Y4}$ W-loop interface.

The Smc2 Neck Region Binds Brn1

Because the Smc2$_{hd}$-Brn1$_N$ complex was refractory to crystallization, we determined an NMR structure of this dynamic interface by fusing the *Ct* Brn1$_N$ domain to the coiled-coil neck region of *Ct* Smc2 (Figures 5A and S7A; Table S5). Similar to the Smc3$_{hd}$-Scc1$_N$ interface in cohesin (Gligoris et al., 2014), the third "contact" helix (α_3) of the kleisin subunit forms an ~50-Å-long helical bundle with the SMC neck coiled coil (Figure S7B). This contact is stabilized by a salt bridge between highly conserved arginine and aspartate residues in the Brn1 contact

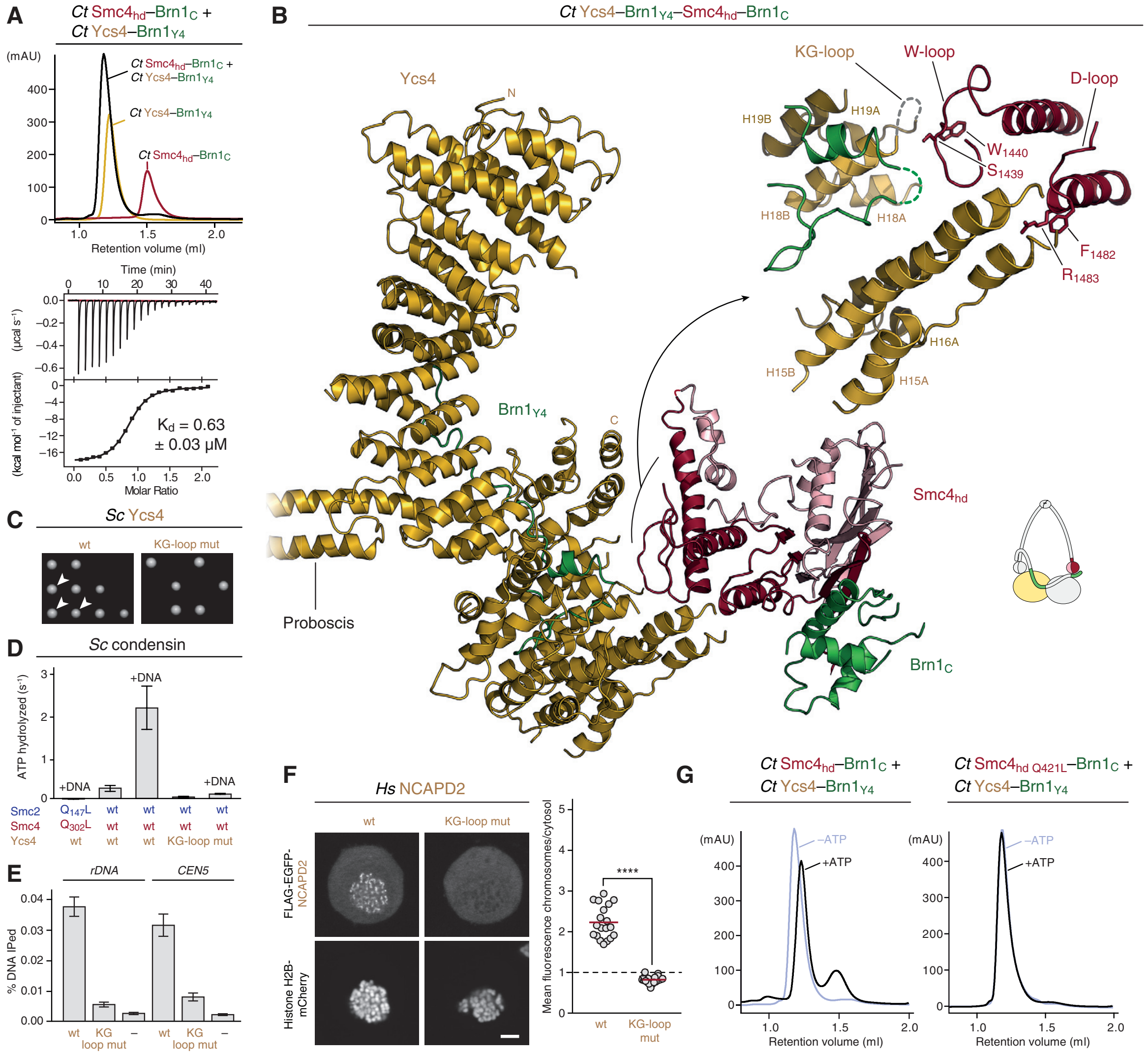

Figure 4. Structure and ATP-Dependent Release of the Ycs4-Smc4$_{hd}$ Interaction

(A) Size-exclusion chromatography of complexes formed between WT *Ct* Ycs4-Brn1$_{Y4}$ and *Ct* Smc4$_{hd}$-Brn1$_{C}$ and ITC of *Ct* Ycs4-Brn1$_{Y4}$ binding to *Ct* Smc4$_{hd}$-Brn1$_{C}$ (fit ± error of the fit).

(B) Cartoon model of the co-crystal structure of *Ct* Ycs4-Brn1$_{Y4}$ and *Ct* Smc4$_{hd}$-Brn1$_{C}$. A close-up view highlights contacts between the Smc4 W- and D-loops (red) with the Ycs4 KG-loop (yellow dotted line) and the helical extension of Ycs4 HEAT-repeat motif 15, respectively.

(C) Spores from diploid *Sc YCS4/ycs4Δ* strains expressing an ectopic PK$_6$-tagged copy of WT (strain C5005) or KVKGQL-DSDGDS KG-loop mutant (C5007) versions of Ycs4 were dissected and incubated for 3 days at 30°C.

(D) ATPase assays with WT (purified from strain C4491), Smc2 and Smc4 Q-loop (C4724), or Ycs4 KG-loop mutant (C5050) *Sc* condensin holocomplexes in the absence or presence of DNA (mean ± SD of 3 independent experiments).

(E) ChIP-qPCR at the rDNA and centromere V (*CEN5*) in diploid *Sc YCS4/ycs4Δ* strains expressing no (C5003) or an ectopic PK$_6$-tagged copy of WT (C5005) or KG-loop mutant (C5007) Ycs4 (mean ± SD of 4 data points from 2 biological and 2 technical repeats each).

(F) Snapshots of HeLa cells expressing mCherry-tagged histone H2B (bottom) and ectopic copies of FLAG-EGFP-tagged WT or KVKGQV-DSDGDS KG-loop mutant NCAPD2 (top). The graph shows the ratio of mean fluorescence EGFP signals in the chromosomal to cytosolic area for 20 data points (circles) and median (horizontal line) from 2 independent experiments (****$p < 0.0001$, Kolmogorov-Smirnov test).

(G) Size-exclusion chromatography of complexes formed between *Ct* Ycs4-Brn1$_{Y4}$ and WT (left) or Q-loop mutant (right) versions of *Ct* Smc4$_{hd}$-Brn1$_{C}$ in the absence (–ATP, light blue) or presence (+ATP, black) of ATP.

See also Figures S5 and S6.

helix and the C-terminal Smc2 neck helix, respectively (*Ct* Brn1$_{R183}$, *Ct* Smc2$_{D1013}$; Figures 5A and S7C). Mutations of these and neighboring residues rendered condensin nonfunctional in budding yeast (*Sc* Brn1$_{R89D}$ and *Ct* Smc2$_{DK\text{-}AE}$; Figures 5B and S7D) and disrupted the *Ct* Smc2$_{cc}$-Brn1$_{N}$ interaction (Figure 5C). Mutation of conserved residues in the N-terminal Smc2 neck helix that make no direct contact with Brn1 had, in contrast, no effect on the *Ct* Smc2$_{cc}$-Brn1$_{N}$ interaction (*Ct*

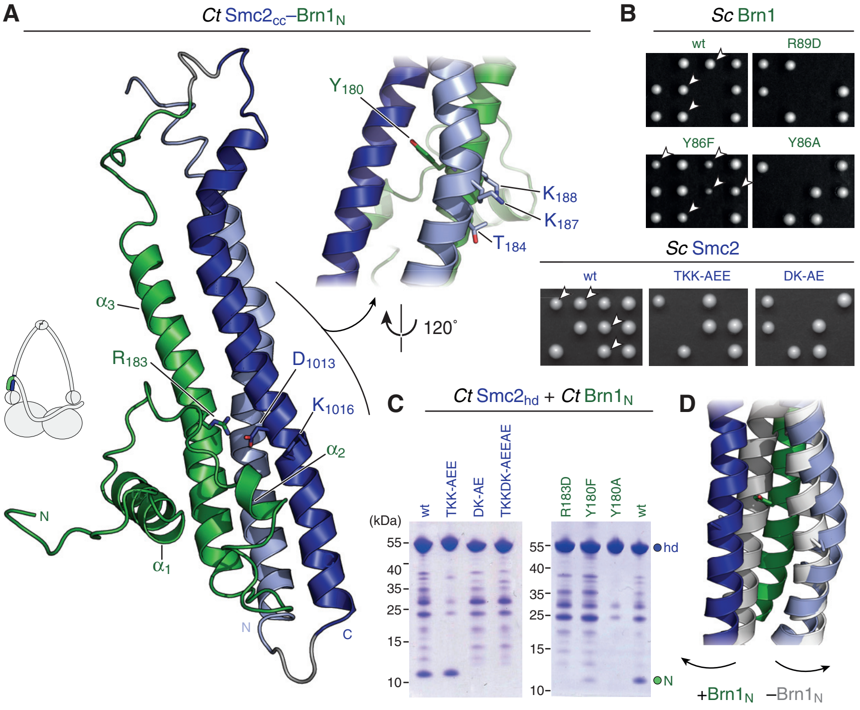

Figure 5. Structure of the Smc2$_{cc}$-Brn1$_N$ Interface

(A) Cartoon model of the lowest-energy *Ct* Smc2$_{cc}$-Brn1$_N$ NMR structure. Residues chosen for mutational analysis are highlighted. The close-up view shows *Ct* Brn1$_{Y180}$ intercalating into the Smc2 coiled coils and the positions of additional residues chosen for mutagenesis.

(B) Diploid *Sc BRN1/brn1Δ* strains expressing an ectopic PK$_6$-tagged copy of WT Brn1 (strain C5239) or Brn1$_{R89D}$ (C5263), Brn1$_{Y86F}$ (C5262) or Brn1$_{Y86A}$ (C5261) mutant versions and diploid *Sc SMC2/smc2Δ* strains expressing an ectopic PK$_6$-tagged copy of WT Smc2 (C5277), Smc2$_{TKK\text{-}AEE}$ (C5278), or Smc2$_{DK\text{-}AE}$ (C5279) mutant versions were dissected and incubated for 3 days at 30°C.

(C) Co-elution of His$_6$-tagged WT or mutant *Ct* Smc2$_{hd}$ constructs (left) and untagged WT or mutant *Ct* Brn1$_N$ constructs (right) from Ni-Sepharose beads tested by SDS-PAGE and Coomassie staining of elution fractions.

(D) Comparison of Smc2$_{cc}$ conformations in the *Ct* Smc2$_{cc}$-Brn1$_N$ NMR and *Ct* Smc2$_{hd}$ crystal structures. C$_\beta$ atoms of Smc2$_{A186}$ and Smc2$_{H1015}$, shown as sticks, serve as rotational markers.

See also Figure S7.

Smc2$_{TKK\text{-}AEE}$; Figure 5C) while nevertheless rendering condensin non-functional in yeast (Figure 5B). This suggests that the role of the Smc2 neck region might go beyond providing a binding platform for the kleisin subunit.

Comparison of the coiled-coil conformations in the *Ct* Smc2$_{hd}$ and *Ct* Smc2$_{cc}$-Brn1$_N$ structures implies that the formation of the three-helix bundle requires rotation of one helix by 60°–90° relative to the other helix and spreading of the two helices by 2.4 Å (Figure 5D). The deformation of the coiled coil is caused by the insertion of a conserved tyrosine residue located within the Brn1 contact helix (*Ct* Brn1$_{Y180}$; Figure 5A). A similar insertion of a conserved kleisin tyrosine residue between the coiled-coil helices can also be observed in the *Sc* Smc3$_{hd}$-Scc1$_N$ crystal structure of cohesin (*Sc* Scc1$_{Y83}$; Figure S7B), which suggests that this binding mode might be generally conserved among kleisin-SMC protein complexes. Mutation of this tyrosine residue to alanine eliminated binding to Smc2 *in vitro* (*Ct* Brn1$_{Y180A}$; Figure 5C) and condensin function *in vivo* (*Sc* Brn1$_{Y86A}$; Figures 5B and S7D), whereas mutation to a chemically similar phenylalanine residue had a more gradual effect on the Smc2$_{cc}$-Brn1$_N$ interaction (*Ct* Brn1$_{Y180F}$; Figure 5C) and cell proliferation (*Sc* Brn1$_{Y86F}$; Figures 5B and S7D).

ATP-Dependent Release of the Brn1 Kleisin from the Smc2 Coiled Coil

Spreading of the Smc2 coiled-coil helices to allow insertion of a tyrosine residue of the Brn1 contact helix provides a potential mechanism for allosteric control of this crucial interface in the condensin ring. We therefore purified *Sc* condensin holocomplexes in which we had inserted a triple repeat of the tobacco etch virus (TEV) protease cleavage site succeeding the Brn1 N-terminal domain (*Sc* Brn1$_{1–141}$) and followed the fate of this domain after TEV cleavage. In the absence of nucleotide, the majority of Brn1$_N$ remained bound to immobilized condensin (Figure 6A) or co-eluted with the complex during size-exclusion chromatography (Figure S7E). ATP addition released most (~95%) of the Brn1$_N$ cleavage fragments from condensin, even when nucleotide hydrolysis was prevented by Walker B mutations (*Sc* Smc2$_{E1113Q}$, *Sc* Smc4$_{E1352Q}$; Figures 6A and S7E). In contrast, mutations in the Q-loop motifs or in the signature motifs that prevent head dimerization rendered the Smc2-Brn1$_N$ interaction insensitive to ATP addition (*Sc* Smc2$_{Q147L}$, *Sc* Smc4$_{Q302L}$ or *Sc* Smc2$_{S1085R}$, *Sc* Smc4$_{S1323R}$, respectively; Figure 6A). ATP-dependent Brn1$_N$ dissociation was not affected by the absence of the Ycg1 HEAT-repeat subunit (Figure 6B) or release of the Ycs4-Brn1$_{Y4}$ subcomplex prior to nucleotide addition (Figure 6C). We conclude that ATP-dependent Smc2-Smc4 head dimerization following nucleotide binding induces condensin ring opening at the Smc2-Brn1 interface independent of the presence of either HEAT-repeat subunit.

DISCUSSION

Based on the current work, we propose a multi-step model of the condensin ATPase cycle (Figure 7). In the nucleotide-free state, only the Smc4 head is able to bind an ATP molecule (Figure 1C). Correlation of the nucleotide-free Smc4$_{hd}$-Brn1$_C$ structure with the ATPγS-bound structure of the homologous Smc1$_{hd}$-Scc1$_C$ complex (Haering et al., 2004) suggests that, upon ATP binding to the P loop of the Smc4 RecA lobe, the Q-loop glutamine residue repositions to form hydrogen bonds with the Mg^{2+} ion and the γ-phosphate (Figure 1B). As a consequence, the helical lobe tilts, relative to the RecA lobe, by 15° degrees (Video S1), which is similar to the flexion recently described for bacterial

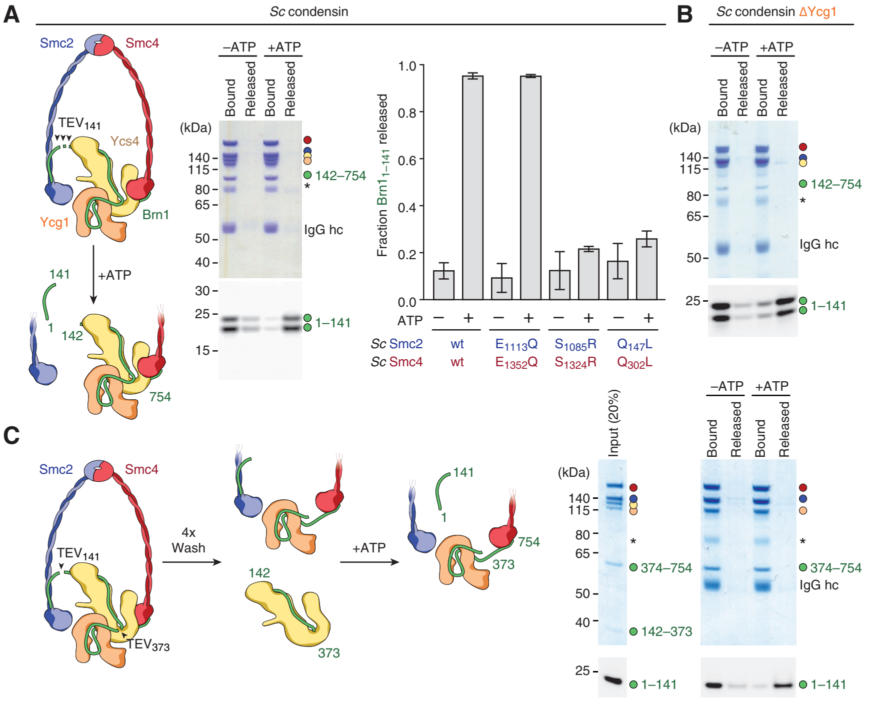

Figure 6. ATP-Binding-Dependent Release of the $Smc2_{cc}$-$Brn1_N$ Interaction

(A) *Sc* condensin holocomplexes containing a Brn1 subunit labeled via an N-terminal ybbR tag with the fluorescent dye ATTO488 and a triple TEV protease cleavage site at position 141 were incubated with TEV protease before immunoprecipitation. Shown is Coomassie staining (top) and an in-gel fluorescence scan (bottom) of SDS-PAGE of bound or released fractions after washing immunoprecipitation beads with buffer without nucleotide (–ATP) or containing 1 mM ATP (+ATP). The graph shows fluorescence intensities of the $Brn1_{1–141}$ fragment in the released fractions of WT (purified from strain C5066), Walker B ($Smc2_{E1113Q}$, $Smc4_{E1352Q}$; C5142), signature motif ($Smc2_{S1085R}$, $Smc4_{S1324R}$; C5139), or Q-loop ($Smc2_{Q147L}$, $Smc4_{Q302L}$; C5125) mutant condensin complexes (mean ± SD from 3 independent experiments).

(B) As in (A), using purified condensin complexes that lack the Ycg1 subunit (C5110).

(C) Brn1 of condensin holocomplexes (C5122) was cleaved simultaneously at positions 141 and 373. Extensive washing steps during immunoprecipitation removed Ycs4-$Brn1_{142–373}$. Immunoprecipitated condensin complexes were then washed with buffer without nucleotide (–ATP) or containing 1 mM ATP (+ATP) as in (A).

See also Figure S7.

SMC proteins (Kamada et al., 2017). The comparison of the $Smc4_{hd}$-$Brn1_C$ and $Smc1_{hd}$-$Scc1_C$ structures furthermore reveals an inversion of the side-chain conformations of a conserved tyrosine-(lysine or arginine) pair in a region of the helical lobe that we define as the W-loop (Figure S7F). It is likely that tilting and repositioning of W-loop residues dissociate Ycs4 (Figure 4G), which binds to this part of $Smc4_{hd}$ (Figure 4B) and sterically blocks access of $Smc2_{hd}$ (Figure S6E).

Ycs4, whose role for condensin function has so far remained incompletely understood, binds to two distinct sequence stretches of the Brn1 kleisin subunit (Figure S4): the N-terminal, low-affinity binding stretch winds along the inner surface of the hook-shaped HEAT-repeat solenoid (Figure 3C), whereas the high-affinity core binding region stretch is not clearly resolved in the crystal structure, although it is essential for the interaction of the Ycs4-$Brn1_{Y4}$ subcomplex with $Smc4_{hd}$ (Figure S6D). It is conceivable that contacts with this part of the kleisin subunit support the essential contacts between the Ycs4 KG-loop and the Smc4 W-loop and between the helical extension of Ycs4 HEAT-repeat motif 15 and the conserved phenylalanine-arginine residue pair at the tip of the Smc4 D-loop. Notably, the conservation of the KG-loop sequence extends to the NCAPD3 HEAT-repeat subunits of metazoan condensin II complexes (Figure S5A) and the identity of several residues in the loop segment of $Smc1_{hd}$ that corresponds to the $Smc4_{hd}$ W-loop has been maintained throughout evolution (Figure S3A). These findings raise the possibility that the interaction of a HEAT-repeat subunit with the κ-SMC_{hd} is a central feature of all condensin and cohesin complexes. In cohesin, the most likely HEAT-repeat subunit to bind $Smc1_{hd}$ might be Pds5, which is most similar in structure to Ycs4 (Figure S5B).

Smc2-Smc4 head dimerization commences in an asymmetric state in which only the Smc4 ATP-binding pocket is occupied (Figure 7). The Smc2 head fails to bind ATP on its own (Figure 1C), presumably because of the deformation of the Smc2 P loop observed in two different crystal forms (Figures 1B and S1C), but readily binds and hydrolyses ATP when in complex with the Smc4 head (Figure S2C). This transition is presumably the consequence of a pronounced reorientation of the Smc2 helical lobe and coiled coil by ~25° upon head dimerization, which is transmitted via D-loop and adjacent helices to create a P loop conformation that is compatible with nucleotide binding (Figure 1B). The subsequent sandwiching of the second ATP by the Smc4 signature motif presumably also depends on the prior reorientation of the neighboring W-loop because mutation of either of the two motifs results in head dimers with distinct elution profiles during size-exclusion chromatography (Figure S2A).

Our data furthermore suggest that, during formation of a pseudo-symmetric Smc2-Smc4 head dimer with both active sites occupied by ATP, changes in the Smc2 coiled-coil conformation markedly reduce the binding affinity to the Brn1 N terminus (Figure 6A). Because we observe Brn1 dissociation in the context of the full-length Smc2-Smc4 dimers but not for the $Smc2_{hd}$-$Brn1_N$ subcomplex (Figure S1A), the conformational transition of the coiled coil might require a pivot point, which could be situated in the "joint" region approximately one-third up the length of the coiled coil (Diebold-Durand et al., 2017) or generated by folding back of the SMC hinge domain onto the coiled coils (Bürmann et al., 2019). In either case, helix rotation provides a straightforward mechanism to control the propensity of the coiled coils to spread apart and accommodate insertion of the kleisin tyrosine side chain (Figure 5A). The fact that the

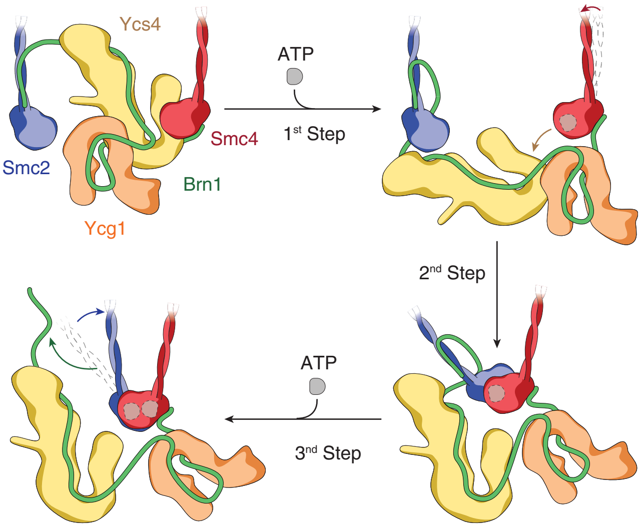

Figure 7. Model of the Condensin ATP-binding Cycle

Binding of ATP to the $Smc4_{hd}$ P loop site induces flexion of RecA and helical lobes that re-orient the coiled coils and W-loop regions and release the interaction with the Ycs4 subunit (step 1). Dissociation of Ycs4 allows $Smc4_{hd}$ engagement with $Smc2_{hd}$ via the bound ATP into an asymmetric dimer (step 2). This induces major flexion between the $Smc2_{hd}$ RecA and helical lobes that render the $Smc2_{hd}$ P loop capable of binding the second ATP molecule, resulting in a pseudo-symmetric $Smc2_{hd}$-$Smc4_{hd}$ dimer that sandwiches two ATP molecules between its catalytic sites (step 3). The simultaneous conformational change in the $Smc2_{hd}$ coiled coil releases $Brn1_N$ to open the condensin ring.

cohesin Scc1 kleisin subunit binds Smc3 in a similar fashion (Gligoris et al., 2014) and is also released upon ATP binding (Beckouët et al., 2016; Murayama and Uhlmann, 2015) strongly suggests that the reaction cycle we describe for condensin is fundamental to the action of all SMC protein complexes.

It seems reasonable to assume that the ATP-dependent conformational changes we describe provide the mechanistic basis for condensin to translocate along the DNA double helix and thereby extrude large DNA loops (Ganji et al., 2018; Terakawa et al., 2017), most likely by driving alternating DNA association and dissociation events. For example, binding of Ycs4 to the Smc4 head creates a topological compartment that might encircle DNA in a similar fashion as the Ycg1-Brn1 safety-belt compartment (Kschonsak et al., 2017). Because ADP, like ATP, is sufficient to dissociate Ycs4 from Smc4 (Figure S6H), it seems reasonable that such a compartment would only exist in a temporary nucleotide-free state. The structural transitions we report for the Smc2-Smc4 ATPase cycle would hence be reset not by hydrolysis, but only after nucleotide release, which is similar to many other ABC-type ATPases (Hopfner, 2016). Re-binding of Ycs4 to Smc4 at this point in the ATPase cycle might be an integral element because mutations that disrupt Ycs4 binding to Smc4 also affect ATPase rates (Figures 4D and S2E). The directionality that derives as a consequence of the strict asymmetry of this reaction cycle could resolve the conundrum how condensin is able to processively track along symmetric DNA molecules in one direction over distances of several kilobase pairs.

It is important to note that the asymmetry of condensin differs from that described for certain heterodimeric ABC transporters, which operate in an asymmetric fashion because only one of the two ATPase sites seems to be capable of hydrolyzing ATP (Procko et al., 2009). Furthermore, it has been suggested that many homodimeric ABC transporters use their two catalytic sites in an alternating fashion (Jones and George, 2013). Because even the two ATPase heads of bacterial SMC homodimers are embedded asymmetrically into the holocomplex by their binding to different ends of the kleisin subunit (Bürmann et al., 2013; Zawadzka et al., 2018), the asymmetric model put forward by our analysis of condensin might not only explain functional differences between the two ATPase sites of condensin's Smc2-Smc4 subunits or cohesin's Smc1-Smc3 subunits (Elbatsh et al., 2016) but, conceivably, apply to all SMC protein complexes. Future studies will need to complete the structural landscape of SMC holocomplexes to uncover how the asymmetric ATPase motor drives the diverse functions of this class of chromosome organizers.

STAR★METHODS

Detailed methods are provided in the online version of this paper and include the following:

- KEY RESOURCES TABLE
- CONTACT FOR REAGENT AND RESOURCE SHARING
- EXPERIMENTAL MODEL AND SUBJECT DETAILS
  - Cell Lines
  - Yeast Strains
  - Bacterial Strains
- METHOD DETAILS
  - Protein Expression and Purification
  - Crystallization and Data Collection
  - Structure Determination and Refinement
  - NMR spectroscopy and structure calculation
  - Multiple-sequence alignments
  - ATP Hydrolysis Assays
  - Isothermal Titration Calorimetry
  - Analytical Size-Exclusion Chromatography
  - GST Pulldown
  - Release of the amino-terminal Brn1fragment
  - Condensin Immunoprecipitation and Western Blotting
  - Bpa Crosslinking
  - Mass Spectrometry
  - ChIP-qPCR
  - Microscopy of Human Condensin Complexes
- QUANTIFICATION AND STATISTICAL ANALYSIS
- DATA AND SOFTWARE AVAILABILITY

SUPPLEMENTAL INFORMATION

Supplemental Information can be found online at https://doi.org/10.1016/j.molcel.2019.03.037.

ACKNOWLEDGMENTS

We thank D. Koppenhoefer, M. Marinova, and A. Landless for help with plasmid generation and protein purification; S. Winter for assisting with initial

NMR data analysis; K. Gull, K. Nasmyth, and J. Ellenberg for sharing plasmids, yeast strains, and antibodies; and C. Dekker, A. Elbatsh, T. Hirano, B. Rowland, and the members of the Haering lab for advice and discussions. We acknowledge support provided by B. Murciano of the EMBL Heidelberg Crystallization Platform, K. Perez of the Protein Expression and Purification Facility, the members of the EMBL Advanced Light Microscopy and Proteomics Core Facilities, and the staff of ESRF beamlines MASSIF-1, ID30-B, and ID29. This work has been funded by the European Molecular Biology Laboratory, the European Research Council (ERC-2015-CoG 681365 CondStruct to C.H.H.), and an NWO Rubicon fellowship (to I.A.S.).

AUTHOR CONTRIBUTIONS

M.H., M.K., S.B., L.T., and H.J.B. purified proteins and conducted biochemical assays. M.H., M.K., and L.T. crystallized the proteins. M.H. and M.K. collected data and solved the X-ray structures. B.S., J. Macošek, and J.H. solved the NMR structure. F.M. carried out imaging experiments with human cells. J. Metz, M.K., M.H., and C.H.H. conducted functional assays in yeast. J. Metz performed and analyzed ChIP-qPCR experiments. I.A.S. purified condensin holocomplexes and performed biochemical assays on the Smc2-Brn1$_N$ interface. M.H. and C.H.H. designed the study. J.H. and C.H.H. supervised the work. M.H. and C.H.H. wrote the manuscript with input from all authors.

DECLARATION OF INTERESTS

The authors declare no competing interests.

Received: December 13, 2018
Revised: March 16, 2019
Accepted: March 27, 2019
Published: June 20, 2019

REFERENCES

Adams, P.D., Afonine, P.V., Bunkóczi, G., Chen, V.B., Davis, I.W., Echols, N., Headd, J.J., Hung, L.W., Kapral, G.J., Grosse-Kunstleve, R.W., et al. (2010). PHENIX: a comprehensive Python-based system for macromolecular structure solution. Acta Crystallogr. D Biol. Crystallogr. *66*, 213–221.

Afonine, P.V., Grosse-Kunstleve, R.W., Echols, N., Headd, J.J., Moriarty, N.W., Mustyakimov, M., Terwilliger, T.C., Urzhumtsev, A., Zwart, P.H., and Adams, P.D. (2012). Towards automated crystallographic structure refinement with phenix.refine. Acta Crystallogr. D Biol. Crystallogr. *68*, 352–367.

Albritton, S.E., and Ercan, S. (2018). Caenorhabditis elegans Dosage Compensation: Insights into Condensin-Mediated Gene Regulation. Trends Genet. *34*, 41–53.

Arumugam, P., Gruber, S., Tanaka, K., Haering, C.H., Mechtler, K., and Nasmyth, K. (2003). ATP hydrolysis is required for cohesin's association with chromosomes. Curr. Biol. *13*, 1941–1953.

Ashkenazy, H., Abadi, S., Martz, E., Chay, O., Mayrose, I., Pupko, T., and Ben-Tal, N. (2016). ConSurf 2016: an improved methodology to estimate and visualize evolutionary conservation in macromolecules. Nucleic Acids Res. *44* (W1), W344-50.

Baker, N.A., Sept, D., Joseph, S., Holst, M.J., and McCammon, J.A. (2001). Electrostatics of nanosystems: application to microtubules and the ribosome. Proc. Natl. Acad. Sci. USA *98*, 10037–10041.

Beckouët, F., Srinivasan, M., Roig, M.B., Chan, K.L., Scheinost, J.C., Batty, P., Hu, B., Petela, N., Gligoris, T., Smith, A.C., et al. (2016). Releasing Activity Disengages Cohesin's Smc3/Scc1 Interface in a Process Blocked by Acetylation. Mol. Cell *61*, 563–574.

Bowler, M.W., Nurizzo, D., Barrett, R., Beteva, A., Bodin, M., Caserotto, H., Delagenière, S., Dobias, F., Flot, D., Giraud, T., et al. (2015). MASSIF-1: a beamline dedicated to the fully automatic characterization and data collection from crystals of biological macromolecules. J. Synchrotron Radiat. *22*, 1540–1547.

Brunger, A.T. (2007). Version 1.2 of the Crystallography and NMR system. Nat. Protoc. *2*, 2728–2733.

Brunger, A.T., Das, D., Deacon, A.M., Grant, J., Terwilliger, T.C., Read, R.J., Adams, P.D., Levitt, M., and Schröder, G.F. (2012). Application of DEN refinement and automated model building to a difficult case of molecular-replacement phasing: the structure of a putative succinyl-diaminopimelate desuccinylase from Corynebacterium glutamicum. Acta Crystallogr. D Biol. Crystallogr. *68*, 391–403.

Buheitel, J., and Stemmann, O. (2013). Prophase pathway-dependent removal of cohesin from human chromosomes requires opening of the Smc3-Scc1 gate. EMBO J. *32*, 666–676.

Bürmann, F., Shin, H.C., Basquin, J., Soh, Y.M., Giménez-Oya, V., Kim, Y.G., Oh, B.H., and Gruber, S. (2013). An asymmetric SMC-kleisin bridge in prokaryotic condensin. Nat. Struct. Mol. Biol. *20*, 371–379.

Bürmann, F., Lee, B.G., Than, T., Sinn, L., O'Reilly, F.J., Yatskevich, S., Rappsilber, J., Hu, B., Nasmyth, K., and Löwe, J. (2019). A folded conformation of MukBEF and cohesin. Nat. Struct. Mol. Biol. *26*, 227–236.

Chan, K.L., Roig, M.B., Hu, B., Beckouët, F., Metson, J., and Nasmyth, K. (2012). Cohesin's DNA exit gate is distinct from its entrance gate and is regulated by acetylation. Cell *150*, 961–974.

Chen, H.T., Warfield, L., and Hahn, S. (2007). The positions of TFIIF and TFIIE in the RNA polymerase II transcription preinitiation complex. Nat. Struct. Mol. Biol. *14*, 696–703.

Chen, V.B., Arendall, W.B., 3rd, Headd, J.J., Keedy, D.A., Immormino, R.M., Kapral, G.J., Murray, L.W., Richardson, J.S., and Richardson, D.C. (2010). MolProbity: all-atom structure validation for macromolecular crystallography. Acta Crystallogr. D Biol. Crystallogr. *66*, 12–21.

Cuylen, S., Metz, J., and Haering, C.H. (2011). Condensin structures chromosomal DNA through topological links. Nat. Struct. Mol. Biol. *18*, 894–901.

de Sanctis, D., Beteva, A., Caserotto, H., Dobias, F., Gabadinho, J., Giraud, T., Gobbo, A., Guijarro, M., Lentini, M., Lavault, B., et al. (2012). ID29: a high-intensity highly automated ESRF beamline for macromolecular crystallography experiments exploiting anomalous scattering. J. Synchrotron Radiat. *19*, 455–461.

Delaglio, F., Grzesiek, S., Vuister, G.W., Zhu, G., Pfeifer, J., and Bax, A. (1995). NMRPipe: a multidimensional spectral processing system based on UNIX pipes. J. Biomol. NMR *6*, 277–293.

Diebold-Durand, M.L., Lee, H., Ruiz Avila, L.B., Noh, H., Shin, H.C., Im, H., Bock, F.P., Burmann, F., Durand, A., Basfeld, A., et al. (2017). Structure of Full-Length SMC and Rearrangements Required for Chromosome Organization. Mol. Cell *67*, 334–347.e5.

Doublié, S. (1997). [29] Preparation of selenomethionyl proteins for phase determination. Methods Enzymol. *276*, 523–530.

Elbatsh, A.M.O., Haarhuis, J.H.I., Petela, N., Chapard, C., Fish, A., Celie, P.H., Stadnik, M., Ristic, D., Wyman, C., Medema, R.H., et al. (2016). Cohesin Releases DNA through Asymmetric ATPase-Driven Ring Opening. Mol. Cell *61*, 575–588.

Emsley, P., Lohkamp, B., Scott, W.G., and Cowtan, K. (2010). Features and development of Coot. Acta Crystallogr. D Biol. Crystallogr. *66*, 486–501.

Evans, P. (2006). Scaling and assessment of data quality. Acta Crystallogr. D Biol. Crystallogr. *62*, 72–82.

Evans, P.R. (2011). An introduction to data reduction: space-group determination, scaling and intensity statistics. Acta Crystallogr. D Biol. Crystallogr. *67*, 282–292.

Evans, P.R., and Murshudov, G.N. (2013). How good are my data and what is the resolution? Acta Crystallogr. D Biol. Crystallogr. *69*, 1204–1214.

Fitzgerald, D.J., Berger, P., Schaffitzel, C., Yamada, K., Richmond, T.J., and Berger, I. (2006). Protein complex expression by using multigene baculoviral vectors. Nat. Methods *3*, 1021–1032.

Franken, H., Mathieson, T., Childs, D., Sweetman, G.M., Werner, T., Tögel, I., Doce, C., Gade, S., Bantscheff, M., Drewes, G., et al. (2015). Thermal proteome profiling for unbiased identification of direct and indirect drug targets using multiplexed quantitative mass spectrometry. Nat. Protoc. *10*, 1567–1593.

CellPress

Ganji, M., Shaltiel, I.A., Bisht, S., Kim, E., Kalichava, A., Haering, C.H., and Dekker, C. (2018). Real-time imaging of DNA loop extrusion by condensin. Science *360*, 102–105.

Gligoris, T.G., Scheinost, J.C., Bürmann, F., Petela, N., Chan, K.L., Uluocak, P., Beckouët, F., Gruber, S., Nasmyth, K., and Löwe, J. (2014). Closing the cohesin ring: structure and function of its Smc3-kleisin interface. Science *346*, 963–967.

Goloborodko, A., Imakaev, M.V., Marko, J.F., and Mirny, L. (2016). Compaction and segregation of sister chromatids via active loop extrusion. eLife *5*, e14864.

Gruber, S. (2017). Shaping chromosomes by DNA capture and release: gating the SMC rings. Curr. Opin. Cell Biol. *46*, 87–93.

Haering, C.H., Schoffnegger, D., Nishino, T., Helmhart, W., Nasmyth, K., and Löwe, J. (2004). Structure and stability of cohesin's Smc1-kleisin interaction. Mol. Cell *15*, 951–964.

Haering, C.H., Farcas, A.M., Arumugam, P., Metson, J., and Nasmyth, K. (2008). The cohesin ring concatenates sister DNA molecules. Nature *454*, 297–301.

Hara, K., Zheng, G., Qu, Q., Liu, H., Ouyang, Z., Chen, Z., Tomchick, D.R., and Yu, H. (2014). Structure of cohesin subcomplex pinpoints direct shugoshin-Wapl antagonism in centromeric cohesion. Nat. Struct. Mol. Biol. *21*, 864–870.

Hassler, M., Shaltiel, I.A., and Haering, C.H. (2018). Towards a Unified Model of SMC Complex Function. Curr. Biol. *28*, R1266–R1281.

Hirano, T. (2016). Condensin-Based Chromosome Organization from Bacteria to Vertebrates. Cell *164*, 847–857.

Holm, L., and Laakso, L.M. (2016). Dali server update. Nucleic Acids Res. *44* (W1), W351-5.

Hopfner, K.P. (2016). Invited review: Architectures and mechanisms of ATP binding cassette proteins. Biopolymers *105*, 492–504.

Hudson, D.F., Ohta, S., Freisinger, T., Macisaac, F., Sennels, L., Alves, F., Lai, F., Kerr, A., Rappsilber, J., and Earnshaw, W.C. (2008). Molecular and genetic analysis of condensin function in vertebrate cells. Mol. Biol. Cell *19*, 3070–3079.

Huis in 't Veld, P.J., Herzog, F., Ladurner, R., Davidson, I.F., Piric, S., Kreidl, E., Bhaskara, V., Aebersold, R., and Peters, J.M. (2014). Characterization of a DNA exit gate in the human cohesin ring. Science *346*, 968–972.

Johnson, B.A., and Blevins, R.A. (1994). NMR View: A computer program for the visualization and analysis of NMR data. J. Biomol. NMR *4*, 603–614.

Jones, P.M., and George, A.M. (2013). Mechanism of the ABC transporter ATPase domains: catalytic models and the biochemical and biophysical record. Crit. Rev. Biochem. Mol. Biol. *48*, 39–50.

Kabsch, W. (2010). Xds. Acta Crystallogr. D Biol. Crystallogr. *66*, 125–132.

Kamada, K., Su'etsugu, M., Takada, H., Miyata, M., and Hirano, T. (2017). Overall Shapes of the SMC-ScpAB Complex Are Determined by Balance between Constraint and Relaxation of Its Structural Parts. Structure *25*, 603–616.e4.

Katoh, K., Misawa, K., Kuma, K., and Miyata, T. (2002). MAFFT: a novel method for rapid multiple sequence alignment based on fast Fourier transform. Nucleic Acids Res. *30*, 3059–3066.

Kikuchi, S., Borek, D.M., Otwinowski, Z., Tomchick, D.R., and Yu, H. (2016). Crystal structure of the cohesin loader Scc2 and insight into cohesinopathy. Proc. Natl. Acad. Sci. USA *113*, 12444–12449.

Kinoshita, K., Kobayashi, T.J., and Hirano, T. (2015). Balancing acts of two HEAT subunits of condensin I support dynamic assembly of chromosome axes. Dev. Cell *33*, 94–106.

Kschonsak, M., Merkel, F., Bisht, S., Metz, J., Rybin, V., Hassler, M., and Haering, C.H. (2017). Structural Basis for a Safety-Belt Mechanism That Anchors Condensin to Chromosomes. Cell *171*, 588–600.e24.

Larkin, M.A., Blackshields, G., Brown, N.P., Chenna, R., McGettigan, P.A., McWilliam, H., Valentin, F., Wallace, I.M., Wilm, A., Lopez, R., et al. (2007). Clustal W and Clustal X version 2.0. Bioinformatics *23*, 2947–2948.

Laskowski, R.A., Rullmannn, J.A., MacArthur, M.W., Kaptein, R., and Thornton, J.M. (1996). AQUA and PROCHECK-NMR: programs for checking the quality of protein structures solved by NMR. J. Biomol. NMR *8*, 477–486.

Lavoie, B.D., Hogan, E., and Koshland, D. (2002). In vivo dissection of the chromosome condensation machinery: reversibility of condensation distinguishes contributions of condensin and cohesin. J. Cell Biol. *156*, 805–815.

Linge, J.P., Habeck, M., Rieping, W., and Nilges, M. (2003). ARIA: automated NOE assignment and NMR structure calculation. Bioinformatics *19*, 315–316.

McCoy, A.J. (2007). Solving structures of protein complexes by molecular replacement with Phaser. Acta Crystallogr. D Biol. Crystallogr. *63*, 32–41.

Merkenschlager, M., and Nora, E.P. (2016). CTCF and Cohesin in Genome Folding and Transcriptional Gene Regulation. Annu. Rev. Genomics Hum. Genet. *17*, 17–43.

Mueller-Dieckmann, C., Bowler, M.W., Carpentier, P., Flot, D., McCarthy, A.A., Nanao, M.H., Nurizzo, D., Pernot, P., Popov, A., Round, A., et al. (2015). The status of the macromolecular crystallography beamlines at the European Synchrotron Radiation Facility. Eur. Phys. J. Plus *130*, 70.

Murayama, Y., and Uhlmann, F. (2015). DNA Entry into and Exit out of the Cohesin Ring by an Interlocking Gate Mechanism. Cell *163*, 1628–1640.

Nasmyth, K. (2001). Disseminating the genome: joining, resolving, and separating sister chromatids during mitosis and meiosis. Annu. Rev. Genet. *35*, 673–745.

Neumann, B., Walter, T., Hériché, J.K., Bulkescher, J., Erfle, H., Conrad, C., Rogers, P., Poser, I., Held, M., Liebel, U., et al. (2010). Phenotypic profiling of the human genome by time-lapse microscopy reveals cell division genes. Nature *464*, 721–727.

O'Donovan, D.J., Stokes-Rees, I., Nam, Y., Blacklow, S.C., Schröder, G.F., Brunger, A.T., and Sliz, P. (2012). A grid-enabled web service for low-resolution crystal structure refinement. Acta Crystallogr. D Biol. Crystallogr. *68*, 261–267.

Onn, I., Aono, N., Hirano, M., and Hirano, T. (2007). Reconstitution and subunit geometry of human condensin complexes. EMBO J. *26*, 1024–1034.

Ouyang, Z., Zheng, G., Tomchick, D.R., Luo, X., and Yu, H. (2016). Structural Basis and IP6 Requirement for Pds5-Dependent Cohesin Dynamics. Mol. Cell *62*, 248–259.

Palou, R., Dhanaraman, T., Marrakchi, R., Pascariu, M., Tyers, M., and D'Amours, D. (2018). Condensin ATPase motifs contribute differentially to the maintenance of chromosome morphology and genome stability. PLoS Biol. *16*, e2003980.

Pervushin, K., Riek, R., Wider, G., and Wüthrich, K. (1997). Attenuated T2 relaxation by mutual cancellation of dipole-dipole coupling and chemical shift anisotropy indicates an avenue to NMR structures of very large biological macromolecules in solution. Proc. Natl. Acad. Sci. USA *94*, 12366–12371.

Piazza, I., Rutkowska, A., Ori, A., Walczak, M., Metz, J., Pelechano, V., Beck, M., and Haering, C.H. (2014). Association of condensin with chromosomes depends on DNA binding by its HEAT-repeat subunits. Nat. Struct. Mol. Biol. *21*, 560–568.

Procko, E., O'Mara, M.L., Bennett, W.F., Tieleman, D.P., and Gaudet, R. (2009). The mechanism of ABC transporters: general lessons from structural and functional studies of an antigenic peptide transporter. FASEB J. *23*, 1287–1302.

Romier, C., Ben Jelloul, M., Albeck, S., Buchwald, G., Busso, D., Celie, P.H., Christodoulou, E., De Marco, V., van Gerwen, S., Knipscheer, P., et al. (2006). Co-expression of protein complexes in prokaryotic and eukaryotic hosts: experimental procedures, database tracking and case studies. Acta Crystallogr. D Biol. Crystallogr. *62*, 1232–1242.

Salzmann, M., Pervushin, K., Wider, G., Senn, H., and Wüthrich, K. (1998). TROSY in triple-resonance experiments: new perspectives for sequential NMR assignment of large proteins. Proc. Natl. Acad. Sci. USA *95*, 13585–13590.

Schindelin, J., Arganda-Carreras, I., Frise, E., Kaynig, V., Longair, M., Pietzsch, T., Preibisch, S., Rueden, C., Saalfeld, S., Schmid, B., et al. (2012). Fiji: an open-source platform for biological-image analysis. Nat. Methods *9*, 676–682.

Schröder, G.F., Levitt, M., and Brunger, A.T. (2010). Super-resolution biomolecular crystallography with low-resolution data. Nature *464*, 1218–1222.

Shen, Y., Delaglio, F., Cornilescu, G., and Bax, A. (2009). TALOS+: a hybrid method for predicting protein backbone torsion angles from NMR chemical shifts. J. Biomol. NMR *44*, 213–223.

Smith, D.B., and Johnson, K.S. (1988). Single-step purification of polypeptides expressed in Escherichia coli as fusions with glutathione S-transferase. Gene *67*, 31–40.

St-Pierre, J., Douziech, M., Bazile, F., Pascariu, M., Bonneil, E., Sauvé, V., Ratsima, H., and D'Amours, D. (2009). Polo kinase regulates mitotic chromosome condensation by hyperactivation of condensin DNA supercoiling activity. Mol. Cell *34*, 416–426.

Svensson, O., Malbet-Monaco, S., Popov, A., Nurizzo, D., and Bowler, M.W. (2015). Fully automatic characterization and data collection from crystals of biological macromolecules. Acta Crystallogr. D Biol. Crystallogr. *71*, 1757–1767.

Terakawa, T., Bisht, S., Eeftens, J.M., Dekker, C., Haering, C.H., and Greene, E.C. (2017). The condensin complex is a mechanochemical motor that translocates along DNA. Science *358*, 672–676.

Terwilliger, T.C., Grosse-Kunstleve, R.W., Afonine, P.V., Moriarty, N.W., Zwart, P.H., Hung, L.W., Read, R.J., and Adams, P.D. (2008). Iterative model building, structure refinement and density modification with the PHENIX AutoBuild wizard. Acta Crystallogr. D Biol. Crystallogr. *64*, 61–69.

Thadani, R., Kamenz, J., Heeger, S., Muñoz, S., and Uhlmann, F. (2018). Cell-Cycle Regulation of Dynamic Chromosome Association of the Condensin Complex. Cell Rep. *23*, 2308–2317.

Uhlmann, F. (2016). SMC complexes: from DNA to chromosomes. Nat. Rev. Mol. Cell Biol. *17*, 399–412.

Vonrhein, C., Blanc, E., Roversi, P., and Bricogne, G. (2007). Automated structure solution with autoSHARP. Methods Mol. Biol. *364*, 215–230.

Vriend, G. (1990). WHAT IF: a molecular modeling and drug design program. J. Mol. Graph. *8*, 52–56, 29.

Weitzer, S., Lehane, C., and Uhlmann, F. (2003). A model for ATP hydrolysis-dependent binding of cohesin to DNA. Curr. Biol. *13*, 1930–1940.

Wilhelm, L., Bürmann, F., Minnen, A., Shin, H.C., Toseland, C.P., Oh, B.H., and Gruber, S. (2015). SMC condensin entraps chromosomal DNA by an ATP hydrolysis dependent loading mechanism in Bacillus subtilis. eLife *4*.

Winn, M.D., Ballard, C.C., Cowtan, K.D., Dodson, E.J., Emsley, P., Evans, P.R., Keegan, R.M., Krissinel, E.B., Leslie, A.G., McCoy, A., et al. (2011). Overview of the CCP4 suite and current developments. Acta Crystallogr. D Biol. Crystallogr. *67*, 235–242.

Woo, J.S., Lim, J.H., Shin, H.C., Suh, M.K., Ku, B., Lee, K.H., Joo, K., Robinson, H., Lee, J., Park, S.Y., et al. (2009). Structural studies of a bacterial condensin complex reveal ATP-dependent disruption of intersubunit interactions. Cell *136*, 85–96.

Wood, A.J., Severson, A.F., and Meyer, B.J. (2010). Condensin and cohesin complexity: the expanding repertoire of functions. Nat. Rev. Genet. *11*, 391–404.

Woods, A., Sherwin, T., Sasse, R., MacRae, T.H., Baines, A.J., and Gull, K. (1989). Definition of individual components within the cytoskeleton of Trypanosoma brucei by a library of monoclonal antibodies. J. Cell Sci. *93*, 491–500.

Wu, N., and Yu, H. (2012). The Smc complexes in DNA damage response. Cell Biosci. *2*, 5.

Zawadzka, K., Zawadzki, P., Baker, R., Rajasekar, K.V., Wagner, F., Sherratt, D.J., and Arciszewska, L.K. (2018). MukB ATPases are regulated independently by the N- and C-terminal domains of MukF kleisin. eLife *7*, e31522.

STAR★METHODS

KEY RESOURCES TABLE

REAGENT OR RESOURCE	SOURCE	IDENTIFIER
**Antibodies**		
Mouse monoclonal anti V5-tag (anti PK6-tag)	AbD Serotec	Cat# MCA1360, RRID: AB_322378
Rabbit polyclonal anti HA-tag	Abcam	Cat# ab9110, RRID: AB_307019
Mouse monoclonal anti tubulin (TAT1)	Woods et al., 1989	N/A
Mouse monoclonal anti HA-tag (12CA5)	EMBL Protein Expression and Purification Core Facility	N/A
Rabbit polyclonal anti Sc Ycg1	Piazza et al., 2014	N/A
**Bacterial and Virus Strains**		
Escherichia coli Rosetta (DE3) pLysS	Merck	Cat# 70954
**Chemicals, Peptides, and Recombinant Proteins**		
C. thermophilum 6 × HIS–Brn1$_{112–204}$ in complex with Smc2$_{2–224/981–1179}$	This work	N/A
C. thermophilum 6 × HIS–Brn1$_{112–204}$ in complex with Smc2$_{2–224/981–1179,\ E1116Q}$	This work	N/A
C. thermophilum 6 × HIS–Brn1$_{112–204}$ in complex with Smc2$_{2–224/981–1179,\ Q147L}$	This work	N/A
C. thermophilum 6 × HIS–Brn1$_{112–204}$ in complex with Smc2$_{2–224/981–1179,\ S1088R}$	This work	N/A
C. thermophilum 6 × HIS–Brn1$_{112–204}$ in complex with Smc2$_{2–224/981–1179,\ W1080A}$	This work	N/A
C. thermophilum 6 × HIS–Brn1$_{112–204}$ in complex with Smc2$_{2–215/990–1179}$	This work	N/A
C. thermophilum Brn1$_{112–204}$ in complex with Smc2$_{2–224/981–1179}$–6 × HIS	This work	N/A
C. thermophilum Brn1$_{112–204}$ in complex with Smc2$_{2–224/981–1179,\ T184A,\ K187E,\ K188E}$–6 × HIS	This work	N/A
C. thermophilum Brn1$_{112–204}$ in complex with Smc2$_{2–224/981–1179,\ D1013A,\ K1016E}$–6 × HIS	This work	N/A
C. thermophilum Brn1$_{112-204,\ R183D}$ in complex with Smc2$_{2–224/981–1179}$–6 × HIS	This work	N/A
C. thermophilum Brn1$_{112-204,\ Y180A}$ in complex with Smc2$_{2–224/981–1179}$–6 × HIS	This work	N/A
C. thermophilum Brn1$_{112–204,\ Y180I}$ in complex with Smc2$_{2–224/981–1179}$–6 × HIS	This work	N/A
C. thermophilum Brn1$_{112-204,\ Y180F}$ in complex with Smc2$_{2–224/981–1179}$–6 × HIS	This work	N/A
C. thermophilum 6 × HIS–Brn1$_{112–204}$ fused to Smc2$_{981–1031/170–224}$	This work	N/A
C. thermophilum Brn1$_{765–898}$ in complex with Smc4$_{264–466/1367–1542}$–8 × HIS	This work	N/A
C. thermophilum Brn1$_{765–898}$ in complex with Smc4$_{264–466/1367–1542,\ E1475Q}$–8 × HIS	This work	N/A
C. thermophilum Brn1$_{765–898}$ in complex with Smc4$_{264–466/1367–1542,\ Q421L}$–8 × HIS	This work	N/A
C. thermophilum Brn1$_{765–898}$ in complex with Smc4$_{264–466/1367–1542,\ E1475Q,\ S1447R}$– 8 × HIS	This work	N/A

(*Continued on next page*)

Continued

REAGENT OR RESOURCE	SOURCE	IDENTIFIER
C. thermophilum $Brn1_{765–898}$ in complex with $Smc4_{264–466/1367–1542,\ E1475Q,\ W1440A}$–8 × HIS	This work	N/A
C. thermophilum $Brn1_{765–898}$ in complex with $Smc4_{264–466/1367–1542,\ S1447R}$–8 × HIS	This work	N/A
C. thermophilum $Brn1_{765–898}$ in complex with $Smc4_{264–466/1367–1542,\ S1439D,\ W1440A}$–8 × HIS	This work	N/A
C. thermophilum $Brn1_{765–898}$ in complex with $Smc4_{264–466/1367–1542,\ F1482A,\ R1483D}$–8 × HIS	This work	N/A
C. thermophilum $Brn1_{765–898}$ in complex with $Smc4_{264–466/1367–1542,\ W1440A}$–8 × HIS	This work	N/A
C. thermophilum 6 × HIS–$Ycs4_{3–1222}$	modified from Piazza et al., 2014	N/A
C. thermophilum 6 × HIS–$Ycs4_{3–827}$	modified from Piazza et al., 2014	N/A
C. thermophilum 6 × HIS–$Ycs4_{3–689}$	modified from Piazza et al., 2014	N/A
C. thermophilum 6 × HIS–$Ycs4_{3–518}$	modified from Piazza et al., 2014	N/A
C. thermophilum 6 × HIS–$Brn1_{225–512}$ in complex with $Ycs4_{3–1222}$	This work	N/A
C. thermophilum 6 × HIS–$Brn1_{336-512}$ in complex with $Ycs4_{3–1222}$	This work	N/A
C. thermophilum 6 × HIS–$Brn1_{225–512}$	This work	N/A
C. thermophilum 6 × HIS–$Brn1_{336–512}$	This work	N/A
C. thermophilum 6 × HIS–$Brn1_{225–418}$ in complex with $Ycs4_{3–828,\ 869–915,\ 939–1222}$	This work	N/A
C. thermophilum 6 × HIS–$Brn1_{225-418}$ in complex with $Ycs4_{3–828,\ 869–915,\ 939–1222,\ K1094D,\ V1095S,\ K1096D,\ Q1098D,\ L1099S}$	This work	N/A
C. thermophilum GST–$Brn1_{225–340}$	modified from Kschonsak et al., 2017	N/A
C. thermophilum GST $Brn1_{225–512}$	modified from Kschonsak et al., 2017	N/A
C. thermophilum GST $Brn1_{336–512}$	modified from Kschonsak et al., 2017	N/A
C. thermophilum GST $Brn1_{336–714}$	modified from Kschonsak et al., 2017	N/A
C. thermophilum GST $Brn1_{513–714}$	modified from Kschonsak et al., 2017	N/A
C. thermophilum GST $Brn1_{636–714}$	modified from Kschonsak et al., 2017	N/A
S. cerevisiae Smc2 in complex with Smc4–3 × StrepII, Brn1–12 × HIS–3 × HA, Ycg1, Ycs4	modified from Kschonsak et al., 2017	N/A
S. cerevisiae Smc2 in complex with Smc4–3 × StrepII, Brn1–12 × HIS–3 × HA, Ycg1, $Ycs4_{K1049D,\ V1050S,\ K1051D,\ Q1053D,\ L1054S}$	This work	N/A
S. cerevisiae $Smc2_{Q147L}$ in complex with $Smc4_{Q302L}$–3 × StrepII, Brn1–12 × HIS–3 × HA, Ycg1, Ycs4	This work	N/A
S. cerevisiae Smc2 in complex with Smc4–3 × StrepII, Brn1(ybbR tag replacing residues 13–23, 3 × TEV site inserted at residue 141)–12 × HIS–3 × HA, Ycg1, Ycs4	Ganji et al., 2018	N/A
S. cerevisiae Smc2 in complex with Smc4–3 × StrepII, Brn1(ybbR tag replacing residues 13–23, 3 × TEV site inserted at residue 141)–12 × HIS–3 × HA tag, Ycs4	This work	N/A
S. cerevisiae Smc2 in complex with Smc4–3 × StrepII, Brn1(ybbR tag replacing residues 13–23, 1 × TEV sites inserted at residues 141 and 373)– 12 × HIS-3 × HA, Ycg1, Ycs4	This work	N/A
S. cerevisiae $Smc2_{Q147L}$ in complex with $Smc4_{Q302L}$–3 × StrepII, Brn1(ybbR tag replacing residues 13–23, 3 × TEV site inserted at residue 141)–12 × HIS–3 × HA, Ycg1, Ycs4	This work	N/A

(*Continued on next page*)

Continued

REAGENT OR RESOURCE	SOURCE	IDENTIFIER
S. cerevisiae $Smc2_{S1085R}$ in complex with $Smc4_{S1324R}$–3 × StrepII, Brn1(ybbR tag replacing residues 13–23, 3 × TEV site inserted at residue 141)–12 × HIS–3 × HA, Ycg1, Ycs4	This work	N/A
S. cerevisiae $Smc2_{E1113Q}$ in complex with $Smc4_{E1352Q}$–3 × StrepII, Brn1(ybbR tag replacing residues 13–23, 3 × TEV-site inserted at residue 141)–12 × HIS–3 × HA, Ycg1, Ycs4	This work	N/A
Deposited Data		
C. thermophilum $Smc2_{hd}$ (I)	This work	PDB: 6QJ1
C. thermophilum $Smc2_{hd}$ (II)	This work	PDB: 6QJ0
C. thermophilum $Smc4_{hd}$–$Brn1_{C}$	This work	PDB: 6QJ2
C. thermophilum Ycs4–$Brn1_{Y4}$	This work	PDB: 6QJ3
C. thermophilum Ycs4–$Brn1_{Y4}$–$Smc4_{hd}$–$Brn1_{C}$	This work	PDB: 6QJ4
C. thermophilum $Smc2_{cc}$–$Brn1_{N}$	This work	PDB: 6Q6E
NMR chemical shifts and restraints	This work	BMRB: 34336
Original image files	This work	https://doi.org/10.17632/rk9hdmj8tk.1
Experimental Models: Cell Lines		
HeLa Kyoto H2B-mCherry	Neumann et al., 2010	N/A
Experimental Models: Organisms/Strains		
See Table S3		
Oligonucleotides		
rDNA 5′-TTTCTGCCTTTTTCGGTGAC-3′	Cuylen et al., 2011	Oligo SC41
rDNA 5′-TGGCATGGATTTCCCTTTAG-3′	Cuylen et al., 2011	Oligo SC42
CEN5 5′-AGCAGTATTAGATTTCCGAAAAGA-3′	This work	Oligo SC71
CEN5 5′-CGTTTAGTTTTTCTTTTCCTTTTCTTG-3′	This work	Oligo SC72
Recombinant DNA		
See Table S1		
Software and Algorithms		
X-ray Detector Software (XDS)	Kabsch, 2010	http://xds.mpimf-heidelberg.mpg.de/
autoSHARP	Vonrhein et al., 2007	https://www.globalphasing.com
Phenix suite	Adams et al., 2010	https://www.phenix-online.org/
CCP4 suite	Winn et al., 2011	http://www.ccp4.ac.uk/
SBGrid DEN/CNS	O'Donovan et al., 2012	https://portal.sbgrid.org/d/apps/den/
COOT v0.8.2	Emsley et al., 2010	https://www2.mrc-lmb.cam.ac.uk/personal/pemsley/coot/
PyMOL	Schrödinger, LLC	https://www.pymol.org/2/
ConSurf	Ashkenazy et al., 2016	http://bental.tau.ac.il/new_ConSurfDB/
APBS	Baker et al., 2001	http://www.poissonboltzmann.org/

CONTACT FOR REAGENT AND RESOURCE SHARING

Further information and requests for resources and reagents should be directed to and will be fulfilled by the Lead Contact, Christian H. Haering (christian.haering@embl.de).

EXPERIMENTAL MODEL AND SUBJECT DETAILS

Cell Lines

HeLa Kyoto H2B-mCherry cells (Neumann et al., 2010) were cultivated in DMEM (Life Technologies) containing 10% FBS (Life Technologies), 1% PenStrep (Invitrogen), and 1% glutamine (Invitrogen) at 37°C, 5% CO_2.

Yeast Strains

Saccharomyces cerevisiae strains are derived of W303. Genotypes are listed in the Table S3.

Bacterial Strains

Proteins for crystallography and biochemistry were expressed in *Escherichia coli* Rosetta (DE3) pLysS cells (Merck, 70954) pre-grown at 37°C and grown at 18°C for induction in 2 × TY or Terrific Broth (TB) medium.

METHOD DETAILS

Protein Expression and Purification

Expression of *Ct* Ycs4, *Ct* Ycs4–Brn1, or *Ct* $Smc2_{hd}$–$Brn1_N$ constructs (see Table S1) was induced for 18 h from pET-MCN vectors (Romier et al., 2006) in *Escherichia coli* Rosetta (DE3) pLysS (Merck) grown at 18°C in 2 × TY medium or M9 minimal medium (for NMR). The *Ct* $Smc4_{hd}$–$Brn1_C$ constructs were expressed in Sf21 cells using the Multibac expression system (Fitzgerald et al., 2006). Cells were lysed by sonication at 4°C in lysis buffer (50 mM TRIS-HCl pH 7.5, 200–500 mM NaCl, 20 mM imidazole, 5 mM β-mercaptoethanol) containing cOmplete protease inhibitor cocktail tablets without EDTA (cOm–EDTA, Roche). The lysate was cleared by centrifugation at 45,000 × g_{max} and loaded onto Ni-Sepharose 6FF (GE Healthcare). After washing with 30–40 column volumes (cv) lysis buffer, proteins were eluted in 7–10 cv elution buffer (lysis buffer plus 300 mM imidazole). The eluate was dialyzed overnight in dialysis buffer (25 mM TRIS-HCl pH 7.5, 200–300 mM NaCl, 1 mM DTT) at 4°C and amino-terminal His_6-tags were cleaved by addition of TEV protease, where applicable.

Ct $Smc2_{hd}$–$Brn1_N$ was loaded onto a Superdex 200 26/60 column (GE Healthcare) equilibrated in SEC-buffer (25 mM TRIS-HCl pH 7.5, 200 mM NaCl, 1 mM DTT). Peak fractions were pooled and diluted with low-salt buffer (25 mM TRIS-HCl pH 7.5, 100 mM NaCl, 1 mM DTT) to a final salt concentration of 100 mM NaCl and loaded onto a 1.7-mL Source S (GE Healthcare) cation exchange column pre-equilibrated with low-salt buffer. After washing with 3–5 cv low-salt buffer, proteins were eluted by increasing NaCl concentrations to 1 M in a linear gradient of 60 mL. Peak fractions were pooled and concentrated by ultrafiltration (Vivaspin 10,000 MWCO, Sartorius).

Ct $Smc4_{hd}$–$Brn1_C$ was loaded onto a Superdex 200 26/60 column (GE Healthcare) equilibrated in SEC-buffer (25 mM TRIS-HCl pH 7.5, 300 mM NaCl, 1 mM DTT). Peak fractions were pooled and diluted with low-salt buffer (25 mM TRIS-HCl pH 7.5, 100 mM NaCl, 1 mM DTT) to a final salt concentration of 100 mM NaCl and loaded onto a 6-mL Resource Q (GE Healthcare) anion exchange column pre-equilibrated with low-salt buffer. Flow through fractions containing *Ct* $Smc4_{hd}$–$Brn1_C$ were pooled and concentrated by ultrafiltration (Vivaspin 10,000 MWCO, Sartorius).

Ct Ycs4 and *Ct* Ycs4–Brn1 were diluted with low-salt buffer (25 mM TRIS-HCl pH 7.5, 100 mM NaCl, 1 mM DTT) to a final salt concentration of 150 mM NaCl and loaded onto a 6 mL RESOURCE Q (GE Healthcare) anion exchange column pre-equilibrated with low-salt buffer. After washing with 3–5 cv low-salt buffer, proteins were eluted by increasing NaCl concentrations to 1 M in a linear gradient of 60 mL. Peak fractions were pooled and loaded onto a Superdex 200 26/60 column (GE Healthcare) equilibrated in SEC-buffer (25 mM TRIS-HCl pH 7.5, 500 mM NaCl, 1 mM DTT). Peak fractions were pooled and concentrated by ultrafiltration (Vivaspin 30,000 MWCO, Sartorius).

His_6-tagged *Ct* Brn1 fragments spanning residues 225–512 or 336–512 where co-expressed and co-purified with untagged *Ct* Ycs4 using the above protocol. Excess *Ct* Brn1 was separated from the *Ct* Ycs4–$Brn1_{Y4}$ complex during the size-exclusion chromatography step.

Ct $Smc2_{cc}$–$Brn1_N$ fusion protein (NMR) was dialyzed overnight in dialysis buffer (10 mM Na/K phosphate pH 6.5, 50 mM NaCl, 1 mM DTT) at 4°C and the amino-terminal His_6-tag was cleaved by addition of TEV protease before loading onto a Superdex 75 26/60 column (GE Healthcare) equilibrated in SEC-buffer (10 mM Na/K phosphate pH 6.5, 50 mM NaCl, 1 mM DTT). Peak fractions were pooled and concentrated by ultrafiltration (Vivaspin 10,000 MWCO, Sartorius).

Various *Ct* Brn1 fragments (see Table S1) were expressed as amino-terminal GST fusion constructs from a pGEX6P-1 vector (Smith and Johnson, 1988) as described above. Cells were lysed at 4°C by sonication in lysis buffer (50 mM TRIS-HCl pH 7.5, 500 mM NaCl, 2 mM DTT containing cOm–EDTA). The lysate was cleared by centrifugation at 45,000 × g_{max} and loaded onto Glutathione Sepharose 4B beads (GE Healthcare). The GST-fusion protein was eluted from the beads with lysis buffer containing 10 mM L-glutathione. The eluate was dialyzed and purified over RESOURCE Q as described above. Peak fractions were pooled and concentrated by ultrafiltration (Vivaspin 10,000 MWCO).

Condensin holocomplexes were expressed (St-Pierre et al., 2009) and purified as described previously (Kschonsak et al., 2017). YbbR-tagged holocomplexes were covalently coupled to Coenzyme A-ATTO488 (New England Biolabs) with Sfp synthase as

described previously (Ganji et al., 2018). Selenomethionine-labeled *Ct* Ycs4–Brn1$_{Y4}$ and *Ct* Smc2$_{hd}$–Brn1$_{N}$ were expressed applying methionine pathway inhibition (Doublié, 1997) and purified as described above.

Crystallization and Data Collection

Crystals of selenomethionine-labeled *Ct* Smc2$_{hd}$ (form I) (Table 1) were grown at 20°C by hanging-drop vapor diffusion. A volume of 1 μL protein *Ct* Smc2hd$_{2-224/983-1179}$–Brn1$_{112-204}$ (8 mg/mL in 25 mM TRIS-HCl 7.5, 100 mM NaCl, 1 mM DTT) was mixed with 1 μL crystallization solution (18% (w/v) PEG3350, 0.2 M Succinate pH 7.0, 2 mM $MnCl_2$). Crystals were cryo-protected by addition of crystallization solution containing 20% (v/v) glycerol before flash freezing in liquid nitrogen. Single-wavelength anomalous dispersion data were collected at a wavelength of 0.979 Å (peak) at beamline ID29, European Synchrotron Radiation Facility (ESRF, Grenoble, France) (de Sanctis et al., 2012). Data were processed with XDS and XSCALE (Kabsch, 2010).

Ct Smc2$_{hd}$ crystals (form II) (Table 1) were grown by hanging-drop vapor diffusion after mixing 1 μL protein Smc2$_{2–215/990–1179}$–Brn1$_{112-204}$ (12.7 mg/mL in 25 mM TRIS-HCl pH 7.5, 100 mM NaCl, 1 mM DTT) and 1 μL crystallization solution (10%–17% (w/v) PEG 8,000, 0.1 M Na cacodylate pH 6.0) at 20°C. Crystals were cryo-protected by addition of 20% (v/v) glycerol before flash freezing in liquid nitrogen. The dataset was collected at a wavelength of 0.976 Å at beamline ID29, ESRF (de Sanctis et al., 2012). Data were processed with XDS (Kabsch, 2010) and POINTLESS (Evans, 2011) and scaled with SCALA of the CCP4 suite (Evans and Murshudov, 2013; Winn et al., 2011).

Ct Smc4$_{hd}$–Brn1$_{C}$ crystals (Table 1) were grown by hanging-drop vapor diffusion after mixing 1 μL protein Smc4$_{264–466/1367–1542}$–Brn1$_{765-898}$ (6.5 mg/mL in 25 mM TRIS-HCl pH 7.5, 150 mM NaCl, 1 mM DTT, 1 mM $MgCl_2$, 1 mM ATPγS) and 1 μL crystallization solution (3% (v/v) EtOH, 0.1 M Na citrate pH 6.0, 1.5 M $LiSO_4$) at 20°C. Crystals were cryo-protected in 2 M $LiSO_4$ before flash freezing in liquid nitrogen. The dataset was collected at a wavelength of 1.000 Å at beamline ID29, ESRF (de Sanctis et al., 2012). Data were processed as described above.

Native and selenomethionine-labeled *Ct* Ycs4–Brn1$_{Y4}$ crystals (Table 1) were grown by sitting drop vapor diffusion after mixing 100 nL Ycs4$_{\Delta loops}$–Brn1$_{225–418}$ and 100 nL crystallization solution in an MRC 2-well plate (Hampton Research). Native crystals were harvested after 30 days (5 mg/mL in 10 mM TRIS–HCl pH 7.5, 200 mM NaCl, 1 mM DTT) with crystallization buffer (12% (w/v) PEG 8,000, 0.1 M ADA pH 6.8, 0.1 M NaCl) at 7°C. Selenomethionine-labeled crystals were harvested after 30 days (6 mg/mL in 10 mM TRIS-HCl pH 7.5, 200 mM NaCl, 1 mM DTT) with crystallization buffer (13% (w/v) PEG 8,000, 0.1 M ADA pH 7.1, 0.13 M NaCl) at 7°C. All crystals were flash frozen in liquid nitrogen after addition of 2 μL crystallization buffer containing 37% (v/v) PEG 400. Datasets of selenomethionine-labeled *Ct* Ycs4–Brn1$_{Y4}$ were collected at a wavelength of 0.966 Å at beamline MASSIF1 (ID30A-1), ESRF (Bowler et al., 2015; Svensson et al., 2015). The dataset of native *Ct* Ycs4–Brn1$_{Y4}$ was collected at a wavelength of 1.0 Å at beamline ID29, ESRF (de Sanctis et al., 2012). All datasets were processed as described above using AIMLESS (Evans and Murshudov, 2013).

Ct Ycs4–Brn1$_{Y4}$–Smc4$_{hd}$–Brn1$_{C}$ crystals (Table 1) were grown by sitting drop vapor diffusion after mixing 100 nL sample (10 mg/mL complex in 25 mM TRIS–HCl pH 7.5, 150 mM NaCl, 1 mM DTT) and 100 nL crystallization solution (0.1 M TRIS-HCl pH 8.5, 8% PEG 8,000, 1 mM TCEP) in an MRC 2-well plate (Hampton Research) at 7°C. Crystals were cryo-protected by addition of 2 μl crystallization buffer containing 37% (v/v) PEG 400 before flash freezing in liquid nitrogen. The dataset was collected at a wavelength of 0.976 Å at beamline ID30B, ESRF (Mueller-Dieckmann et al., 2015). Data were processed as described above using SCALA (Evans, 2006).

Structure Determination and Refinement

Single anomalous dispersion data for *Ct* Smc2$_{hd}$ (crystal form I), was used to locate 16 selenium sites with autoSHARP (Vonrhein et al., 2007) followed by site refinement, phasing, and density modification. An initial model was built using Phenix AutoBuild and manual adjustment in Coot (Emsley et al., 2010; Terwilliger et al., 2008). The model was further improved by iterative rounds of restrained refinement with phenix.refine and manual adjustment with Coot (Afonine et al., 2012; Emsley et al., 2010).

The *Ct* Smc2$_{hd}$ (crystal form II) structure was solved by molecular replacement with an adapted *Ct* Smc2$_{hd}$ (crystal form I) as search model using Phenix Phaser-MR (McCoy, 2007). The structure was finalized in iterative rounds of manual correction with Coot (Emsley et al., 2010) and restrained refinement with phenix.refine (Afonine et al., 2012).

The *Ct* Smc4$_{hd}$–Brn1$_{C}$ structure was solved by molecular replacement with an adapted *Sc* Smc1$_{hd}$–Scc1$_{C}$ structure (pdb 1W1W) as search model using Phenix Phaser-MR (McCoy, 2007). An initial model was built using Phenix AutoBuild and manual adjustments with Coot (Emsley et al., 2010; Terwilliger et al., 2008). The structure was further improved in iterative rounds of manual correction with Coot (Emsley et al., 2010) and restrained refinement with phenix.refine (Afonine et al., 2012).

Single anomalous dispersion data, merged from two independent datasets, and native data for *Ct* Ycs4–Brn1$_{Y4}$ were used to locate 34 selenium sites with Phenix AutoSol (Adams et al., 2010), followed by site refinement, phasing, and density modification. An initial model was built using Phenix AutoBuild and manual adjustment in Coot (Emsley et al., 2010; Terwilliger et al., 2008). The model was further improved by iterative rounds of manual adjustments with Coot (Emsley et al., 2010) and restrained refinements with phenix.refine (Afonine et al., 2012) against the anomalous dataset.

The *Ct* Ycs4–Brn1$_{Y4}$–Smc4$_{hd}$–Brn1$_{C}$ low-resolution co-structure was solved by a molecular replacement search with adapted *Ct* Ycs4–Brn1$_{Y4}$ and *Ct* Smc4$_{hd}$–Brn1$_{C}$ as individual search components using Phenix Phaser-MR (McCoy, 2007). The initial model was refined using the deformable elastic network (DEN) protocol with CNS over a grid-enabled web server hosted by SBGrid (O'Donovan et al., 2012; Schröder et al., 2010) using standard settings and the input structure as both starting and reference models (Brunger

et al., 2012). Out of the resulting models, the one with the lowest R_{free} value was used for a final round of manual adjustments with Coot (Emsley et al., 2010) and real-space refinement with phenix.refine (Afonine et al., 2012).

All structures were refined with hydrogens ('riding' model) and validated using MolProbity (Chen et al., 2010). Structures (Table 1) have the following Ramachandran statistics: *Ct* $Smc2_{hd}$ (crystal form I) favored 96.0%, outliers 0.2%; *Ct* $Smc2_{hd}$ (crystal form II) favored 99.0%, outliers 0%; *Ct* $Smc4_{hd}$–$Brn1_{C}$ favored 94.0%, outliers 0.5%; *Ct* Ycs4–$Brn1_{Y4}$ favored 92.0%, outliers 0.8%; *Ct* Ycs4–Brn1–$Smc4_{hd}$ favored 90.0%, outliers 0.1%.

Structures were visualized with PyMOL (Schrödinger, LLC). The electrostatic surface potential graph was created with APBS (Baker et al., 2001).

NMR spectroscopy and structure calculation

NMR experiments were recorded on Bruker AVIII NMR spectrometers operating at field strengths corresponding to proton Larmor frequencies of 600 and 800 MHz equipped with a cryogenic TXI probe. All spectra were acquired at 298 K, processed with NMRPipe (Delaglio et al., 1995), and analyzed using NMRview (Johnson and Blevins, 1994). Initial backbone assignments were achieved from TROSY-HNCA, -HN(CO)CA, -HNCACB and -HN(CO)CACB recorded on a ^{2}H-^{13}C-^{15}N labeled sample (Pervushin et al., 1997; Salzmann et al., 1998). Backbone and side-chain assignments were completed mainly on a set of 3D NOESY spectra – ^{1}H-NOESY-^{1}H,^{15}N-HSQC, (^{1}H),^{13}C-HMQC-NOESY-^{1}H,^{15}N-HSQC, ^{1}H,^{13}C-HMQC-NOESY-^{1}H and (^{1}H),^{13}C-HMQC-NOESY-^{1}H,^{13}C-HMQC. The same experiments were used for deriving NOE-based distance restraints to feed structure calculation using CNS1.2 (Brunger, 2007) and ARIA1.2 (Linge et al., 2003). Due to the size and for NMR disadvantageous tumbling behavior of coiled-coil proteins, conventional side chain assignment experiments yielded too low signal-to-noise. Therefore, side chain assignments have been achieved using the above listed NOESY-type experiments. Consequently, NOEs were thereby manually assigned followed by the iterative ARIA approach to quantify, merge, and decrease assignment ambiguities, with the (^{1}H),^{13}C-HMQC-NOESY-^{1}H,^{13}C-HMQC data included as a 4D peak list. Backbone torsion angles were determined from chemical shifts using TALOS+ (Shen et al., 2009). Structural quality after refinement of the ten lowest energy structures out of 100 calculated structures in iteration 8 was validated using PROCHECK (Laskowski et al., 1996) and WHATIF (Vriend, 1990) (Table S5).

Multiple-sequence alignments

Smc2, Smc4, Brn1 and Ycs4 sequences from 40 divergent species (10 animals, 10 plants, 10 yeasts, 10 protists; Table S2) were aligned with MAFFT (Katoh et al., 2002) using the Smith-Waterman local algorithm (L-INS-i) with default settings. The Ycs4 alignments were used to map surface sequence conservation with Consurf (relaxed conservation scores) (Ashkenazy et al., 2016). To account for the overall higher level of sequence conservation, the column scores of the Smc2, Smc4 or Brn1 alignments were calculated with ClustalX (Larkin et al., 2007) using a PAM 250 matrix, binned to 10 steps and mapped onto *Ct* $Smc2_{hd}$ or *Ct* Smc4 $_{hd}$ surface models (strict conservation scores).

ATP Hydrolysis Assays

Reactions (10 μL) were set up with 5 μM SMC head proteins, as indicated, in ATPase buffer (50 mM TRIS-HCl pH 7.5, 215 mM NaCl, 2% (v/v) glycerol, 10 mM $MgCl_2$, 5 mM ATP, 1.3 mM DTT and 33 nM [α^{32}P]-ATP; Hartmann Analytic). ATP hydrolysis reactions were initiated by addition of ATP and incubated at 30°C. A volume of 1.0 μL of the reaction mix was spotted onto PEI cellulose F TLC plates (Merck) every 3 min for a total of 15 min. The reaction products were resolved on TLC plates using 0.5 M LiCl and 1 M formic acid solution and detected by exposing the TLC plates to a phosphorimager screen and analysis on a Typhoon FLA 9,500 scanner (GE Healthcare). ATP hydrolysis rates were calculated from the ADP/ATP ratios from time points in the linear range of the reaction.

ATPase assays with condensin holocomplexes were carried out as described previously (Kschonsak et al., 2017).

Isothermal Titration Calorimetry

Ct $Smc2_{hd}$–$Brn1_{N}$ or *Ct* $Smc4_{hd}$–$Brn1_{C}$ proteins were dialyzed to ITC buffer 1 (25 mM TRIS-HCl pH 7.5, 200 mM NaCl, 1 mM $MgCl_2$) or ITC buffer 2 (25 mM TRIS-HCl pH 7.5, 200 mM NaCl, 10 mM $MgCl_2$, 2% glycerol, 0.5 mM DTT). ATP was dissolved in ITC buffer 1 or buffer 2. ATP was injected at a concentration of 340 μM into 37.5–42.0 μM protein at 25°C (buffer 1) or 190–400 μM into 23.6–40.0 μM protein at 20°C (buffer 2). For the interaction studies of *Ct* Brn1 and *Ct* Ycs4 or of *Ct* $Smc4_{hd}$-$Brn1_{C}$ and *Ct* Ycs4-$Brn1_{Y4}$, proteins were dialyzed against ITC buffer 3 (25 mM TRIS-HCl pH 7.5, 300 mM NaCl, 0.5 mM DTT) and injected at 25°C or 10°C.

ITC measurements were performed on a MicroCal iTC200 or a PEAQ-ITC microcalorimeter (Malvern Panalytical). ITC data were corrected for the dilution heat and fitted with the MicroCal Origin software package applying one set of binding sites model. Standard deviation values of the fit were calculated from the original data points.

Analytical Size-Exclusion Chromatography

For *Ct* constructs, aliquots of 80 μL of protein samples at a concentration of 15 μM where incubated (with 1 mM ATP where indicated) on ice for 15 min and injected onto a Superdex 200 Increase 3.2/300 column (GE Healthcare) and separated in a buffer containing 175 mM NaCl, 25 mM TRIS-HCl pH 7.5, 1 mM $MgCl_2$ and 1 mM DTT (and 100 μM ATP where indicated) at a flow rate of 0.05 mL/min using the ÄKTA Ettan System (GE Healthcare). Fractions of 100 μL were collected and analyzed by SDS-PAGE and Coomassie staining.

For *Sc* condensin holocomplexes, 20 μL aliquots at a concentration of ~3 μM were incubated 16 h on ice with 1.5 μg TEV protease in the presence of 1 mM EDTA, 0.2 mM PMSF and 0.01% (v/v) Tween-20. After adjustment to 1.0 μM condensin concentration and 125 mM NaCl, 50 mM KCl, 50 mM TRIS-HCl pH 7.5, 5 mM $MgCl_2$ and 1 mM DTT (with 1 mM ATP where indicated), 50 μL was injected onto a Superose 6 Increase 3.2/300 column (GE Healthcare) and separated in same buffer (with 100 μM ATP where indicated) at a flow rate of 0.05 mL/min using the ÄKTA Ettan System (GE Healthcare). Fractions of 100 μL were collected and protein precipitated with 10% (w/v) trichloroacetic acid before SDS-PAGE and silver staining.

GST Pulldown

40 μg of glutathione S-transferase (GST) fusion protein was incubated with 60 μg of each untagged protein and 30 μL Glutathione Sepharose 4B (GE Healthcare) in 200 mM NaCl, 50 mM TRIS-HCl pH 7.5, 1 mM DTT, 1 mM $MgCl_2$ in a total volume of 0.5 mL for 1 h at 4°C. The beads were gently centrifuged at 1,200 rpm for 3 min and washed 6 times with the same volume of buffer before boiling and analysis by SDS-PAGE.

Release of the amino-terminal Brn1fragment

Aliquots of 10-20 μL of ~3 μM *Sc* condensin holocomplexes with CoA-ATTO488-labeled Brn1 were treated 16 h on ice with 1.5 μg TEV protease in the presence of 1 mM EDTA, 0.2 mM PMSF and 0.01% (v/v) Tween-20. Next, 5.5 pmol of condensin was immobilized on 20 μL Protein A-coupled Dynabeads (ThermoFisher Scientific) that had been pre-bound to 3 μg anti-HA antibody (12CA5). Beads were washed four times with 50 mM TRIS-HCl pH 7.5, 125 mM NaCl, 50 mM KCl, 5 mM $MgCl_2$, 5% (v/v) glycerol, 1 mM DTT, 0.2 mM PMSF and 0.01% (v/v) Tween-20. Release of the Brn1 amino-terminal cleavage fragment was subsequently assayed by washing three times with same buffer (including 1 mM ATP where indicated) with 5 min incubation at 25°C each. The three washes were collected and protein precipitated with 10% (w/v) trichloroacetic acid. Proteins bound to beads were eluted in 2 × SDS loading buffer (100 mM TRIS–HCl pH 6.8, 4% (w/v) SDS, 20% glycerol (v/v) 0.2% (w/v) bromophenol blue, 0.2 M DTT) by heating to 65°C for 5 min. Individual proteins were resolved by SDS-PAGE and fluorescence was analyzed in-gel on a Typhoon FLA9500 imager (GE Healthcare) with a 473-nm laser and a 510-nm long pass filter.

Condensin Immunoprecipitation and Western Blotting

Immunoprecipitation of endogenous condensin complexes from yeast was performed as described previously (Kschonsak et al., 2017). Yeast strains were grown at 30°C in 2 L YPAD media to an OD_{600} of 1.0, harvested by centrifugation and lysed by cryogenic grinding (SPEX Freezer/Mill 6970) in lysis buffer (50 mM TRIS-HCl pH 8.0, 100 mM NaCl, 2.5 mM $MgCl_2$, 0.25% (v/v) Triton X-100, 1 mM DTT, 1 mM PMSF) containing 2 × cOm–EDTA. The lysate was cleared by centrifugation at 20,400 × g_{max} and incubated with 100 μL Protein A-coupled Dynabeads (ThermoFisher Scientific) that had been pre-bound to anti-PK (V5) tag antibody (Abd Serotec, MCA1360) for 2 h at 4°C. Beads were washed with IP buffer (50 mM TRIS-HCl pH 8.0, 100 mM NaCl, 1 mM DTT, 5 mM EDTA, 0.25% (v/v) Triton X-100) and eluted in 20 μL 2 × SDS loading buffer (100 mM TRIS-HCl pH 6.8, 4% (w/v) SDS, 20% glycerol (v/v) 0.2% (w/v) bromophenol blue, 0.2 M DTT) by heating to 90°C for 5 min, prior to SDS-PAGE and Coomassie staining or western blotting with antibodies against the PK (V5) tag (Abd Serotec, MCA1360), *Sc* Ycg1 (Piazza et al., 2014) or α-tubulin (TAT1) (Woods et al., 1989).

Bpa Crosslinking

Yeast strains expressing $Smc4_{bpa}$ constructs were generated by plasmid shuffle, replacing a *URA3*-based episomal plasmid containing a wild-type *SMC4* allele in a *smc4Δ* background strain with the *TRP1*-based pLH157 encoding *E. coli* TyrRS and tRNA CUA (Chen et al., 2007) with a *LEU2*-based centromeric plasmid containing an $SMC4_{bpa}$ allele with an amber stop codon at the indicated position.

For analysis by western blotting, yeast strains were grown in 25 mL –LEU–TRP synthetic drop-out media containing 1 mM *p*-benzoyl-L-phenylalanine (bpa; Bachem 4017646) at 30°C to an OD_{600} of 0.6. Cells were harvested by centrifugation, resuspended in 1 mL PBS in a Petri dish and exposed to a total of 5 J 365-nm light using a Stratalinker 2400 UV cross-linker (~25 min exposure time) at room temperature. Cells were collected by centrifugation, resuspended in 0.5 mL 100 mM NaOH and incubated for 10 min at room temperature. Cells were collected by centrifugation and lysed in SDS loading buffer (50 mM TRIS-HCl pH 6.8, 2% (w/v) SDS, 10% glycerol (v/v) 0.1% (w/v) bromophenol blue, 0.1 M DTT) by at 65°C for 5 min prior to SDS-PAGE and western blotting with antibodies against the PK (V5) tag (Abd Serotec, MCA1360) or the HA tag (Abcam, ab9110).

For analysis by mass spectrometry, cells were harvested from 1.5 L –LEU–TRP media containing 1 mM *p*-benzoyl-l-phenylalanine at an OD_{600} of 2.9 and resuspended in 250 mL PBS. One half of the sample was exposed in Petri dishes to 10 J 365-nm light (~50 min exposure time), whereas the other half was kept in the dark. Cells were harvested by centrifugation, washed with 45 mL lysis buffer (50 mM TRIS-HCl pH 8.0, 100 mM NaCl, 2.5 mM $MgCl_2$, 0.25 (v/v) % Triton X-100) and resuspended in 15 mL lysis buffer containing 1 mM DTT, 1 mM PMSF and 2 × cOm–EDTA before lysis by cryogenic grinding (SPEX Freezer/Mill 6970). Condensin complexes were immunoprecipitated as described above, using 100 μL Protein A-coupled Dynabeads that had been pre-bound to 10 μg anti-PK antibody. After elution and SDS-PAGE, gels were silver stained using a formaldehyde-free protocol. The cross-linked band and a band at the same height in the –UV control were excised for analysis by mass spectrometry.

Mass Spectrometry

Silver-stained bands were excised, chopped into small pieces and transferred to 0.5-mL tubes. For all following steps, buffers were exchanged by two consecutive 15 min incubation steps in 200 μL of acetonitrile, which was removed after each step. Proteins were reduced by the addition of 200 μL of 10 mM DTT, 100 mM $(NH_4)HCO_3$ at 56°C for 30 min and alkylated by the addition of 200 μL of 55 mM iodoacetamide, 100 mM $(NH_4)HCO_3$ for 20 min in the dark. A volume of 50 μL of 1 ng/μL trypsin in 50 mM $(NH_4)HCO_3$ was added and samples were incubated for 30 min on ice and then over night at 37°C. Gel pieces were sonicated for 15 min, spun down and the supernatant was transferred into a glass vial (VDS Optilab, 93908556). The gel pieces were washed once with 50 μL of an aqueous solution of 50% acetonitrile and 1% formic acid and sonicated for 15 min. The combined supernatants were dried and reconstituted in 10 μL of 0.1% (v/v) formic acid.

Peptides were separated using the nanoAcquity UPLC system (Waters) with nanoAcquity trapping (nanoAcquity Symmetry C18, 5 μm, 180 μm × 20 mm) and analytical (nanoAcquity BEH C18, 1.7μm, 75 μm × 200 mm) columns, which were coupled to an LTQ Orbitrap Velos (Thermo Fisher Scientific) using the Proxeon nanospray source. Peptides were loaded for 6 min using a constant flow of solvent A (0.1% formic acid) at 5 μL min^{-1}. Peptides were then separated via the analytical column using a constant flow of 0.3 μL min^{-1}. The percentage of solvent B (acetonitrile, 0.1% formic acid) was increased from 3 to 10% within 5 min, followed by an increase to 40% within 10 min. Eluting peptides were ionized with a Pico-Tip Emitter 360 μm OD × 20 μm ID (10 μm tip, New Objective), applying a spray voltage of 2.2 kV at 300°C. Peptides were analyzed with an Orbitrap Velos Pro system (Thermo). Full scan MS spectra with a mass range of 300–1,700 m/z were acquired in profile mode with a resolution of 30.000 and a filling time of 500 ms, applying a limit of 106 ions. The 15 most intense ions were fragmented in the LTQ using a normalized collision energy of 40%. Three times 104 ions were selected within 100 ms and fragmented upon accumulation of selected precursor ions. MS/MS data were acquired in centroid mode of multiple charged (2+, 3+, 4+) precursor ions. The dynamic exclusion list was restricted to 500 entries with a maximum retention period of 30 s and relative mass window of 10 ppm. In order to improve the mass accuracy, a lock mass correction using a background ion (m/z 445.12003) was applied.

Data were processed using IsobarQuant (Franken et al., 2015) and Mascot (v2.2.07), including carbamidomethyl (C), acetyl (N-term) and oxidation (M) modifications. The mass error tolerance for full scan MS spectra was set to 10 ppm and for MS/MS spectra to 0.02 Da. A maximum of 2 missed cleavages were allowed. A minimum of two unique peptides with a peptide length of at least seven amino acids and a false discovery rate below 0.01 were required on the peptide and protein level (Table S4).

ChIP-qPCR

ChIP-qPCR experiments were performed as described previously (Cuylen et al., 2011; Kschonsak et al., 2017). Yeast strains were grown in 42 mL YPAD at 30°C to an OD_{600} of 0.6 and fixed with 4.7 mL fixation buffer (9.5 mM TRIS-HCl pH 8.0, 19 mM NaCl, 0.095 mM EGTA, 3% (v/v) formaldehyde, 0.19 mM EDTA) for 30 min at 16°C. Fixation was stopped by addition of glycine to 125 mM (final concentration), followed by washing steps in PBS and PIPES buffer (100 mM PIPES-KOH pH 8.3). Cells were lysed by spheroplasting with 0.5 mg/mL zymolase T-100 (AMS Biotechnology) in HEMS buffer (100 mM HEPES-KOH pH 7.5, 1 mM EGTA, 1 mM $MgSO_4$, 1.2 M Sorbitol, 1 mM PMSF) containing cOm–EDTA, followed by resuspension of cells in 1.5 mL lysis buffer (50 mM HEPES-KOH pH 7.5, 140 mM NaCl, 1 mM EDTA, 1% (v/v) Triton X-100, 0.1% (w/v) sodium deoxycholate, 1 mM PMSF) containing cOm–EDTA. Chromatin was sheared by sonication to a length of ~500 bp using a Bioruptor UCD-200 (Diagenode) for 9 min, 'high level' setting (30 s on, 60 s off).

Lysates were cleared by centrifugation at 16,800 × g_{max} and pre-cleared with 50 μL Protein A Dynabeads (ThermoFisher Scientific) for 90 min at 4°C. 10% of the cleared lysate was used to check sonication, 12% was kept on ice as input sample. 2 μg anti-PK (V5) tag antibody (Abd Serotec MCA1360) was added to the remaining lysate and samples were incubated at 4°C for 16 h before addition of 100 μl Protein A Dynabeads (ThermoFisher Scientific) for another 4 h at 4°C. Beads were washed with lysis buffer, wash buffer (10 mM TRIS-HCl pH 8.0, 0.25 M LiCl, 0.5% (w/v) sodium deoxycholate, 1 mM EDTA, 1 mM PMSF) containing cOm–EDTA and TE buffer (10 mM TRIS-HCl pH 8.0, 1 mM EDTA) containing cOm–EDTA. Samples were eluted in 320 μl TES buffer (50 mM TRIS–HCl pH 8.0, 10 mM EDTA, 1% (w/v) SDS) at 65°C for 8 h. After addition of 30 μg RNaseA (Roche) for 90 min at 37°C and 200 μg Proteinase K (Roche) for 90 min at 65°C, DNA was purified via a spin column (QIAGEN) and eluted in 50 μl EB buffer.

qPCR reactions were set up for 5 μl of 1:5 and 1:25 dilutions for immunoprecipitated samples and 1:5, 1:50, 1:500 and 1:5,000 dilutions for input samples with SYBR green PCR Master mix (Applied Biosystems) and 5 μM qPCR primers (see Key Resources Table) on an Applied Biosystems 7,500 Fast Real-Time PCR System. Data were calculated from two independent experiments with two qPCR runs each.

Microscopy of Human Condensin Complexes

HeLa Kyoto H2B-mCherry cells (Neumann et al., 2010) were transiently transfected with pC1 FLAG-EGFP-NCAPH or SMC4-FLAG-EGFP as described previously (Kschonsak et al., 2017).

Images were analyzed with Fiji (Schindelin et al., 2012). First, background was subtracted using the rolling ball algorithm. Chromatin regions were segmented based on the mCherry fluorescence signal and the whole cell was segmented based on the bright field image. Cytoplasmic regions were selected after subtracting the areas of chromatin from the whole cell regions. Mean fluorescence intensities of EGFP images were measured for chromatin and cytoplasmic regions. Data were calculated from two independent experiments. Cells were tested for mycoplasma contamination.

QUANTIFICATION AND STATISTICAL ANALYSIS

Statistical details of experiments can be found in the figure legends or Method Details section.

DATA AND SOFTWARE AVAILABILITY

The accession numbers for the coordinate files reported in this paper are PDB: 6Q6E, 6QJ0, 6QJ1, 6QJ2, 6QJ3, 6QJ4. The accession number for the NMR chemical shifts and restraints reported in this paper is BMRB: 34336. Original image files are available at Mendeley Data: https://doi.org/10.17632/rk9hdmj8tk.1.

Molecular Cell, Volume *74*

Supplemental Information

Structural Basis of an Asymmetric Condensin ATPase Cycle

Markus Hassler, Indra A. Shaltiel, Marc Kschonsak, Bernd Simon, Fabian Merkel, Lena Thärichen, Henry J. Bailey, Jakub Macošek, Sol Bravo, Jutta Metz, Janosch Hennig, and Christian H. Haering

Molecular Cell, Volume *74*

Supplemental Information

Structural Basis of an Asymmetric

Condensin ATPase Cycle

Markus Hassler, Indra A. Shaltiel, Marc Kschonsak, Bernd Simon, Fabian Merkel, Lena Thärichen, Henry J. Bailey, Jakub Macošek, Sol Bravo, Jutta Metz, Janosch Hennig, and Christian H. Haering

SUPPLEMENTAL FIGURES

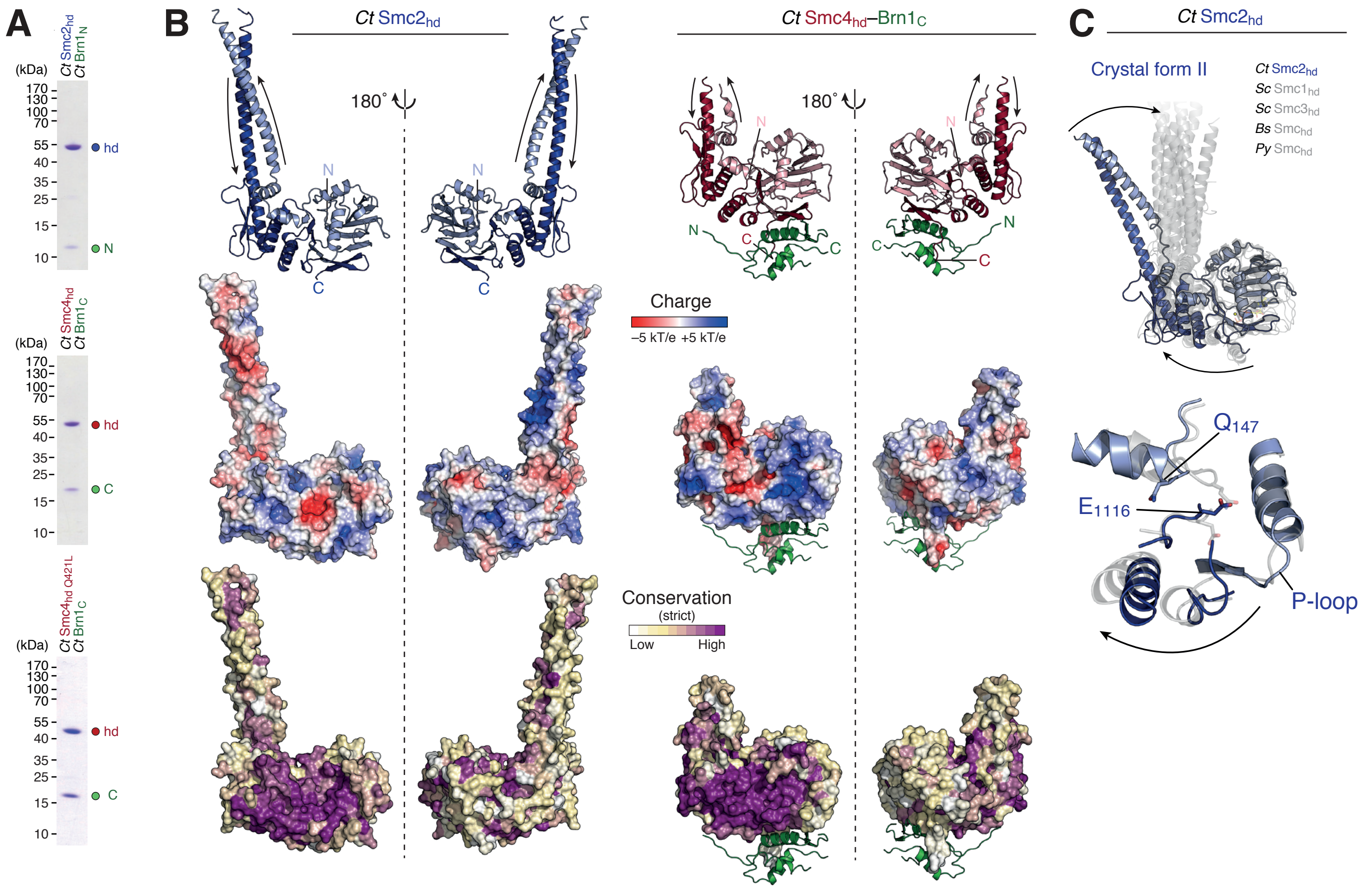

Figure S1. Structures of Smc2 and Smc4 ATPase Head Domains (related to Figure 1)

A Coomassie-stained SDS-PAGE lanes of wild-type *Ct* $Smc2_{hd}$–$Brn1_N$ and wild-type or Q-loop mutant *Ct* $Smc4_{hd}$–$Brn1_C$ complexes used for crystallization and ITC. **B** Cartoon, electrostatic surface potential and surface conservation models of *Ct* $Smc2_{hd}$ and *Ct* $Smc4_{hd}$–$Brn1_C$. **C** Structural alignment based on the RecA lobe of *Ct* $Smc2_{hd}$ (crystal form II) to ATPγS-bound structures of the *Sc* cohesin Smc1 (pdb 1W1W, C_α RMSD = 0.866) and Smc3 (pdb 4UX3, C_α RMSD = 2.880) or the nucleotide-free structures of *B. subtilis* (*Bs*) SMC (pdb 3ZGX, C_α RMSD = 2.866) and *P. yayanosii* (*Py*) SMC (pdb 5XEI, C_α RMSD = 0.988). Close-up views highlight the position of the conserved Q-loop glutamine and Walker B glutamate residues.

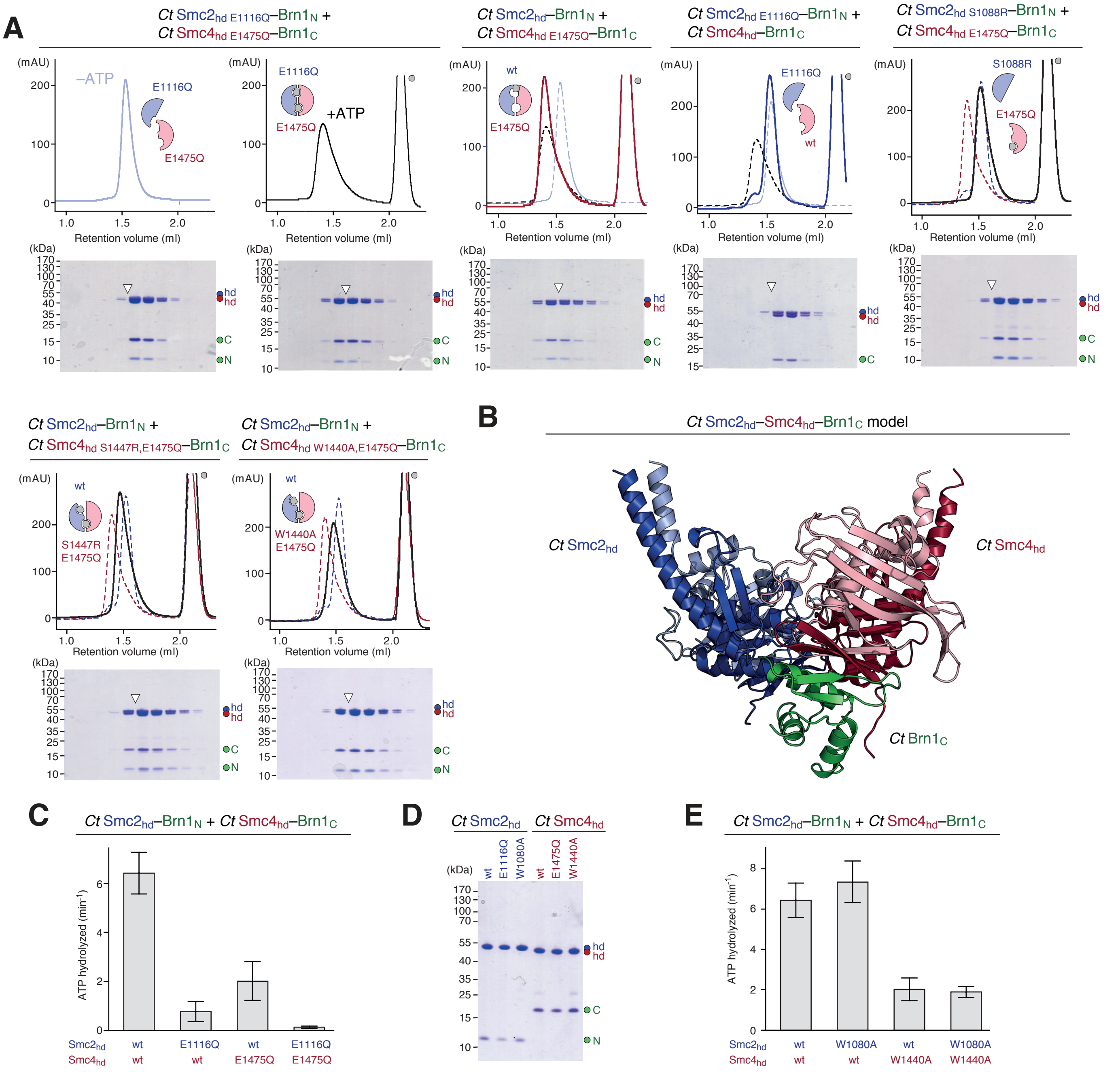

Figure S2. Smc2–Smc4 ATPase Head Dimerization (related to Figure 1)

A Size exclusion chromatography and Coomassie-stained SDS-PAGE analysis of elution fractions of wild-type, Walker B, signature motif or W-loop mutant combinations of *Ct* Smc2$_{hd}$–Brn1$_{N}$ and *Ct* Smc4$_{hd}$–Brn1$_{C}$. Dotted lines indicate elution profiles of *Ct* Smc2$_{hd\ E1116Q}$–Brn1$_{N}$ and *Ct* Smc4$_{hd\ E1475Q}$–Brn1$_{C}$ in the presence (black) or absence (light blue) of ATP, *Ct* Smc2$_{hd\ E1116Q}$–Brn1$_{N}$ and *Ct* Smc4$_{hd}$–Brn1$_{C}$ (blue) or *Ct* Smc2$_{hd}$–Brn1$_{N}$ and *Ct* Smc4$_{hd\ E1475Q}$–Brn1$_{C}$ (red) combinations. **B** Model of a *Ct* Smc2$_{hd}$–*Ct* Smc4$_{hd}$–Brn1$_{C}$ complex built on the ATPγS-dimerized *Sc* Smc1$_{hd}$–Scc1$_{C}$ homodimer structure (pdb 1W1W). **C** ATPase assays with wild-type or Walker B mutant combinations of *Ct* Smc2$_{hd}$–Brn1$_{N}$ and *Ct* Smc4$_{hd}$–Brn1$_{C}$ (mean ± SD of 3 independent experiments). **D** Coomassie-stained SDS-PAGE of wild-type, Walker B or W-loop mutants of *Ct* Smc2$_{hd}$–Brn1$_{N}$ and *Ct* Smc4$_{hd}$–Brn1$_{C}$. **E** ATPase assays with wild-type and W-loop mutant combinations of purified *Ct* Smc2$_{hd}$–Brn1$_{N}$ and *Ct* Smc4$_{hd}$–Brn1$_{C}$ complexes (mean ± SD of 3 independent experiments).

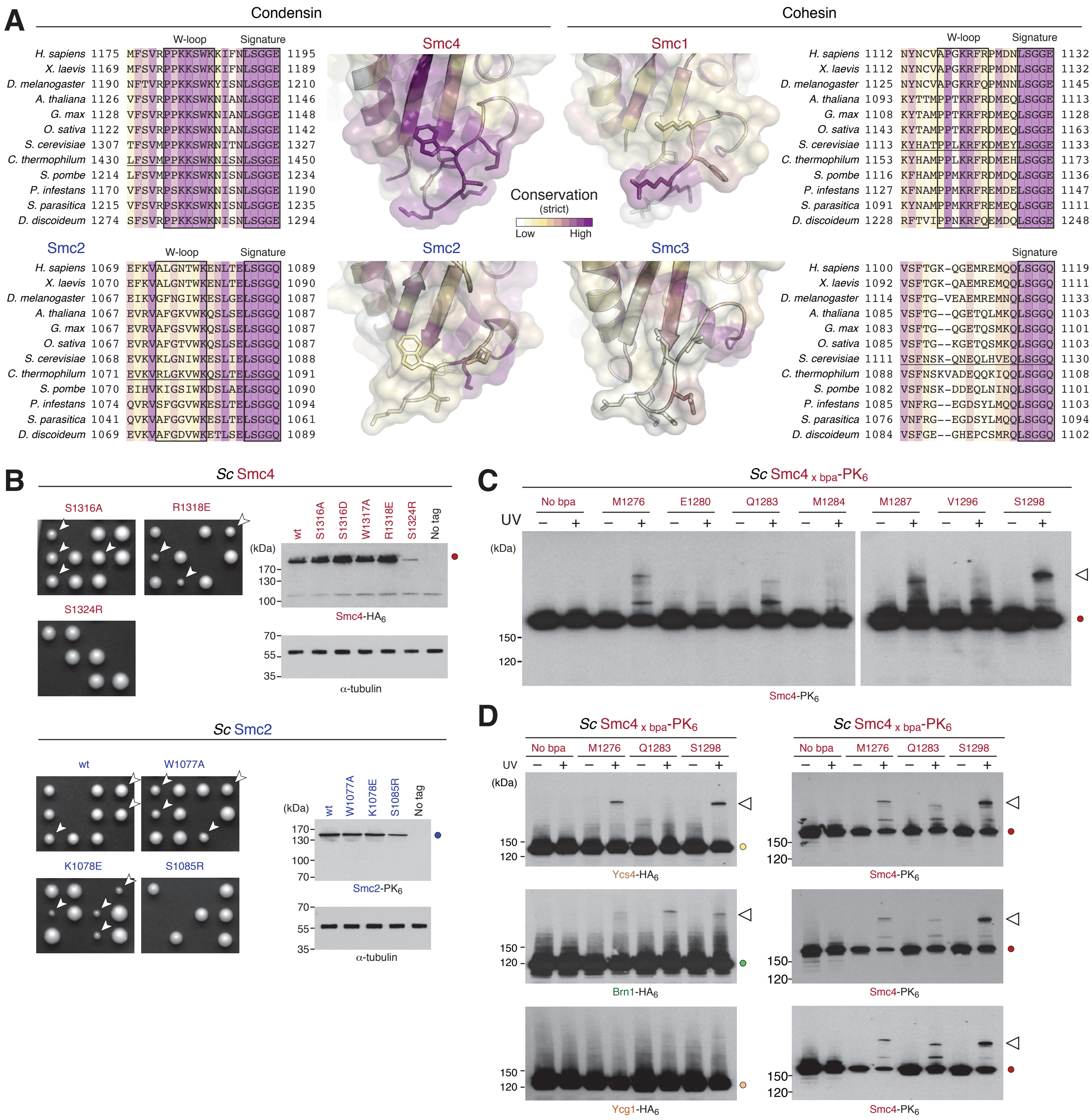

Figure S3. Conservation and Functional Analysis of the Smc4 W-loop (related to Figure 2)

A Partial alignment of W-loop sequences in Smc2 and Smc4 condensin and Smc1 and Smc3 cohesin subunits from 12 divergent species. Colors indicate conservation scores calculated from an alignment of sequences from 40 species (Table S1) and mapped onto the structures of *Ct* $Smc4_{hd}$, *Ct* $Smc2_{hd}$, *Sc* $Smc1_{hd}$ (pdb 1W1W) or *Sc* $Smc3_{hd}$ (pdb 4UX3). **B** Spores of diploid *S. cerevisiae SMC4/smc4Δ* strains expressing an ectopic HA_6-tagged copy of W-loop mutants $Smc4_{S1316A}$ (C4592), $Smc4_{R1318E}$ (C4590) or signature motif mutant $Smc4_{S1324R}$ (C4589) or *SMC2/smc2Δ* strains expressing an ectopic PK_6-tagged copy of W-loop mutants $Smc2_{W1077A}$ (C4567) or $Smc2_{K1078E}$ (C4608) or signature motif mutant $Smc2_{S1085R}$ (C4582) were dissected and incubated for 3 days at 30°C. Protein expression levels were tested by western blotting of whole cell extracts against HA or PK epitope tags. **C** Western blot analysis of Smc4 cross-linking products by probing against the PK epitope of whole cells extracts of strains (C4656, C4672, C4669, C4657, C4670, C4671, C4673, C4681) expressing Smc4-PK_6 with bpa substitutions at the indicated position before (–UV) or after (+UV) exposure to 365-nm light. **D** As in C, of strains co-expressing bpa-substituted versions of Smc4 with endogenously HA_6-tagged versions of Ycs4 (C4715, C4697, C4700, C4754), Brn1 (C4713, C4695, C4698, C4752) or Ycg1 (C4714, C4696, C4699, C4753).

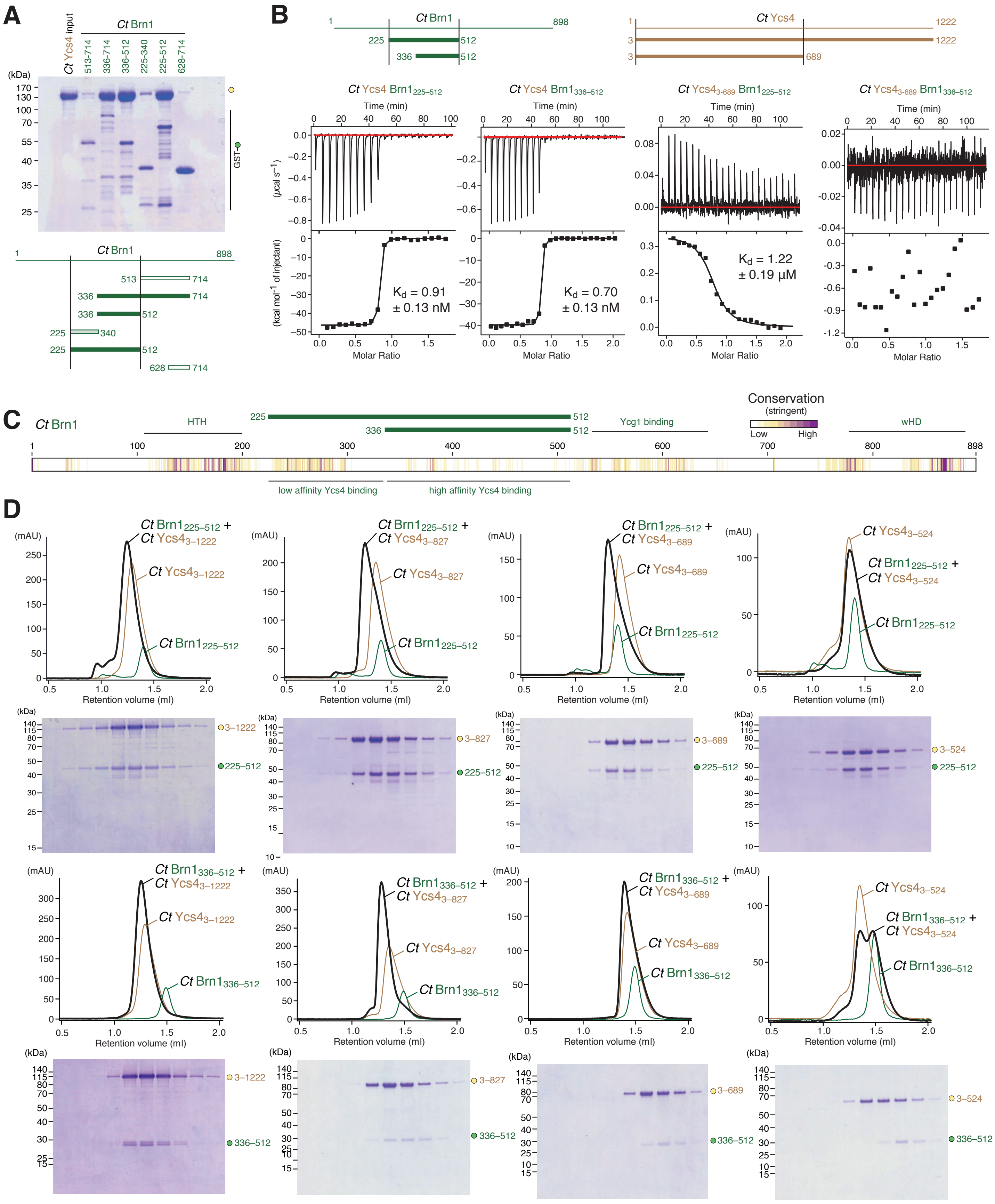
A
Ct Ycs4 input
Ct Brn1
513-714
336-714
336-512
225-340
225-512
628-714
(kDa)
GST-
Ct Brn1
1
898
B
Ct Brn1
Ct Ycs4
Ct Ycs4 Brn1225–512
Ct Ycs4 Brn1336–512
Ct Ycs43-689 Brn1225–512
Ct Ycs43-689 Brn1336–512
Time (min)
(µcal s−1)
(kcal mol−1 of injectant)
Kd = 0.91 ± 0.13 nM
Kd = 0.70 ± 0.13 nM
Kd = 1.22 ± 0.19 µM
Molar Ratio
C
Ct Brn1
HTH
Ycg1 binding
wHD
Conservation
(stringent)
Low
High
low affinity Ycs4 binding
high affinity Ycs4 binding
D
(mAU)
Retention volume (ml)
Ct Brn1225–512 + Ct Ycs43–1222
Ct Ycs43–1222
Ct Brn1225–512
Ct Brn1225–512 + Ct Ycs43–827
Ct Ycs43–827
Ct Brn1225–512 + Ct Ycs43–689
Ct Ycs43–689
Ct Ycs43–524
Ct Brn1225–512 + Ct Ycs43–524
(kDa)
3–1222
225–512
3–827
3–689
3–524
Ct Brn1336–512 + Ct Ycs43–1222
Ct Brn1336–512
Ct Brn1336–512 + Ct Ycs43–827
Ct Brn1336–512 + Ct Ycs43–689
Ct Brn1336–512 + Ct Ycs43–524
336–512

(Previous page)

Figure S4. Mapping of *Ct* Ycs4–Brn1 Interaction Domains (related to Figure 3)

A Binding of purified *Ct* Ycs4 protein (input) to glutathione beads pre-bound with various GST-*Ct* Brn1 fragments tested by SDS-PAGE and Coomassie staining of bound fractions. **B** ITC of the binding of full-length *Ct* $Ycs4_{3\text{-}1222}$ or truncated $Ycs4_{3\text{-}689}$ to *Ct* $Brn1_{225\text{-}512}$ or *Ct* $Brn1_{336\text{-}512}$ (fit ± error of the fit). **C** Graphical representation of the Brn1 sequence conservation. Colors indicate conservation scores calculated from an alignment of sequences from 40 species (Table S1). Brn1 stretches used for detailed Ycs4 mapping and regions interacting with other condensin subunits are highlighted. **D** Size exclusion chromatography of complexes formed between full-length or truncationed versions of *Ct* Ycs4 and *Ct* $Brn1_{225\text{-}512}$ or *Ct* $Brn1_{336\text{-}512}$. Coomassie-stained SDS-PAGE of elution fractions.

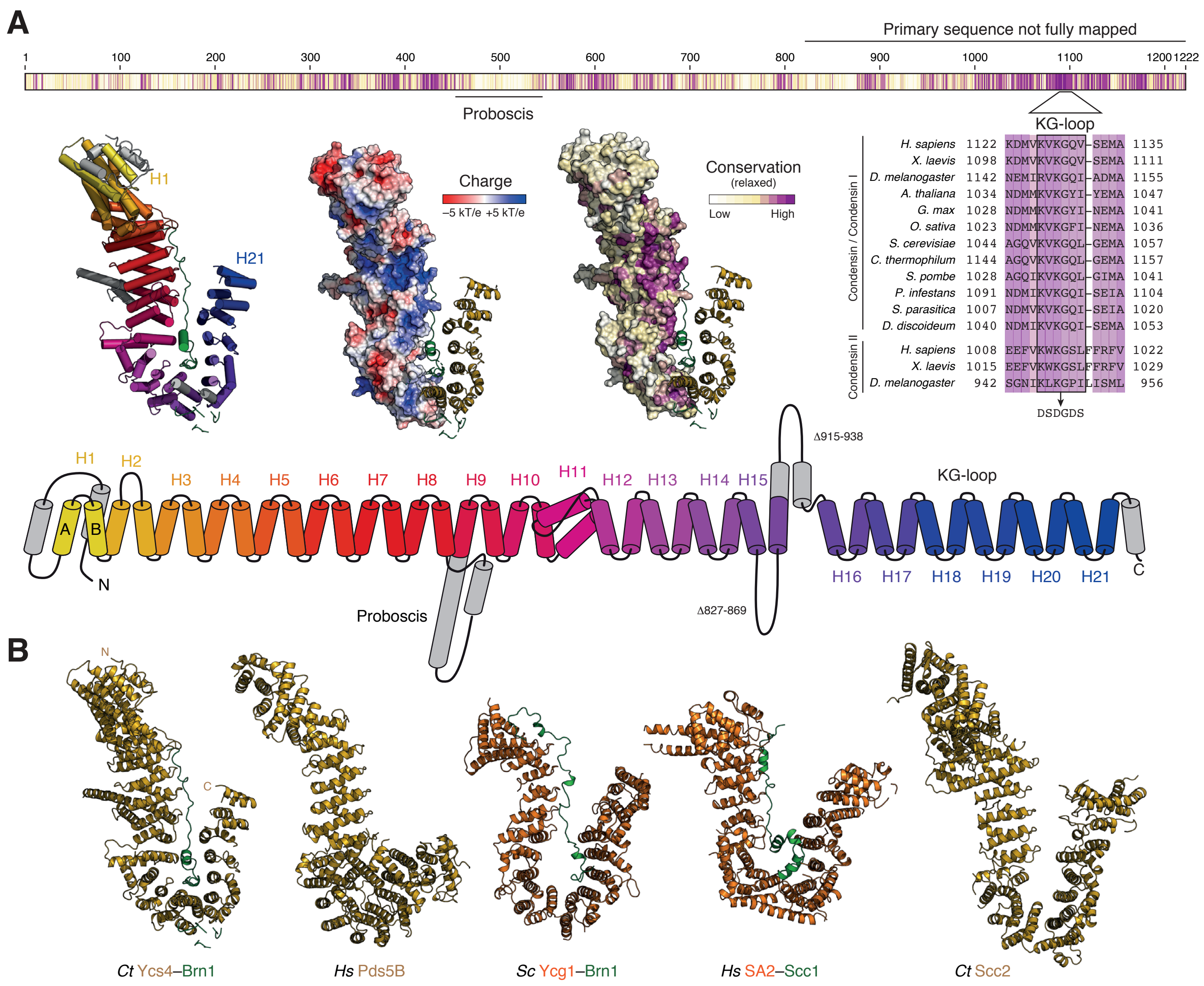

Figure S5. Structure of the *Ct* Ycs4–$Brn1_{Y4}$ Complex (related to Figure 3)

A Cartoon, electrostatic surface potential and surface conservation models of the *Ct* Ycs4–$Brn1_{Y4}$ complex. Surfaces were not built for the carboxy-terminal portion of Ycs4 due to the crystallographic disorder of this part of the complex. The colored box indicates conservation scores in Ycs4 calculated from an alignment of sequences from 40 species and the positions of the Proboscis and conserved KG-loop are highlighted (Table S1). The cartoon model indicates the arrangement of the 21 HEAT-repeat motifs in *Ct* Ycs4 (H1–H21). **B** Side-by-side comparison of the *Ct* Ycs4–Brn1 structure to structures of the *Hs* Pds5 HEAT-repeat subunit and the *Hs* SA2–Scc1 HEAT-repeat-kleisin complex of cohesin (pdb 5HDT, 4PJU), the *Ct* Ycg1–Brn1 HEAT-repeat-kleisin complex of condensin (pdb 5OQQ) and the *Ct* Scc2 subunit of the cohesin loading complex (pdb 5T8V).

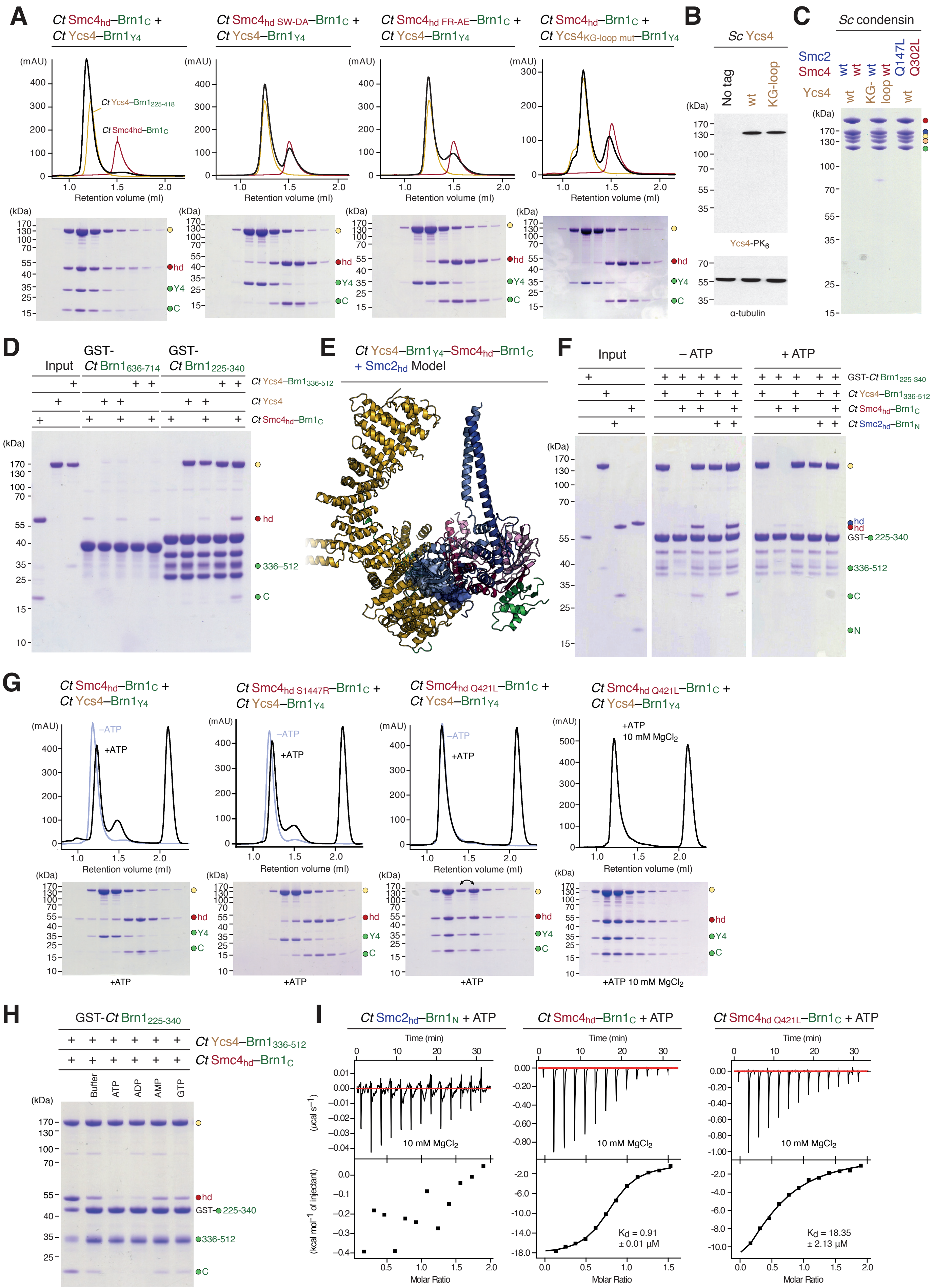

A
Ct Smc4hd–Brn1C + Ct Ycs4–Brn1Y4
Ct Smc4hd SW-DA–Brn1C + Ct Ycs4–Brn1Y4
Ct Smc4hd FR-AE–Brn1C + Ct Ycs4–Brn1Y4
Ct Smc4hd–Brn1C + Ct Ycs4KG-loop mut–Brn1Y4
Ct Ycs4–Brn1225-418
Ct Smc4hd–Brn1C
(mAU)
Retention volume (ml)
(kDa)
hd
Y4
C
B
Sc Ycs4
No tag
wt
KG-loop
Ycs4-PK6
α-tubulin
C
Sc condensin
Smc2
Smc4
Ycs4
wt
KG-loop
Q147L
Q302L
D
Input
GST-Ct Brn1636-714
GST-Ct Brn1225-340
Ct Ycs4–Brn1336-512
Ct Ycs4
Ct Smc4hd–Brn1C
336–512
E
Ct Ycs4–Brn1Y4–Smc4hd–Brn1C + Smc2hd Model
F
Input
– ATP
+ ATP
GST-Ct Brn1225-340
Ct Ycs4–Brn1336-512
Ct Smc4hd–Brn1C
Ct Smc2hd–Brn1N
GST-225-340
336-512
N
G
Ct Smc4hd–Brn1C + Ct Ycs4–Brn1Y4
Ct Smc4hd S1447R–Brn1C + Ct Ycs4–Brn1Y4
Ct Smc4hd Q421L–Brn1C + Ct Ycs4–Brn1Y4
Ct Smc4hd Q421L–Brn1C + Ct Ycs4–Brn1Y4
–ATP
+ATP
+ATP 10 mM MgCl2
H
GST-Ct Brn1225-340
Ct Ycs4–Brn1336-512
Ct Smc4hd–Brn1C
Buffer
ATP
ADP
AMP
GTP
I
Ct Smc2hd–Brn1N + ATP
Ct Smc4hd–Brn1C + ATP
Ct Smc4hd Q421L–Brn1C + ATP
Time (min)
(µcal s−1)
(kcal mol−1 of injectant)
10 mM MgCl2
Molar Ratio
Kd = 0.91 ± 0.01 µM
Kd = 18.35 ± 2.13 µM

(Previous page)

Figure S6. ATP-dependent Ycs4–Brn1$_{Y4}$ release from Smc2$_{hd}$–Brn1$_N$ (related to Figure 4)

A Size exclusion chromatography of complexes formed between *Ct* wild-type, Smc4 W-loop mutant (Smc4$_{S1439D, W1440A}$), Smc4 D-loop mutant (Smc4$_{F1482A, R1483D}$) or Ycs4 KG-loop mutant (Ycs4$_{KG\text{-}loop}$) versions of Smc4$_{hd}$–Brn1$_C$ with Ycs4–Brn1$_{225\text{-}418}$. **B** *Sc* Ycs4–PK$_6$ protein expression levels tested by western blotting of whole cell extracts against the PK epitope tag (strains C5003, C5005, C5007). **C** Coomassie-stained SDS-PAGE of wild-type, Ycs4 KG-loop and Smc2–Smc4 Q-loop mutant *Sc* condensin holocomplexes used for ATPase assays. **D** Binding of combinations of purified *Ct* Ycs4–Brn1$_{336\text{-}512}$, *Ct* Ycs4 and *Ct* Smc4hd–Brn1$_C$ proteins (input) to glutathione beads pre-bound with GST-*Ct* Brn1$_{636\text{-}714}$ or GST-*Ct* Brn1$_{225\text{-}340}$ tested by SDS-PAGE and Coomassie staining of bound fractions. **E** Model of the *Ct* Ycs4–Brn1$_{Y4}$–Smc4$_{hd}$–Brn1$_C$ complex bound to *Ct* Smc2$_{hd}$ based on the ATPɣS-dimerized *Sc* Smc1$_{hd}$–Scc1$_C$ homodimer structure (pdb 1W1W). Steric clashes are indicated by blue surfaces. **F** Binding of purified *Ct* Ycs4–Brn1$_{336\text{-}512}$, *Ct* Ycs4, *Ct* Smc2$_{hd}$–Brn1$_N$ and *Ct* Smc4$_{hd}$–Brn1$_C$ protein combinations (input) to glutathione beads pre-bound with GST-*Ct* Brn1$_{225\text{-}340}$ in the absence (–ATP) or presence of 600 μM ATP (+ATP) tested by SDS-PAGE and Coomassie staining of bound fractions. **G** Size exclusion chromatography of complexes formed between *Ct* Ycs4–Brn1$_{Y4}$ and wild-type, signature motif mutant (Smc4$_{hd\ S1447R}$) or Q-loop mutant (Smc4$_{hd\ Q421L}$) versions of *Ct* Smc4$_{hd}$–Brn1$_C$ in the absence (–ATP) or presence of nucleotide (+ATP), or in the presence of ATP and 10 mM $MgCl_2$. Coomassie-stained SDS-PAGE of elution fraction in the presence of ATP. **H** Effect of different nucleotides on the interaction between *Ct* Smc4–Brn1$_C$ and *Ct* Ycs4–Brn1$_{336\text{-}512}$ immobilized on glutathione beads via its binding to GST-*Ct* Brn1$_{225\text{-}340}$. **I** ITC in buffer containing 10 mM $MgCl_2$ of ATP binding by wild-type *Ct* Smc2$_{hd}$–Brn1$_N$ and wild-type or Q-loop mutant *Ct* Smc4$_{hd}$–Brn1$_C$ (fit ± error of the fit).

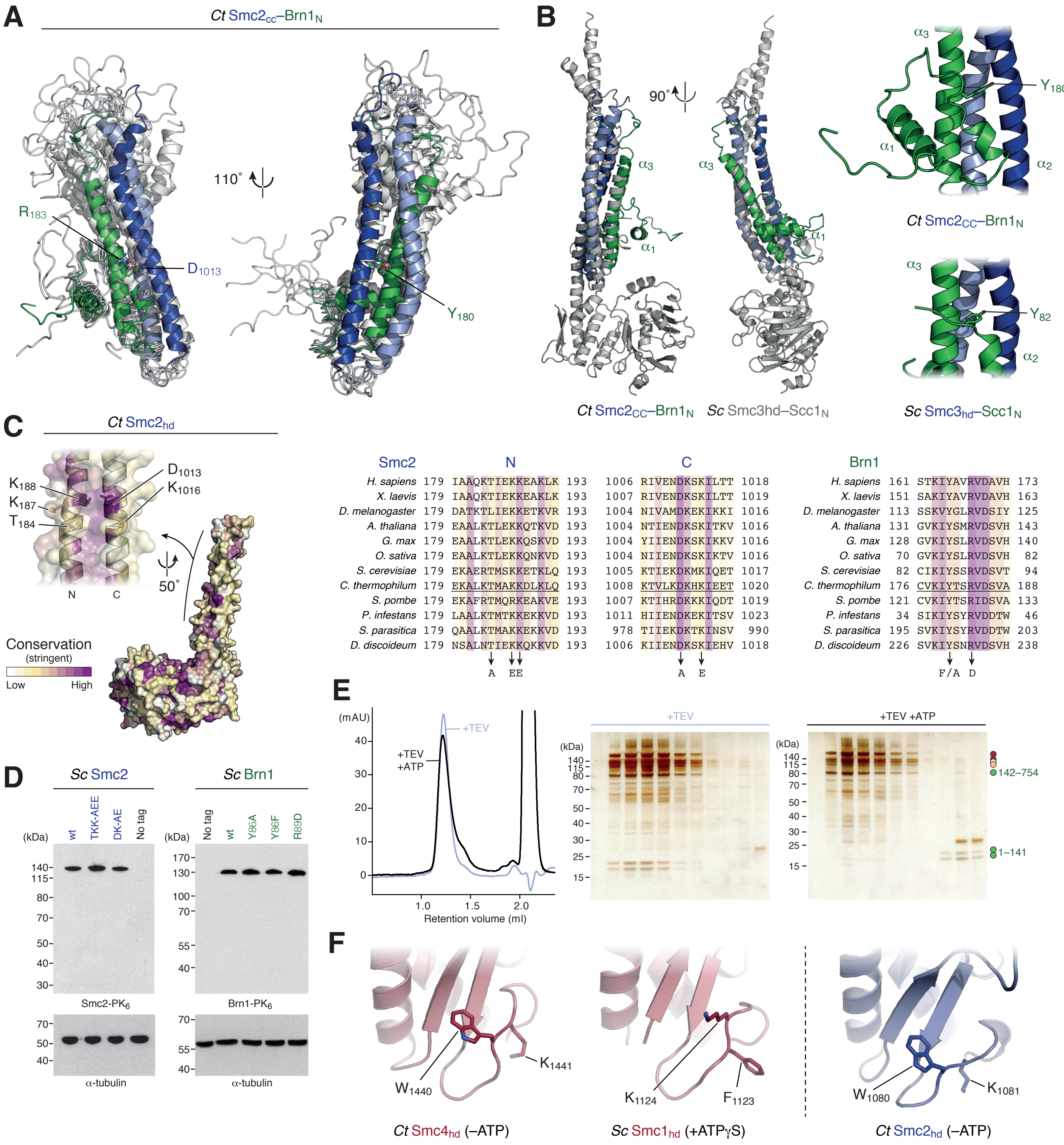

Figure S7. Structure and ATP-dependent release of the $Smc2_{cc}$–$Brn1_N$ from condensin (related to Figures 5 and 6)

A Ensemble of the 10 lowest energy NMR conformers of *Ct* $Smc2_{cc}$–$Brn1_N$. **B** Superimposition of the lowest energy *Ct* $Smc2_{CC}$–$Brn1_N$ NMR structure onto the *Sc* Smc3hd–$Scc1_N$ crystal structure (pdb 4UX3), using Smc2 coiled coil and Brn1 α_3 helices as guide. Close-up views highlight the intercalation of the conserved kleisin tyrosine residues between the SMC coiled coils. **C** Partial alignment of Smc2 amino-terminal (left) and carboxy-terminal (middle) neck regions as well as $Brn1_N$ α_3 regions from 12 divergent species. Colors indicate conservation scores calculated from an alignment of sequences from 40 species (Table S1). **D** Expression levels of wild-type (C5277) and mutant (C5278, C5279) versions of Smc2–PK_6 or of wild-type (C5239) and mutant (C5261–C5263) versions of Brn1–PK_6 in budding yeast probed by western blotting of whole cell extracts against the PK epitope tag. **E** Size exclusion chromatography of purified *Sc* condensin complexes cleaved by TEV protease at position 141 in Brn1 in the absence of nucleotide or presence of ATP. Silver-stained SDS-PAGE of TCA-precipitated elution fractions. **F** Comparison of W-loop residue conformations in nucleotide-free condensin *Ct* Smc2 and *Ct* Smc4 and ATPγS-bound *Sc* Smc1 (pdb 1W1W) head structures.

SUPPLEMENTAL TABLES

Table S1. Recombinant DNA (related to Figures 1 to 6)

1771	pETMCN 6×HIS-TEV-Brn1$_{112–204}$-Smc2$_{hd}$ (res.112–204 of *Ct* Brn1 and res. 2–224, 981–1179 of *Ct* Smc2)
2858	pETMCN 6×HIS-TEV-Brn1$_{112–204}$-Smc2$_{hd\ EQ}$ (res. 112–204 of *Ct* Brn1 and res. 2–224, 981–1179 of *Ct* Smc2 with E1116Q)
3138	pETMCN 6×HIS-TEV-Brn1$_{112–204}$-Smc2$_{hd\ QL}$ (res. 112–204 of *Ct* Brn1 and res. 2–224, 981–1179 of *Ct* Smc2 with Q147L)
3388	pETMCN 6×HIS-TEV-Brn1$_{112–204}$-Smc2$_{hd\ SR}$ (res. 112–204 of *Ct* Brn1 and res. 2–224, 981–1179 of *Ct* Smc2 with S1088R)
2936	pETMCN 6×HIS-TEV-Brn1$_{112–204}$-Smc2$_{hd\ WA}$ (res. 112–204 of *Ct* Brn1 and res. 2–224, 981–1179 of *Ct* Smc2 with W1080A)
1911	pETMCN 6×HIS-TEV-Brn1$_{112–204}$-Smc2$_{hd\ II}$ (res. 112–204 of *Ct* Brn1 and res. 2–215, 990–1179 of *Ct* Smc2)
3427	pETMCN Brn1$_{112–204}$-Smc2$_{hd}$-6×HIS (res. 112–204 of *Ct* Brn1 and res. 2–224, 981–1179 of *Ct* Smc2)
3428	pETMCN Brn1$_{112–204}$-Smc2$_{hd\ TKK\text{-}AEE}$-6×HIS (res. 112–204 of *Ct* Brn1 and res. 2–224, 981–1179 of *Ct* Smc2 with T184A, K187E, K188E)
3426	pETMCN Brn1$_{112–204}$-Smc2$_{hd\ DK\text{-}AE}$-6×HIS (res. 112-204 of *Ct* Brn1 and res. 2–224, 981–1179 of *Ct* Smc2 with D1013A, K1016E)
3577	pETMCN Brn1$_{112–204,\ R183D}$-Smc2$_{hd}$-6×HIS (res. 112–204 of *Ct* Brn1 with R183D and res. 2–224, 981–1179 of *Ct* Smc2)
3578	pETMCN Brn1$_{112\text{-}204,\ Y180A}$-Smc2$_{hd}$-6×HIS (res. 112–204 of *Ct* Brn1 with Y180A and res. 2–224, 981–1179 of *Ct* Smc2)
3627	pETMCN Brn1$_{112\text{-}204,\ Y180F}$-Smc2$_{hd}$-6×HIS (res. 112–204 of *Ct* Brn1 with Y180F and res. 2–224, 981–1179 of *Ct* Smc2)
2676	pETMCN 6×HIS–Brn1$_{112\text{-}204}$–Smc2$_{cc}$ fusion (res. 112–204 of *Ct* Brn1 and res. 981–1031, 170–224 of *Ct* Smc2)
2566	Multibac cre pIDC Brn1$_{765–898}$-pFL Smc4$_{hd}$-8×HIS (res. 765–898 of *Ct* Brn1 and res. 264–466, 1367–1542 *Ct* Smc4)
2775	Multibac cre pIDC Brn1$_{765–898}$-pFL Smc4$_{hd\ EQ}$-8×HIS (res. 765–898 of *Ct* Brn1 and res. 264–466, 1367–1542 of *Ct* Smc4 with E1475Q)
3224	Multibac cre pIDC Brn1$_{765–898}$-pFL Smc4$_{hd\ QL}$-8×HIS (res. 765–898 of *Ct* Brn1 and res. 264–466, 1367–1542 of *Ct* Smc4with Q421L)
3580	Multibac cre pIDC Brn1$_{765–898}$-pFL Smc4$_{hd\ EQ,\ SR}$-8×HIS (res. 765–898 of *Ct* Brn1 and res. 264–466, 1367–1542 of *Ct* Smc4 with E1475Q, S1447R)
2944	Multibac cre pIDC Brn1$_{765–898}$-pFL Smc4$_{hd\ EQ,\ WA}$-8×HIS (res. 765–898 of *Ct* Brn1 and res. 264–466, 1367–1542 of *Ct* Smc4 with E1475Q, W1440A)
3405	Multibac cre pIDC Brn1$_{765–898}$-pFL Smc4$_{hd\ SR}$-8×HIS (res. 765–898 of *Ct* Brn1 and res. 264–466, 1367–1542 of *Ct* Smc4 with S1447R)
3406	Multibac cre pIDC Brn1$_{765–898}$-pFL Smc4$_{hd\ SD,\ WA}$-8×HIS (res. 765–898 of *Ct* Brn1 and res. 264–466, 1367–1542 of *Ct* Smc4 with S1439D, W1440A)
3404	Multibac cre pIDC Brn1$_{765–898}$-pFL Smc4$_{hd\ FA,\ RD}$-8×HIS (res. 765–898 of *Ct* Brn1 and res. 264–466, 1367–1542 of *Ct* Smc4 with F1482A, R1483D)
2943	Multibac cre pIDC Brn1$_{765–898}$-pFL Smc4$_{hd\ WA}$-8×HIS (res. 765–898 of *Ct* Brn1 and res. 264–466, 1367–1542 of *Ct* Smc4 with W1440A)
1896	pETMCN 6×HIS-TEV-Ycs4$_{3–1222}$ (res. 3–1222 of *Ct* Ycs4)
2319	pETMCN 6×HIS-TEV-Ycs4$_{3–827}$ (res. 3–827 of *Ct* Ycs4)
2322	pETMCN 6×HIS-Ycs4$_{3–689}$ (res. 3–689 of *Ct* Ycs4)
1857	pETMCN 6×HIS-Ycs4$_{3–518}$ (res. 3–518 of *Ct* Ycs4)
2221	pETMCN 6×HIS-Brn1$_{225–512}$-Ycs4 (res. 225–512 of *Ct* Brn1 and res. 3–1222 of *Ct* Ycs4)
2220	pETMCN 6×HIS-Brn1$_{336–512}$-Ycs4 (res. 336–512 of *Ct* Brn1 and res. 3–1222 of *Ct* Ycs4)
2777	pETMCN 6×HIS-Brn1$_{225–418}$-Ycs4$_{\Delta loops}$ (res. 225–418 of *Ct* Brn1 and res. 3–828, 869–915, 939–1222 of *Ct* Ycs4)
3176	pETMCN 6×HIS-Brn1$_{225–418}$-Ycs4$_{\Delta loops,\ KG\text{-}loop\ mut}$ (res. 225–418 of *Ct* Brn1 and res. 3–828, 869–915, 939–1222 of *Ct* Ycs4 with K1094D, V1095S, K1096D, Q1098D, L1099S)
1786	pGEX6PI GST-HRV3C-Brn1$_{225–340}$ (residues 225–340 of *Ct* Brn1)
1877	pGEX6PI GST-HRV3C-Brn1$_{225–512}$ (residues 225–512 of *Ct* Brn1)
1785	pGEX6PI GST-HRV3C-Brn1$_{336–512}$ (residues 336–512 of *Ct* Brn1)
1780	pGEX6PI GST-HRV3C-Brn1$_{336–714}$ (residues 336–714 of *Ct* Brn1)
1784	pGEX6PI GST-HRV3C-Brn1$_{513–714}$ (residues 513–714 of *Ct* Brn1)
1879	pGEX6PI GST-HRV3C-Brn1$_{636–714}$ (residues 636–714 of *Ct* Brn1)
2648	2µ pGAL7 Smc4-3×StrepII, pGAL10 Smc2, pGAL1 Brn1-12×HIS-3×HA, TRP1 (*Sc* Smc4, Smc2, Brn1)
3029	2µ pGAL7 Smc4$_{Q302L}$-3×StrepII, pGAL10 Smc2$_{Q147L}$, pGAL1 Brn1–12×HIS–3×HA, TRP1 (*Sc* Smc4 with Q302L, Smc2 with Q147L, Brn1)
2843	2µ pGAL1 Ycg1, pGAL10 Ycs4, URA3 (*Sc* Ycg1, Ycs4)
3308	2µ pGAL1 Ycg1, pGAL10 Ycs4$_{KG\text{-}loop\ mut}$, URA3 (*Sc* Ycg1, Ycs4 with K1049D, V1050S, K1051D, Q1053D, L1054S)
2845	2µ pGAL10 Ycs4, URA3 (*Sc* Ycs4)

Continued on next page

Table S1. Recombinant DNA (related to Figures 1 to 6) *continued from previous page*

3314	2µ pGAL7 Smc4-3×StrepII, pGAL10 Smc2, pGAL1 Brn1(ybbR$_{13–23}$, 3×TEV$_{141}$)-12×HIS-3×HA, TRP1 (*Sc* Smc4, Smc2, Brn1 with ybbR tag replacing res. 13–23, 3×TEV site inserted at res. 141)
3349	2µ pGAL7 Smc4$_{E1352Q}$-3×StrepII, pGAL10 Smc2$_{E1113Q}$, pGAL1 Brn1(ybbR$_{13–23}$, 3×TEV$_{141}$)-12×HIS-3×HA, TRP1 (*Sc* Smc4 with E1352Q, Smc2 with E1352Q, Brn1 with ybbR tag replacing res. 13–23, 3×TEV site inserted at res.141)
3371	2µ pGAL7 Smc4$_{Q302L}$-3×StrepII, pGAL10 Smc2$_{Q147L}$, pGAL1 Brn1(ybbR$_{13–23}$, 3×TEV$_{141}$)-12×HIS-3×HA, TRP1 (*Sc* Smc4 with Q302L, Smc2 with Q147L, Brn1 with ybbR tag replacing res. 13–23, 3×TEV site inserted at res. 141)
3378	2µ pGAL7 Smc4$_{S1324R}$-3×StrepII, pGAL10 Smc2$_{S1085R}$, pGAL1 Brn1(ybbR$_{13–23}$, 3×TEV$_{141}$)-12×HIS-3×HA, TRP1 (*Sc* Smc4 with S1324R, Smc2 with S1085R, Brn1 with ybbR tag replacing res. 13–23, 3×TEV site inserted at res. 141)
3373	2µ pGAL7 Smc4-3×StrepII, pGAL10 Smc2, pGAL1 Brn1(ybbR13–23, 1×TEV$_{141}$, 1×TEV$_{373}$)-12×HIS-3×HA, TRP1 (*Sc* Smc4, Smc2, Brn1 with ybbR tag replacing residues 13–23, 1×TEV sites inserted at res. 141 and 373)
1999	pSMC4-FLAG-EGFP (*Hs* SMC4)
1725	pSMC4-FLAG-EGFPWA (*Hs* SMC4 with W1185A)
1732	pSMC4-FLAG-EGFPSD, WA (*Hs* SMC4 with S1184D, W1185A)
1455	pFLAG-EGFP-NCAPD2 (*Hs* NCAPD2)
3649	pFLAG-EGFP-NCAPD2 (*Hs* NCAPD2 with K1384D, V1385S, K1386D, Q1388D, V1389S)
1288	6.4kb (plasmid backbone with *E. coli* ori and amp^{R})

Table S2. Uniprot Identifiers for Sequence Alignments (related to Figures 2, S1, S3, S5 and S7)

				Smc2	Smc4	Brn1	Ycs4
Animals	Vertebrates	Mammals	*Homo sapiens*	O95347	Q9NTJ3	Q15003	Q15021
			Mus musculus	Q8CG48	Q8CG47	Q8C156	Q8K2Z4
		Birds	*Gallus gallus*	Q90988	Q8AWB9	A0A1D5P3B2	A0A1L1RND5
		Reptiles	*Chelonia mydas*	M7AYY6	M7B6H5	M7BB33	M7BQD0
		Amphibians	*Xenopus laevis*	P50533	P50532	O13067	Q9YHY6
		Fish	*Danio rerio*	B8A5K9	E7FGC2	A1L231	B0V123
	Arthropods	Insects	*Drosophila melanogaster*	Q7KK96	Q9V3A7	P91663	Q9VAJ1
	Nematodes		*Trichinella spiralis*	A0A0V1B7P9	A0A0V1B923	E5SDR4	A0A0V1BS59
			Caenorhabditis elegans	Q09591	Q20060	G5EGE9	Q9U2M1
	Flatworms		*Schistosoma mansoni*	G4VP73 (gap)	G4LZF5	G4VCQ8	G4VHF7
Plants	Eudicots	Mustard family	*Arabidopsis thaliana*	Q9C5Y4	Q9FJL0	Q564K3	Q9M1J4
		Mallow family	*Theobroma cacao*	A0A061FZA3	A0A061EEP1	A0A061FCQ2	A0A061EVA8
		Myrtle family	*Eucalyptus grandis*	A0A059DKA1	A0A059AQS4	A0A059DDB4	A0A059DI45
		Pea family	*Glycine max*	I1M0W9	I1LWK5	I1KFG2	K7K8M4
		Grape family	*Vitis vinifera*	F6HAI6	D7SXB5	D7TEB5	F6HLQ7
		Nightshade family	*Solanum lycopersicum*	A0A097PJ88	K4BBV4	K4CF69	K4D2L4
	Monocots	Grass family	*Oryza sativa*	Q8GU55	Q8L6H8	B9EXC2	Q7XAM6
	Basal magnoliophyta	Amborella family	*Amborella trichopoda*	W1PPA4	W1PTT0	W1NZC1	U5CSJ8
	Mosses		*Physcomitrella patens*	A9S6L3	A9RDI2	A9TD22	A9TB92
	Red algae		*Galdieria sulphuraria*	M2XLH5	M2XMB5	M2XK80	M2WUI9
Fungi	Ascomycetes	Saccharomycetes	*Saccharomyces cerevisiae*	P38989	Q12267	P38170	Q06156
			Candida albicans	A0A1D8PI59	Q5A4Y2	A0A1D8PMC8	A0A1D8PI01
		Sordariomycetes	*Chaetomium thermophilum*	G0S5H7	G0S2G2	G0SBJ6	G0SB82
			Neurospora crassa	Q7S9M2	Q7S1T6	Q7SCS0	Q7S1I4
		Eurotiomycetes	*Emericella nidulans*	Q5B0N1	C8V7U2	Q5B5B5	C8VHB5
			Neosartorya fumigata	Q4X159	Q4WIE1	Q4WG60	A0A0J5ST09
		Dothideomycetes	*Botryosphaeria parva*	R1E6Z9	R1GIF9	R1GN90	R1GUT6
		Schizosaccharomycetes	*Schizosaccharomyces pombe*	P41003	P41004	Q9Y7R3	O94679
	Basidiomycetes		*Cryptococcus neoformans*	J9VUT8	J9VMA0	Q5K864	Q5K972
	Microsporidians		*Encephalitozoon cuniculi*	Q8SSJ9	Q8SRK4	Q8SWA2	Q8SSE2
Protists	Choanoflagellates		*Salpingoeca rosetta*	F2UFL3	F2U3W0	F2TXB9	F2UF62
	Amoebozoa	Dictyostelium	*Dictyostelium discoideum*	Q54PK4	Q54LV0	Q54DR4	Q54B17
	Alveolates	Ciliates	*Emericella nidulans*	Q22ST6	Q6PUA5	Q24BA4	Q233H3
	Stramenopiles	Diatoms	*Neosartorya fumigata*	B7GAL2	B5Y5J8	B7G0X0	B5Y4W4
			Thalassiosira pseudonana	B8BQT7	B8CCA2	B8BWW0	B8BQQ4
		Oomycetes	*Phytophthora infestans*	D0NY62	D0NXB1	D0MV02	D0P2X5
			Saprolegnia parasitica	A0A067CIY4	A0A067CB22	A0A067CV32	A0A067CDW8
	Cryptomonads		*Guillardia theta*	L1J804	L1JUU7	L1INS6	L1JQ47
	Euglenozoa	Kinetoplasts	*Trypanosoma brucei*	Q389U3	Q38CG6	Q57ZI0	Q38F58
			Leishmania major	Q4QJG2	Q4QC62	Q4QJ31	Q4QF22

Table S3. Yeast Genotypes (related to Figures 2, 4, 5, 6, S3, S6 and S7)

C4568	MATa/α, smc4::HIS3/SMC4, ura3::SMC4-HA$_6$::URA3/ura3
C4592	MATa/α, smc4::HIS3/SMC4, ura3::SMC4$_{S1316A}$-HA$_6$::URA3/ura3
C4595	MATa/α, smc4::HIS3/SMC4, ura3::SMC4$_{S1316D}$-HA$_6$::URA3/ura3
C4570	MATa/α, smc4::HIS3/SMC4, ura3::SMC4$_{W1317A}$-HA$_6$::URA3/ura3
C4590	MATa/α, smc4::HIS3/SMC4, ura3::SMC4$_{R1318E}$-HA$_6$::URA3/ura3
C4589	MATa/α, smc4::HIS3/SMC4, ura3::SMC4$_{S1324R}$-HA$_6$::URA3/ura3
C4564	MATa/α, smc2::hphMX4/SMC2, trp1::SMC2-PK$_6$::TRP1/trp1
C4567	MATa/α, smc2::hphMX4/SMC2, trp1::SMC2$_{W1077A}$-PK$_6$::TRP1/trp1
C4608	MATa/α, smc2::hphMX4/SMC2, trp1::SMC2$_{K1078E}$-PK$_6$::TRP1/trp1
C4582	MATa/α, smc2::hphMX4/SMC2, trp1::SMC2$_{S1085R}$-PK$_6$::TRP1/trp1
C4656	MATa, smc4::natMX, [YCplac111 SMC4-PK$_6$ LEU2], [pLH157::TRP1]
C4672	MATa, smc4::natMX, [YCplac111 SMC4$_{M1276amb}$-PK$_6$ LEU2], [pLH157 TRP1]
C4669	MATa, smc4::natMX, [YCplac111 SMC4$_{E1280amb}$-PK$_6$ LEU2], [pLH157 TRP1]
C4657	MATa, smc4::natMX, [YCplac111 SMC4$_{Q1283amb}$-PK$_6$ LEU2], [pLH157 TRP1]
C4670	MATa, smc4::natMX, [YCplac111 SMC4$_{M1284amb}$-PK$_6$ LEU2], [pLH157 TRP1]
C4671	MATa, smc4::natMX, [YCplac111 SMC4$_{M1287amb}$-PK$_6$ LEU2], [pLH157 TRP1]
C4673	MATa, smc4::natMX, [YCplac111 SMC4$_{V1296amb}$-PK$_6$ LEU2], [pLH157 TRP1]
C4681	MATa, smc4::natMX, [YCplac111 SMC4$_{S1298amb}$-PK$_6$ LEU2], [pLH157 TRP1]
C4715	MATa, smc4::natMX, YCS4-HA6::HIS3, [YCplac111 SMC4-PK$_6$ LEU2], [pLH157 TRP1]
C4697	MATa, smc4::natMX, YCS4-HA6::HIS3, [YCplac111 SMC4$_{M1276amb}$-PK$_6$ LEU2], [pLH157 TRP1]
C4700	MATa, smc4::natMX, YCS4-HA6::HIS3, [YCplac111 SMC4$_{Q1283amb}$-PK$_6$ LEU2], [pLH157 TRP1]
C4754	MATa, smc4::natMX, YCS4-HA6::HIS3, [YCplac111 SMC4$_{S1298amb}$-PK$_6$ LEU2], [pLH157 TRP1]
C4713	MATa, smc4::natMX, BRN1-HA6::HIS3, [YCplac111 SMC4-PK$_6$ LEU2], [pLH157 TRP1]
C4695	MATa, smc4::natMX, BRN1-HA6::HIS3, [YCplac111 SMC4$_{M1276amb}$-PK$_6$ LEU2], [pLH157 TRP1]
C4698	MATa, smc4::natMX, BRN1-HA6::HIS3, [YCplac111 SMC4$_{Q1283amb}$-PK$_6$ LEU2], [pLH157 TRP1]
C4752	MATa, smc4::natMX, BRN1-HA6::HIS3, [YCplac111 SMC4$_{S1298amb}$-PK$_6$ LEU2], [pLH157 TRP1]
C4714	MATa, smc4::natMX, YCG1-HA6::HIS3, [YCplac111 SMC4-PK$_6$ LEU2], [pLH157 TRP1]
C4696	MATa, smc4::natMX, YCG1-HA6::HIS3, [YCplac111 SMC4$_{M1276amb}$-PK$_6$ LEU2], [pLH157 TRP1]
C4699	MATa, smc4::natMX, YCG1-HA6::HIS3, [YCplac111 SMC4$_{Q1283amb}$-PK$_6$ LEU2], [pLH157 TRP1]
C4753	MATa, smc4::natMX, YCG1-HA6::HIS3, [YCplac111 SMC4$_{S1298amb}$-PK$_6$ LEU2], [pLH157 TRP1]
C5003	MATa/α, ycs4::kanMX6/YCS4, URA3/ura3
C5005	MATa/α, ycs4::kanMX6/YCS4, ura3::YCS4-PK$_6$::URA3/ura3
C5007	MATa/α, ycs4::kanMX6/YCS4, ura3::YCS4$_{K1048D,\ V1049S,\ K1050D,\ Q1052D,\ L1053S}$-PK$_6$::URA3/ura3
C5277	MATa/α, smc2::HIS3/SMC2, ura3::SMC2-PK$_6$::URA3/ura3
C5278	MATa/α, smc2::HIS3/SMC2, ura3::SMC2$_{T184A,\ K187E,\ K188E}$-PK$_6$::URA3/ura3
C5279	MATa/α, smc2::HIS3/SMC2, ura3::SMC2$_{D1010A,\ K1013E}$-PK$_6$::URA3/ura3
C4239	MATa/α, brn1::HIS3/BRN1, ura3::BRN1-PK$_6$::URA3/ura3
C5261	MATa/α, brn1::HIS3/BRN1, ura3::BRN1$_{Y86A}$-PK$_6$::URA3/ura3
C5262	MATa/α, brn1::HIS3/BRN1, ura3::BRN1$_{Y86F}$-PK$_6$::URA3/ura3
C5263	MATa/α, brn1::HIS3/BRN1, ura3::BRN1$_{R89D}$-PK$_6$::URA3/ura3

Continued on next page

Table S3. Yeast Genotypes (related to Figures 2, 4, 5, 6, S3, S6 and S7) *continued from previous page*

C4491	MATa, lys2::pGAL1 GAL4::LYS2, pep4::HIS3, bar1::hisG, [2μ, pGAL7 SMC4-(StrepII)$_3$, pGAL10 SMC2, pGAL1 BRN1-HA$_3$-His$_{12}$ TRP1], [2μ, pGAL1 YCG1, pGAL10 YCS4 URA3]
C4724	MATa, lys2::pGAL1 GAL4::LYS2, pep4::HIS3, bar1::hisG, [2μ, pGAL7 SMC4$_{Q302L}$-(StrepII)$_3$, pGAL10 SMC2$_{Q147L}$, pGAL1 BRN1-HA$_3$-His$_{12}$ TRP1], [2μ, pGAL1 YCG1, pGAL10 YCS4 URA3]
C5050	MATa, lys2::pGAL1 GAL4::LYS2, pep4::HIS3, bar1::hisG, [2μ, pGAL7 SMC4-(StrepII)$_3$, pGAL10 SMC2, pGAL1 BRN1-HA$_3$-His$_{12}$ TRP1], [2μ, pGAL1 YCG1, pGAL10 YCS4$_{K1048D, V1049S, K1050D, Q1052D, L1053S}$ URA3]
C4896	MATa, lys2::pGAL1 GAL4::LYS2, pep4::HIS3, bar1::hisG, [2μ, pGAL7 SMC4-(StrepII)$_3$, pGAL10 SMC2, pGAL1 BRN1(TEV141)$_3$-HA$_3$-His$_{12}$ TRP1], [2μ, pGAL1 YCG1, pGAL10 YCS4 URA3]
C5066	MATa, lys2::pGAL1 GAL4::LYS2, pep4::HIS3, bar1::hisG, [2μ, pGAL7 SMC4-(StrepII)$_3$, pGAL10 SMC2, pGAL1 ybbR(12-24)-BRN1(TEV141)$_3$-HA$_3$-His$_{12}$ TRP1], [2μ, pGAL1 YCG1, pGAL10 YCS4 URA3]
C5125	MATa, lys2::pGAL1 GAL4::LYS2, pep4::HIS3, bar1::hisG, [2μ, pGAL7 SMC4$_{Q302L}$-(StrepII)$_3$, pGAL10 SMC2$_{Q147L}$, pGAL1 ybbR$_{12–24}$-BRN1(TEV141)$_3$-HA3-His$_{12}$ TRP1], [2μ, pGAL10 YCS4,, pGAL1 YCG1 URA3]
C5139	MATa, lys2::pGAL1 GAL4::LYS2, pep4::HIS3, bar1::hisG, [2μ, pGAL7 SMC4$_{S1324R}$-(StrepII)$_3$, pGAL10 SMC2$_{S1085R}$, pGAL1 ybbR$_{12–24}$-BRN1(TEV141)$_3$-HA$_3$-His$_{12}$ TRP1], [2μ, pGAL10 YCS4, pGAL1 YCG1 URA3]
C5142	MATa, lys2::pGAL1 GAL4::LYS2, pep4::HIS3, bar1::hisG, [2μ, pGAL7 SMC4$_{E1352Q}$-(StrepII)$_3$, pGAL10 SMC2$_{E1113Q}$, pGAL1 ybbR$_{12–24}$-BRN1(TEV141)$_3$-HA$_3$-His$_{12}$ TRP1], [2μ, pGAL10 YCS4, pGAL1 YCG1 URA3]
C5110	MATa, lys2::pGAL1 GAL4::LYS2, pep4::HIS3, bar1::hisG, [2μ, pGAL7 SMC4-(StrepII)$_3$, pGAL10 SMC2, pGAL1 ybbR$_{12–24}$-BRN1(TEV141)3-HA$_3$-His$_{12}$ TRP1], [2μ, pGAL10 YCS4 URA3]
C5122	MATa, lys2::pGAL1 GAL4::LYS2, pep4::HIS3, bar1::hisG, [2μ, pGAL7 SMC4-(StrepII)$_3$, pGAL10-SMC2, pGAL1 ybbR$_{12–24}$-BRN1(TEV141, TEV373)-HA$_3$-His$_{12}$ TRP1], [2μ, pGAL10 YCS4, pGAL1 YCG1 URA3]

Table S4. Mass Spectrometry Data (related to Figure 2E)

Protein Name	Uniprot Identifier	Mass (kDa)	Score	Number Peptides	Sequence Coverage (%)
**$Smc4_{S1298bpa}$ –UV**					
None					
**$Smc4_{S1298bpa}$ +UV**					
Sc Smc4	Q12267	162.1	2,236	59	35.4
Sc Ycs4	Q06156	132.9	1,638	38	26.1
Sc Brn1	P38170	86.2	392	10	13.9
Sc Smc2	P38989	133.8	272	5	4.6

Table S5. NMR Statistics (related to Figure 5)

**Experimental restraints**	
Distance restraints	
Total NOEs (unambiguous/ambiguous)	2527/317
Short range ($\|i–j\| \leq 1$)	1884/202
Medium range ($\|i–j\| < 5$)	265/66
Long range ($\|i–j\| > 5$)	378/49
Hydrogen bonds	61
Dihedral restraints (φ/ψ)	170/170
**Structural quality**	
Coordinate precision (Å, residues 58–80,130–160,167–193)	
Backbone (N, C_α, C')	0.65 ± 0.16
Heavy atoms	1.16 ± 0.15
Coordinate precision (Å, residues 16-46,51–56,58–80,130–160,167–193)	
Backbone (N, C_α, C')	0.79 ± 0.15
Heavy atoms	1.25 ± 0.15
Restraint RMSD	
Distance restraints (Å)	0.024 ± 0.005
Dihedral restraints (°)	0.91 ± 0.47
Deviation from idealized geometry	
Bond lengths (Å)	0.0032 ± 0.0001
Bond angles (°)	0.46 ± 0.02
**Ramachandran analysis (%)**	
Favoured regions	91.1 ± 1.4
Allowed regions	8.0 ± 1.5
Generously allowed	0.4 ± 0.3
Disallowed	0.6 ± 0.5
**Whatcheck analysis**	
1st generation packing	–0.717 ± 0.311
2nd generation packing	–2.019 ± 0.342
Ramachandran plot appearance	–1.689 ± 0.427
Chi^{-1}/Chi^{-2} rotamer normality	–2.958 ± 0.385
Backbone conformation	–0.666 ± 0.435
